# RNF168 and USP10 regulate topoisomerase IIα function via opposing effects on its ubiquitylation

**DOI:** 10.1038/ncomms12638

**Published:** 2016-08-25

**Authors:** Kiran Kumar Naidu Guturi, Miyuki Bohgaki, Toshiyuki Bohgaki, Tharan Srikumar, Deborah Ng, Ramya Kumareswaran, Samah El Ghamrasni, Justin Jeon, Parasvi Patel, Mohamed Saad Eldin, Rob Bristow, Peter Cheung, Grant S. Stewart, Brian Raught, Anne Hakem, Razqallah Hakem

**Affiliations:** 1 Department of Medical Biophysics, Princess Margaret Cancer Centre, Ontario Cancer Institute, University Health Network, University of Toronto, Toronto, Ontario, Canada M5G 1L7; 2 Department of Biology, York University, Toronto, Ontario, Canada M3J 1P3; 3 School of Cancer Sciences, University of Birmingham, Birmingham B15 2TT, UK

## Abstract

Topoisomerase IIα (TOP2α) is essential for chromosomal condensation and segregation, as well as genomic integrity. Here we report that RNF168, an E3 ligase mutated in the human RIDDLE syndrome, interacts with TOP2α and mediates its ubiquitylation. RNF168 deficiency impairs decatenation activity of TOP2α and promotes mitotic abnormalities and defective chromosomal segregation. Our data also indicate that RNF168 deficiency, including in human breast cancer cell lines, confers resistance to the anti-cancer drug and TOP2 inhibitor etoposide. We also identify USP10 as a deubiquitylase that negatively regulates TOP2α ubiquitylation and restrains its chromatin association. These findings provide a mechanistic link between the RNF168/USP10 axis and TOP2α ubiquitylation and function, and suggest a role for RNF168 in the response to anti-cancer chemotherapeutics that target TOP2.

## Abstract (web summary)

The E3 ligase RNF168 is essential for the signalling of DNA double strand break and its mutations are associated with the RIDDLE syndrome. Here the authors identify TOP2a as substrate for RNF168 and USP10; providing a link between the RNF168/USP10 axis, TOP2a and the response to anti-cancer drugs that target TOP2.

## Graphical abstract

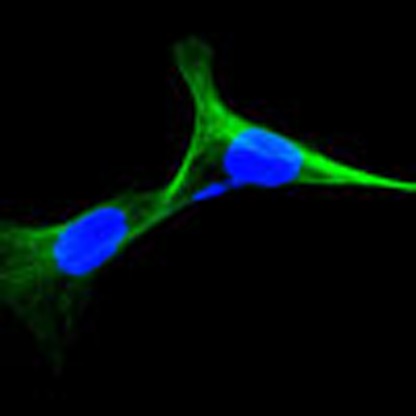

TOP2α is important for chromosome condensation and segregation owing to its role in DNA decatenation and the decatenation G2 checkpoint^1^,^2^. This enzyme forms a transient covalent linkage with DNA, known as the TOP2α-DNA cleavage complex, during S phase to resolve DNA supercoiling that occurs during DNA replication, recombination and transcription^1^,^2^,^3^. TOP2α functions by binding a DNA duplex and cleaving both strands to allow another intact double-stranded DNA to pass through the break. Following strand passage, TOP2α ligates the DNA double-strand break (DSB), and thus relieves DNA topological stress. Through its activities, and its role in the decatenation G2 checkpoint, TOP2α is critical for maintaining genomic integrity^4^,^5^,^6^.

Post-translational modifications of TOP2α, including ubiquitylation, SUMOylation and phosphorylation, have been implicated in the regulation of TOP2α localization, turnover and activity^1^,^2^,^3^. TOP2α ubiquitylation by BMI1/RING1A or APC/C-Cdh1 promotes its degradation^2^,^7^,^8^, while its ubiquitylation by BRCA1 stimulates its decatenation activity^9^, in addition to its proteasomal degradation^10^. Despite the importance of ubiquitylation on TOP2α function and steady-state levels, the mechanisms that mediate its deubiqyuitylation have not yet been identified.

The E3 ubiquitin ligase RNF168 is an important regulator of DSB repair^11^. This repair process is vital for maintaining genomic integrity; and its defects have been implicated in the pathogenesis of a number of human diseases such as cancer^12^,^13^. At the sites of DSBs, RNF168 ubiquitylates H2A type histones to promote the recruitment of downstream DNA damage response proteins, including BRCA1 (refs 11, 14, 15). Further underscoring the importance of this E3 ubiquitin ligase, biallelic mutations of this gene have been identified as the underlying cause of the human RIDDLE syndrome^16^,^17^,^18^. In keeping with this disease connection, *Rnf168* knockout mice exhibit many features of the human disorder, including increased radiosensitivity, immunodeficiency and genomic instability, which, in combination with the loss of p53, promotes earlier onset of cancer^19^.

To gain further insight into RNF168 functions, we used a proteomics approach to identify its interacting partners. Here, we report that RNF168 interacts with TOP2α to mediate its polyubiquitylation, and we further show that RNF168 deficiency confers resistance to ICRF-193, a TOP2 catalytic inhibitor, and etoposide (VP-16), a TOP2 poison and cytotoxic anti-cancer drug. We also report that RNF168 deficiency impairs TOP2α-chromatin association and its decatenation activity. Finally, we show that the deubiquitylase USP10 interacts with RNF168 and TOP2α, and restrains ubiquitylation of TOP2α as well as its chromatin binding. Collectively, our data reveal a novel and critical RNF168-dependent mechanism for fine-tuning TOP2α's decatenation activity through regulation of its ubiquitylation status. Our data also uncover USP10 as an important deubiquitylase for TOP2α.

## Results

### RNF168 is a novel interacting partner for TOP2α

**Figure 1:**
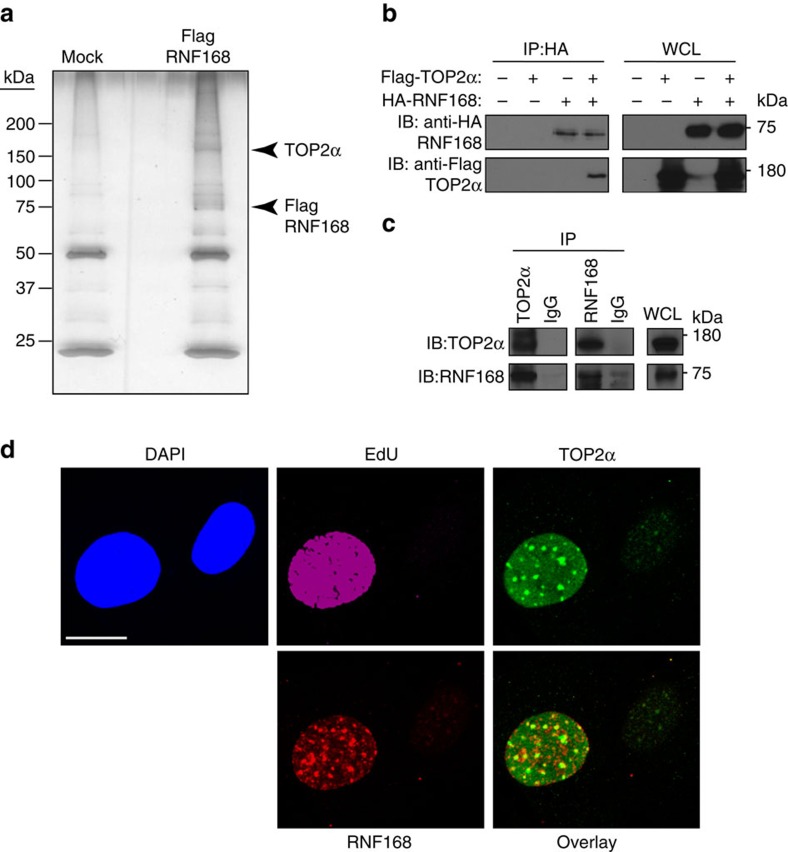
RNF168 interacts with TOP2α. (**a**) Identification of RNF168-associated proteins. A representative SDS–polyacrylamide gel electrophoresis of Flag-RNF168-associated proteins. Flag-tagged RNF168 was transfected in HEK293T cells and pull-down analysis was performed 48 h later. Protein bands were detected by silver staining. Protein bands were identified by mass spectrometry analysis following in-gel protease digestion. (**b**) HEK293T cells were transfected as indicated with HA-tagged RNF168 and Flag-TOP2α expression vectors. Cells were lysed and IP was performed using anti-Flag antibody. The resulting precipitates were subjected to IB analysis with the indicated antibodies. WCL, whole-cell lysate. (**c**) TOP2α, RNF168 and IgG (control) immunoprecipitates from HEK293T cells were examined by IB as indicated. (**b**,**c**) Data are representative of three independent experiments. (**d**) Cells treated with EdU were used for detection of localization patterns of TOP2α (Alexa Fluor 488) and RNF168 (Alexa Fluor 594) using confocal microscopy. Scale bar, 20 μm.

To further define the functions of the E3 ligase RNF168, we initially expressed Flag-tagged RNF168 in HEK293T cells and coupled Flag immunoprecipitation (IP) with SDS–polyacrylamide gel electrophoresis analysis and mass spectrometry to identify novel RNF168-binding proteins. We found a 170 kDa protein that was significantly enriched in the Flag-RNF168 immunoprecipitate, which we identified as TOP2α (Fig. 1a and Supplementary Table 1).

To confirm the physical association of RNF168 with TOP2α, we first co-transfected HEK293T cells with HA-tagged RNF168 and Flag-tagged TOP2α. Anti hemagglutinin (HA) immunoblot (IB) analysis of Flag-RNF168 immunoprecipitates from these transfected cells confirmed that RNF168 was pulled down by the TOP2α IP (Fig. 1b). Next, we examined the interaction between endogenous RNF168 and TOP2α. Consistent with our data using tagged proteins, reciprocal IPs of endogenous RNF168 and TOP2α from HEK293T cells showed that they interacted with one another (Fig. 1c).

We also examined using immunofluorescence the localization of RNF168 and TOP2α in S phase cells. Mouse embryonic fibroblasts (MEFs) were stained with the S phase marker 5-ethynyl-2′-deoxyuridine (EdU), anti-RNF168, anti-TOP2α and 4,6-diamidino-2-phenylindole (DAPI). We observed coexpression of RNF168 and TOP2α in S phase cells (EdU-positive). These studies also indicated the colocalization of RNF168 and TOP2α S phase foci (revised Fig. 1d and Supplementary Fig. 1). Collectively, these results validate the interaction between RNF168 and TOP2α.

### Important role for RNF168 in DNA decatenation

**Figure 2:**
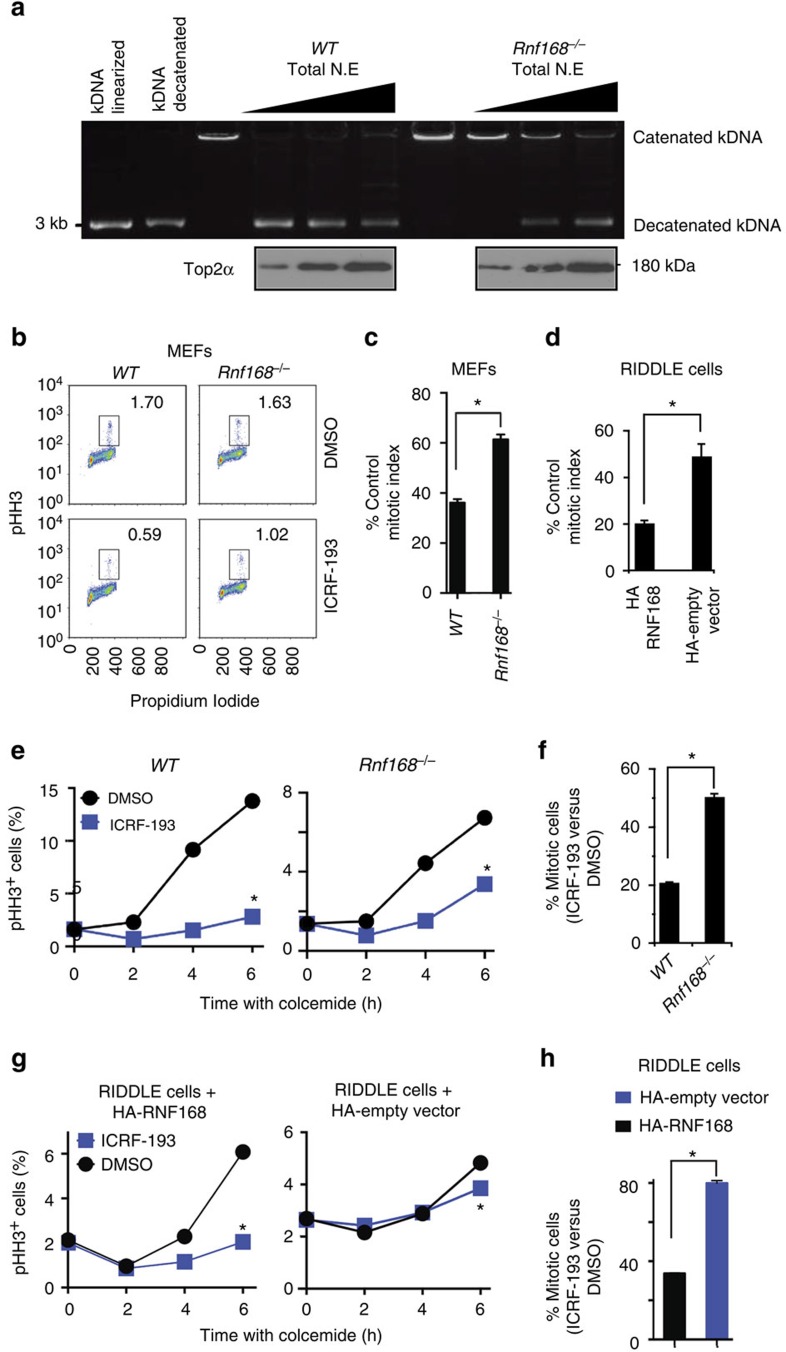
RNF168 stimulates DNA decatenation and is required for effective decatenation G2 checkpoint. (**a**) Representative agarose gel of *in vitro* kinetoplast DNA-based decatenation assays performed for 10 min with different amount of nuclear extracts from WT and *Rnf168*^*−/−*^ MEFs. Catenated and decatenated kDNA were separated by electrophoresis using 1% agarose gel. IB show Top2α's level in the total nuclear extracts used for this assay. (**b**) Representative data of the mitotic inhibition assay of decatenation G2 checkpoint in WT and *Rnf168*^*−/−*^ primary MEFs. Cells were treated with DMSO or ICRF-193 for 15 min and then incubated in culture media for an additional 2 h. The fraction of mitotic cells (pHH3^+^) was determined by flow cytometry. (**c**) Bar graphs represent the mean inhibition of mitotic index 2 h post ICRF-193 treatment of passage 1 primary MEFs (% pHH3^+^ cells post ICRF-193 treatment compared with DMSO-treated controls). *P<0.05. (**d**) Bar graphs represent the mean inhibition of mitotic index 2 h post ICRF-193 treatment of RIDDLE cells reconstituted with either HA-RNF168 or HA-empty vector as in **c**. *P<0.05. (**e**) Analysis of decatenation G2 checkpoint of *Rnf168*^*−/−*^ and WT 3T3 MEFs using the mitotic entry assay. Percentage of pHH3^+^ cells is shown at the indicated time post-treatment with colcemid in the presence of DMSO or ICRF-193. *P<0.05; *Rnf168*^*−/−*^ MEFs compared with WT MEFs 6 h post-ICRF-193 treatment. (**f**) Bar graphs represent the mean fraction of pHH3^+^ WT and *Rnf168*^*−/−*^ MEFs evading G2 arrest 6 h post-treatment with ICRF-193 compared with DMSO-treated cells as in **e**. *P<0.05. (**g**) Mitotic entry assay of decatenation G2 checkpoint in human RIDDLE cells reconstituted with HA-empty vector or HA-RNF168. Percentage of pHH3^+^ cells is shown at the indicated times post-treatment with colcemid with or without ICRF-193. *P<0.05, RIDDLE cells reconstituted with HA-empty vector compared with RIDDLE cells reconstituted with HA-RNF168 at 6 h post-ICRF-193 treatment. (**h**) Bar graphs represent the mean fraction of RIDDLE cells (reconstituted with HA-empty vector compared with those reconstituted with HA-RNF168) evading G2 arrest 6 h post ICRF-193 treatment compared with DMSO-treated controls as in **f**. *P<0.05. Three independent experiments in triplicates unless specified. Error bars in **c**,**d**,**f** and **h** represent mean±s.e.m.

TOP2α-mediated decatenation of newly replicated sister chromatids is required for chromosome segregation and the entry of cells into mitosis^1^,^2^. To further test whether RNF168 affects TOP2α function, we performed a standard *in vitro* kinetoplast DNA-based decatenation assay using total nuclear extracts from primary MEFs and thymocytes deficient for Rnf168, and compared their decatenation activities with their wild-type (*WT*) controls. *Rnf168*^*−/−*^ cells showed reduced decatenation activity compared with *WT* controls (Fig. 2a; Supplementary Fig. 2), thus supporting the importance of RNF168 in DNA decatenation.

TOP2α is also required for the decatenation G2 checkpoint^5^,^6^. This checkpoint is distinct from the DNA damage-induced G2 checkpoint and serves to delay entry of cells with insufficiently decatenated chromatids into mitosis. To further test the effects of RNF168 deficiency on TOP2α function, we carried out decatenation G2 checkpoint assays that rely on TOP2 inhibition by the bisdioxopiperazine ICRF-193. This catalytic inhibitor of TOP2 blocks decatenation of intertwined daughter chromatids in G2 phase, resulting in activation of the decatenation G2 checkpoint and delayed progression into mitosis^1^,^20^,^21^. While ICRF-193 does not generally produce DSBs, it has been reported to do so under certain conditions^22^,^23^. Therefore, we first tested whether ICRF-193 induces DSBs in *WT* and *Rnf168*^*−/−*^ MEFs under our treatment conditions. Using foci for Serine 139 phosphorylated H2a.x (γ-H2a.x) as markers DSBs (ref. 24), we observed that ICRF-193 did not generate DSBs in these cells (Supplementary Fig. 3a,b). Therefore, we used ICRF-193-based assays to measure the activation of decatenation G2 checkpoint under our experimental conditions. We first performed a mitotic inhibition assay to assess the activation of the decatenation G2 checkpoint^25^. Cells treated with ICRF-193 or dimethyl sulfoxide (DMSO) for 15 min were incubated in culture media for an additional 2 h and the frequency of mitotic cells (phospho-histone-H3 positive, denoted ‘pHH3^+^') was determined by flow cytometry. ICRF-193 treatment significantly reduced the frequency of mitotic cells in *WT* MEFs and HA-RNF168-reconstituted RIDDLE cells compared with *Rnf168*^*−/−*^ MEFs and HA-vector-reconstituted RIDDLE cells, respectively (Fig. 2b–d; Supplementary Fig. 4a). We next performed a mitotic entry assay^25^ to further examine the effects of RNF168 deficiency on the decatenation G2 checkpoint. Cells were treated for different durations (2–6 h) with either the microtubule depolymerization inhibitor colcemid alone, to prevent mitotic exit, or in combination with ICRF-193 to also block decatenation and delay entry into mitosis. The rate of mitotic entry of cells measured by pHH3 staining and flow cytometry showed higher mitotic rates for *Rnf168*^*−/−*^ MEFs and RIDDLE cells compared with *WT* MEFs and RNF168-reconstituted RIDDLE cells, respectively (Fig. 2e–h; Supplementary Fig. 4b,c). Taken together, RNF168 deficiency leads to defective DNA decatenation and impaired activation of the decatenation G2 checkpoint, thus supporting a model in which loss of RNF168 inhibits TOP2α activity.

### RNF168 deficiency increases the resistance to TOP2 drugs

**Figure 3:**
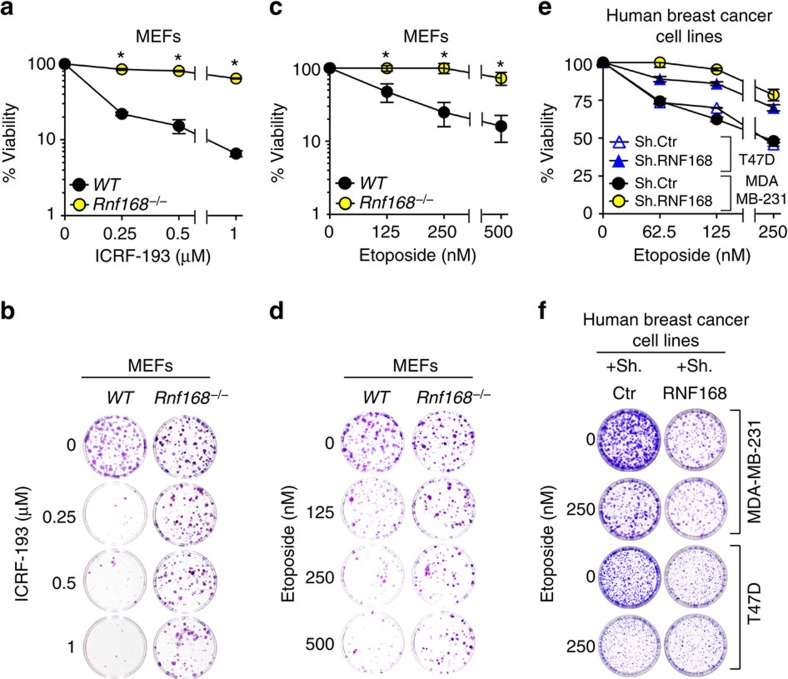
RNF168 mouse cells and human breast cancer cell lines to cytotoxic effects of TOP2 catalytic inhibitors and poisons. (**a**–**d**) Sensitivity of WT and *Rnf168*^*−/−*^ MEFs to ICRF-193 (**a**,**b**) or etoposide (**c**,**d**) was determined using clonogenic assays. (**e**,**f**) Sensitivity of the human breast cancer cell lines T47D and MDA-MB-231 to etoposide was determined using clonogenic assays. (**a**,**c**,**e**) Data are presented as the mean±s.e.m. (**a**,**c**, *n*>4; **e**, *n*=4). *P<0.05 for *Rnf168*^*−/−*^ MEFs compared with WT MEFs and T47D and MDA-MB-231 cells knocked down for RNF168 (sh.RNF168) compared with their respective controls (Sh.Ctr: ShRNA control). (**b**,**d**) Representative pictures of dishes showing surviving WT and *Rnf168*^*−/−*^ colonies post ICRF-193 (**b**) or etoposide (**d**) treatment. (**f**) Representative pictures of dishes showing surviving colonies of etoposide treated T47D and MDA-MB-231 cells.

To directly determine the effect of RNF168 deficiency on TOP2α function, we examined the cytotoxic effects of different concentrations of ICRF-193 on *Rnf168*^*−/−*^ MEFs and their *WT* controls by performing clonogenic assays. Camptothecin, a TOP1 inhibitor^26^, was used as a control for comparison. Our data indicated that while *Rnf168*^*−/−*^ MEFs displayed a similar sensitivity to camptothecin as compared with *WT* controls (Supplementary Fig. 5), they were more resistant to ICRF-193-induced cytotoxicity (Fig. 3a,b).

Etoposide, a TOP2 poison widely used for cancer therapy^21^,^27^, was also used to examine the effect of RNF168 deficiency on its cytotoxic effects. Analysis of clonogenic assays indicated that, similar to ICRF-193 treatment, *Rnf168*^*−/−*^ MEFs were highly resistant to etoposide-induced cytotoxicity (Fig. 3c,d). We next examined a panel of human breast cancer cell lines for their RNF168 expression levels and their response to etoposide. IB analysis indicated lower expression levels of RNF168 in the breast cancer cell lines MDA-MB-415 and MCF7 compared with SKBR3, T47D and MDA-MB-231 (Supplementary Fig. 6a). To determine whether RNF168 expression level correlates with the response to etoposide, we examined the cytotoxic effect of etoposide on breast cancer cell lines that have different levels of endogenous RNF168. SKBR3, T47D and MDA-MB-231 cancer cell lines, which highly express RNF168, were more sensitive to etoposide compared with the low RNF168-expressing MCF7 and MDA-MB-415 cancer cell lines (Supplementary Fig. 6b,c). To further elucidate the effect of RNF168 expression on the response of breast tumour cells to etoposide, we examined the effect of RNF168 knockdown on etoposide-induced cytotoxicity of T47D and MDA-MB-231 cell lines. Clonogenic assays using different doses of etoposide indicated that knockdown of RNF168 in T47D and MDA-MB-231 promotes their resistance to etoposide (Fig. 3e,f).

Collectively these data indicate that RNF168 deficiency promotes resistance to cytotoxicity induced by TOP2 catalytic inhibitors and poisons, further suggesting the importance of RNF168 for TOP2α function.

### RNF168 mediates polyubiquitylation of TOP2α

**Figure 4:**
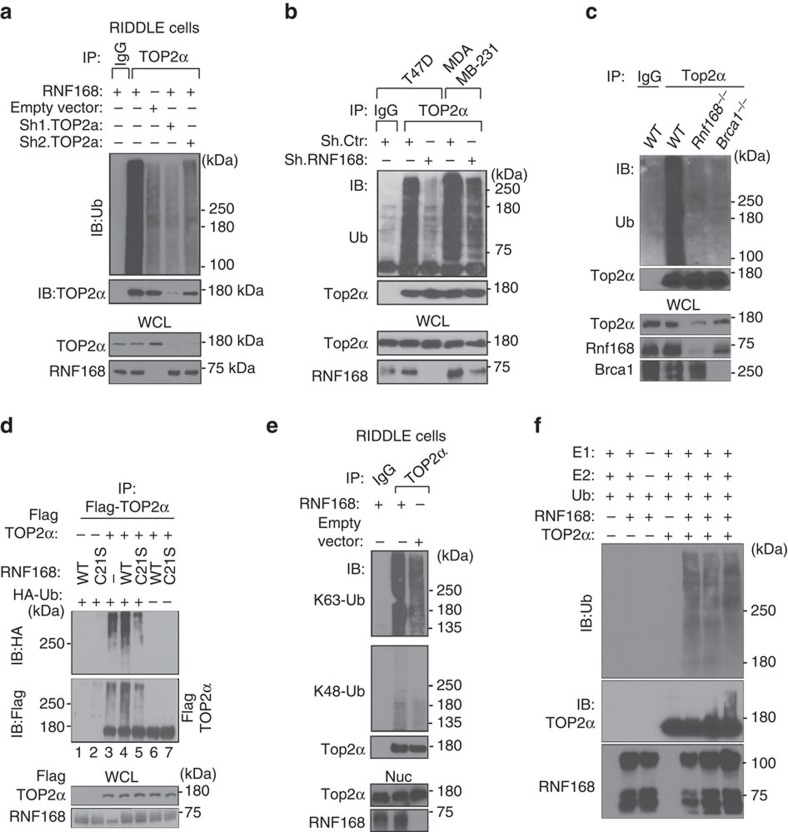
RNF168 mediates ubiquitylation of TOP2α. (**a**) RIDDLE cells reconstituted with HA-RNF168 or HA-empty vector, and control HA-RNF168-reconstituted RIDDLE cells with TOP2α knock down were lysed and WCL subjected to IP with anti-TOP2α or IgG (control) antibodies. Immunoprecipitates were blotted with the indicated antibodies to detect ubiquitylated TOP2α. (**b**) Human breast cancer cell lines T47D and MDA-MB-231 knocked down for RNF168 (Sh.RNF168) and their control expressing ShRNA control (sh.Ctr) were examined for their level of ubiquitylated TOP2α as in **a**. (**c**) *Rnf168*^*−/−*^, *Brca1*^*−/−*^ and WT MEFs were lysed and subjected to IP with anti-Top2α or IgG (control) antibodies. IPs from WCL were blotted with the indicated antibodies. (**d**) HEK293T cells were transfected with RNF168 (WT or mutant Rnf168-C21S), Flag-TOP2α and HA-Ub vectors as indicated. WCL were subjected to IP with anti-Flag, and IB analysis was performed using anti-HA antibody to detect ubiquitylated Flag-TOP2α. (**e**) Nuclear extracts from RIDDLE cells reconstituted with HA-RNF168 or HA-empty vector were subjected to IP with anti-TOP2α or IgG (control) antibodies. Immunoprecipitates were blotted with the indicated antibodies against K63- and K48-Ub linkages. (**f**) *In vitro* ubiquitylation of recombinant TOP2α in the presence of recombinant RNF168 (500 ng for lane 5, 1 μg for lanes 2, 3 and 6 and 2 μg for lane7), UBE1 (E1), UBE2E2 (E2) and Ub. Nuc, nuclear extract; WCL, whole-cell lysate.

Ubiquitylation plays important roles in regulating the function and turnover of TOP2α (ref. 2). As RNF168 interacts with TOP2α, we tested whether RNF168 could mediate ubiquitylation of TOP2α. Intracellular ubiquitylation assays indicated that endogenous TOP2α immunoprecipitated from RNF168-reconstituted RIDDLE cells, but not from the RIDDLE cells deficient for RNF168, were highly reactive to antibodies specific for ubiquitin (Ub; Fig. 4a). Knockdown of TOP2α in RNF168-reconstituted RIDDLE cells confirmed that RNF168 indeed polyubiquitylates endogenous TOP2α (Fig. 4a). Some of the TOP2α immunoprecipates from RNF168-expressing cells that were reactive to anti-Ub were smaller in size compared with the 170 KDa full-length TOP2α, suggesting that these smaller proteins might be the result of TOP2α degradation or TOP2α co-immunoprecipitating proteins.

We also performed *in vivo* ubiquitylation assays and examined the effect of RNF168 deficiency in human breast cancer cell lines on the level of TOP2α ubiquitylation. We observed that knockdown of RNF168 in the tumour cell lines T47D and MDA-MB-231 restrained ubiquitylation of their endogenous TOP2α (Fig. 4b). Consistent with these data, ubiquitylation levels of Top2α in *Rnf168*^*−/−*^ MEFs were greatly reduced compared with *WT* controls (Fig. 4c).

Given that the E3 ligases RNF8, RNF168 and BRCA1 promote DSB repair^11^, and that BRCA1 has been reported to ubiquitylate TOP2α (refs 9, 10), we were interested to see the effects of the deficiency of these E3 ligases on TOP2α ubiquitylation. As expected, *Brca1*^*−/−*^ MEFs displayed similarly reduced Top2α ubiquitylation compared with *Rnf168*^*−/−*^ MEFs (Fig. 4c). In contrast, RNF8, which plays important roles in the recruitment of RNF168 and BRCA1 to DSB flanking sites^11^,^28^, failed to interact with TOP2α, nor mediate its ubiquitylation (Supplementary Fig. 7a,b). Ubiquitylation of Top2α was also not affected in MEFs deficient for either Rnf8 or 53bp1, an important regulator of the DSB response^29^ (Supplementary Fig. 7c). Altogether, these data indicate that RNF168 and BRCA1, but not RNF8, are major E3 ligases that mediate TOP2α ubiquitylation.

To further examine RNF168-mediated ubiquitylation of TOP2α, we also performed intracellular ubiquitylation assays. We observed that TOP2α smears became stronger when both exogenous RNF168-WT and Ub were co-expressed with the full-length TOP2α in HEK293T cells (Fig. 4d, lane 4). However, ubiquitylation of TOP2α was reduced when TOP2α was co-expressed with the Ub ligase-dead mutant RNF168 (RNF168-C21S) compared with RNF168-WT (Fig. 4d, cf. lanes 4 and 5). These data verify the importance of the E3 ligase activity of RNF168 for the ubiquitylation of TOP2α.

Given that different Ub linkage types exist and can lead to distinct cellular outcomes^30^, and that K48 and K63-Ub-linkages are the predominant forms of polyubiquitylation in cells, we performed intracellular ubiquitylation assays to explore whether RNF168-ubiquitylated TOP2α contains these Ub-linkages. Using Ub in which all lysines (K) except K48- or K63 were mutated to arginines, we observed that RNF168 preferentially catalyses K63-linked ubiquitylation of TOP2α (Supplementary Fig. 7d). Consistent with these data, immunoprecipitated TOP2α from RNF168-reconstituted RIDDLE cells showed increased reactivity to antibodies against K63-Ub but not K48-Ub (Fig. 4e). These data support that RNF168 polyubiquitylation of TOP2α is mediated predominantly by K63-linkages.

Similar to ubiquitylation, SUMOylation also regulates TOP2α functions^2^. In *Xenopus*, TOP2α SUMOylation on K660 (K662 in *Homo sapiens*) inhibits its decatenation activity^2^,^31^,^32^. Substitution of K660 to arginine (R) in TOP2α of *Xenopus* eliminates its SUMOylation-dependent inhibition^32^. Given that Ub and SUMO modifications have been shown to compete for the same K residues on certain substrates^33^, we sought to examine whether substitution K662R of TOP2α affects its ubiquitylation by RNF168. *In vitro* ubiquitylation analysis indicated reduced level of RNF168 ubiquitylation of TOP2α-K662R compared with TOP2α-WT (Supplementary Fig. 7e). These data suggest that K662 of TOP2α is a target for RNF168 ubiquitylation. However, our observation that RNF168 ubiquitylation of TOP2α is not fully abrogated in the presence of K662R substitution, suggests that additional TOP2α residues are also ubiquitylated by RNF168.

To further examine whether TOP2α is a direct substrate for RNF168, we performed *in vitro* ubiquitylation assays using recombinant TOP2α, recombinant RNF168, E1 and E2 (Fig. 4f). These *in vitro* ubiquitylation assays indicated the ability of recombinant RNF168 to ubiquitylate recombinant TOP2α, thus suggesting that RNF168 is a direct E3 ligase for TOP2α ubiquitylation.

### RNF168 E3 ligase activity stimulates TOP2α function

**Figure 5:**
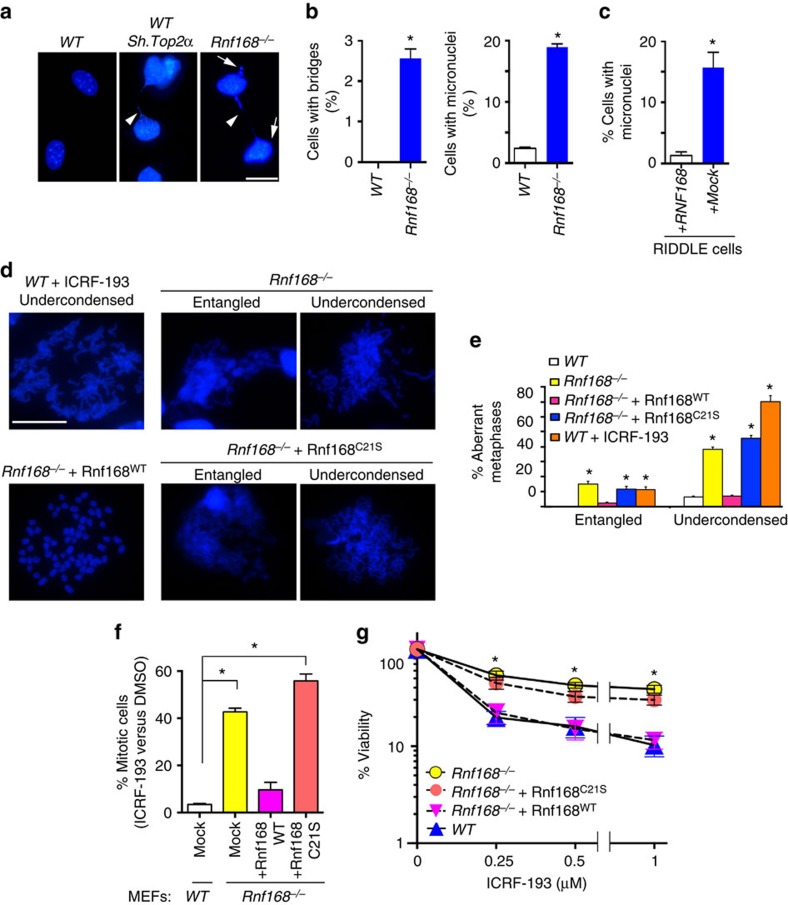
Deficiency of the E3 ligase RNF168 impairs chromosome segregation and promotes chromosome entanglement and under-condensation. (**a**) WT and *Rnf168*^*−/−*^ MEFs, and Top2α knockdown control MEFs were fixed and stained with DAPI. Representative cells with defective chromosome segregation, as indicated by chromosome bridges (arrow head) and micronuclei (arrow) are shown. (**b**) Histograms show quantification of cells with chromosome bridges or micronuclei (mean±s.e.m., *n*=3). *P<0.05. (**c**) Histograms show the fraction of RIDDLE cells reconstituted with HA-RNF168 or HA-empty vector that display micronuclei (mean±s.e.m., *n*=3). *P<0.05. (**d**; upper panels) Representative metaphase spreads showing undercondensed chromosomes in WT MEFs 24 h post ICRF-193 treatment (positive control), and in *Rnf168*^*−/−*^ DMSO-treated MEFs. (**d**, lower panels) Representative metaphase spreads of DMSO-treated *Rnf168*^*−/−*^ MEFs complemented with RNF168-WT (WT) or the E3 ligase deficient RNF168-C21S (C21S). (**e**) Histograms show quantification of abnormal metaphase spreads with entangled or undercondensed chromosomes from the indicated cells (mean±s.e.m., *n*=3). *P<0.05 compared with WT MEFs. (**f**) Histograms present the mean fraction of MEFs evading ICRF-193-induced G2 arrest as compared with DMSO-treated controls (mean±s.e.m., *n*=3). Data are shown for WT MEFs, mock infected *Rnf168*^*−/−*^ MEFs and *Rnf168*^*−/−*^ MEFs complemented with RNF168-WT or RNF168-C21S. 3T3 MEFs were used for these experiments. Data shown are for 6 h post-treatment with colcemid±4 μM ICRF-193. *P<0.05 compared with WT MEFs. (**g**) Clonogenic assay was used to determine sensitivity to ICRF-193 of mock infected WT and *Rnf168*^*−/−*^ MEFs, as well as *Rnf168*^*−/−*^ MEFs complemented with RNF168 (WT or C21S mutant). Data are presented as the mean±s.e.m. (*n*=4). *P<0.05 compared with WT MEFs. Scale bar, 20 μm.

Based on the interaction of RNF168 and TOP2α, and our observation that TOP2α ubiquitylation by RNF168 does not increase its turnover, we hypothesized that RNF168 may regulate TOP2α. Given that TOP2α inactivation results in abnormal chromosome segregation^1^,^2^, we first examined whether RNF168 deficiency may also affect chromosome segregation. Defects in chromosome segregation can lead to increased frequency of lagging chromosomes that promote the formation of micronuclei as well as formation of chromosome bridges^5^,^34^. Interestingly, in contrast to WT MEFs, but similar to cells knocked down for Top2α, *Rnf168*^*−/−*^ MEFs readily displayed increased frequency of abnormal interphase nuclei with chromosome bridges (Fig. 5a,b and Supplementary Fig. 8). Similar to Top2α*-*knocked down cells, *Rnf168*^*−/−*^ MEFs also displayed a significantly increased frequency of micronuclei (Fig. 5a,b and Supplementary Fig. 8). Human cells from the RIDDLE patient 15-9BI also showed increased frequency of micronuclei compared with their controls complemented with RNF168-WT (Fig. 5c).

TOP2α is also essential for the condensation of mitotic chromosomes; and cells knocked down for TOP2α or treated with its catalytic inhibitor ICRF-193 display impaired chromosome condensation and increased frequency of entangled chromosomes^2^,^3^,^5^,^35^. Therefore, we further tested our hypothesis that RNF168 regulates TOP2α function by examining the effects of RNF168 deficiency on chromosome condensation. Metaphase spreads of WT and *Rnf168*^*−/−*^ MEFs as well as Top2α*-*knocked down MEFs were examined for their frequency of undercondensed and entangled chromosomes. As expected, the frequency of these aberrant chromosomes was increased in WT MEFs treated with ICRF-193 or following Top2α knockdown (Fig. 5d and Supplementary Fig. 8). Under untreated conditions, and in contrast to WT cells, *Rnf168*^*−/−*^ MEFs already displayed elevated frequencies of entangled and undercondensed chromosomes (Fig. 5d,e). The requirement of RNF168 for normal segregation and condensation of mitotic chromosomes, and decreased ability of etoposide to generate DSBs in *Rnf168*^*−/−*^ MEFs compared with WT controls (Supplementary Figs 9 and 10), support our hypothesized role for RNF168 in regulating TOP2α function.

Given that the Ub ligase-dead mutant RNF168-C21S was defective in mediating TOP2α ubiquitylation (Fig. 4d), we examined the importance of the E3 ligase function of RNF168 for TOP2α activity using *Rnf168*^*−/−*^ MEFs reconstituted with empty vector (mock), Rnf168-WT or the Ub ligase-dead Rnf168-C21S mutant^36^. We first examined the frequency of undercondensed and entangled chromosomes in metaphase spreads of these cells. While complementation of *Rnf168*^*−/−*^ MEFs with Rnf168-WT reduced their frequency of undercondensed and entangled chromosomes, their complementation with the Rnf168-C21S mutant failed to rescue these defects (Fig. 5d,e).

TOP2α function is required for the decatenation G2 checkpoint^5^,^6^; therefore we examined the effect of RNF168 E3 ligase activity on this checkpoint using the mitotic entry assay^25^. Reconstitution of *Rnf168*^*−/−*^ MEFs with Rnf168-WT reduced their fraction of mitotic cells to a level comparable to WT MEFs (Fig. 5f; Supplementary Fig. 11). This rescue was however entirely dependent on Rnf168's E3 ligase activity since *Rnf168*^*−/−*^ MEFs complemented with Rnf168-C21S mutant retained their defective decatenation G2 checkpoint (Fig. 5f; Supplementary Fig. 11). This finding prompted us to perform clonogenic assays and examine the effect of the loss of Rnf168 Ub ligase function on the response to ICRF-193-induced cytotoxicity. While complementation of *Rnf168*^*−/−*^ MEFs with Rnf168-WT restored their sensitivity to ICRF-193 to a level similar to WT controls, complementation of these cells with Rnf168-C21S mutant failed to suppress their resistance to ICRF-193 cytotoxicity (Fig. 5g). The inability of the E3 ligase-dead Rnf168 to rescue the defects of *Rnf168*^*−/−*^ MEFs, including impaired segregation and condensation of chromosomes, the decatenation G2 checkpoint and the resistance to the TOP2 inhibitor ICRF-193; all support the importance of this E3 ligase for the stimulation of TOP2α activity.

We also performed the standard *in vitro* kinetoplast DNA-based decatenation assay and examined decatenation activity of recombinant TOP2α that was pre-ubiquitylated *in vitro* by recombinant RNF168 (Fig. 4f). We observed that *in vitro* ubiquitylation of recombinant TOP2α by RNF168 increases its decatenation activity (Supplementary Fig. 12). Collectively, these results support the importance of the E3 ligase function of RNF168 in the stimulation of TOP2α activity.

### RNF168 ubiquitylates TOP2α to activate its chromatin-binding

**Figure 6:**
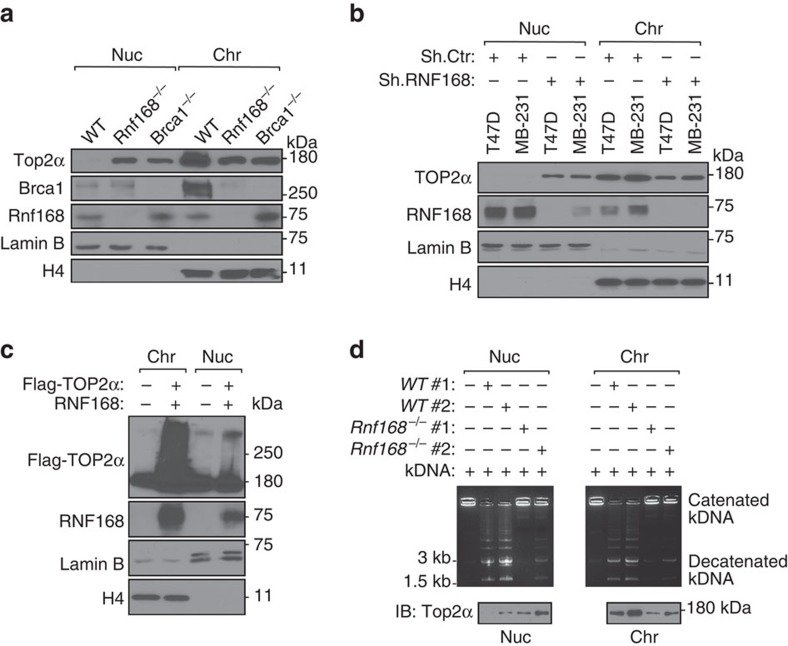
The E3 ligase RNF168 promotes TOP2α-chromatin association and stimulates decatenation activity in nuclear and chromatin fractions. (**a**) Nuclear (Nuc) and chromatin (Chr) fractions prepared from WT, *Rnf168*^*−/−*^ and *Brca1*^*−/−*^ MEFs were analysed by IB for chromatin occupancy of Top2α. Additional IBs were performed with the indicated antibodies as controls. H4, histone H4. (**b**) Nuclear and chromatin fractions were prepared from the human breast cancer cell lines T47D and MDA-MB-231 knocked down for RNF168 (Sh.RNF168) and their controls (Sh.Ctr) and analysed by IB for the chromatin occupancy of TOP2α as in **a**. (**c**) HEK293T were transfected with Flag-TOP2α along with RNF168 (+) or empty vector (−) and their nuclear and chromatin fractions were prepared and examined by IB using the indicated antibodies. (**d**) A representative agarose gel showing decatenation activity of soluble nuclear and chromatin extracts from 2 WT and 2 *Rnf168*^*−/−*^ MEFs. *In vitro* kinetoplast DNA-based decatenation assay was performed for 20 min with different amounts of nuclear and chromatin extracts, and catenated and decatenated kDNA were separated by electrophoresis. IB using anti-Top2α was performed to show the level of Top2α present in each sample.

TOP2α binding to chromatin is a necessary step for its biological function^1^,^2^. We therefore examined whether impaired ubiquitylation of TOP2α due to the absence of RNF168 affects its chromatin binding. IB analysis of WT MEFs indicated enrichment of Top2α in the chromatin fraction compared with the nuclear fraction (Fig. 6a). However, *Rnf168*^*−/−*^ MEFs displayed decreased levels of Top2α in their chromatin fraction, whereas their nuclear fractions displayed higher levels of Top2α compared with WT controls (Fig. 6a).

To confirm these findings in human cells, we performed similar fractionation experiments of breast cancer cell lines T47D and MDA-MB-231 and their RNF168-knockdown counterparts. We observed that in contrast to controls, but similar to *Rnf168*^*−/−*^ cells, RNF168-knockdown T47D and MDA-MB-231 cells displayed enrichment of TOP2α nuclear fractions (Fig. 6b).

Our data showed that RNF168 ubiquitylates TOP2α, and defects of this ubiquitylation affect TOP2 levels in the nuclear and chromatin fractions. Therefore, we next examined the effects of RNF168 overexpression on the ubiquitylation level of TOP2α in nuclear and chromatin fractions. We observed that co-overexpression of RNF168 and Flag-TOP2α in HEK293T cells enriches ubiquitylated TOP2α in both fractions; although this enrichment was more prominent in the chromatin fraction (Fig. 6c).

TOP2α from both nuclear and chromatin fractions have been previously reported to possess decatenation activity^9^,^37^; and therefore, we performed *in vitro* kinetoplast DNA-based decatenation assays and examined the effects of Rnf168 deficiency on decatenation activities in the chromatin and nuclear fractions. Despite the elevated level of Top2α in the nuclear fractions of *Rnf168*^*−/−*^ MEFs, this fraction exhibited reduced decatenation activity compared with the nuclear fraction of WT MEFs (Fig. 6d). Furthermore, while Rnf168 deficiency impaired Top2α-chromatin association (Fig. 6a,b), *Rnf168*^*−/−*^ MEFs also exibited decreased decatenation activity in the chromatin fraction (Fig. 6d). We propose that RNF168 ubiquitylates TOP2α in the nuclear and chromatin fractions, and promotes its decatenation activity and chromatin association. Taken together these data highlight the importance of RNF168 for the regulation TOP2α function.

### USP10 deubiquitylates TOP2α and restrains its chromatin binding

**Figure 7:**
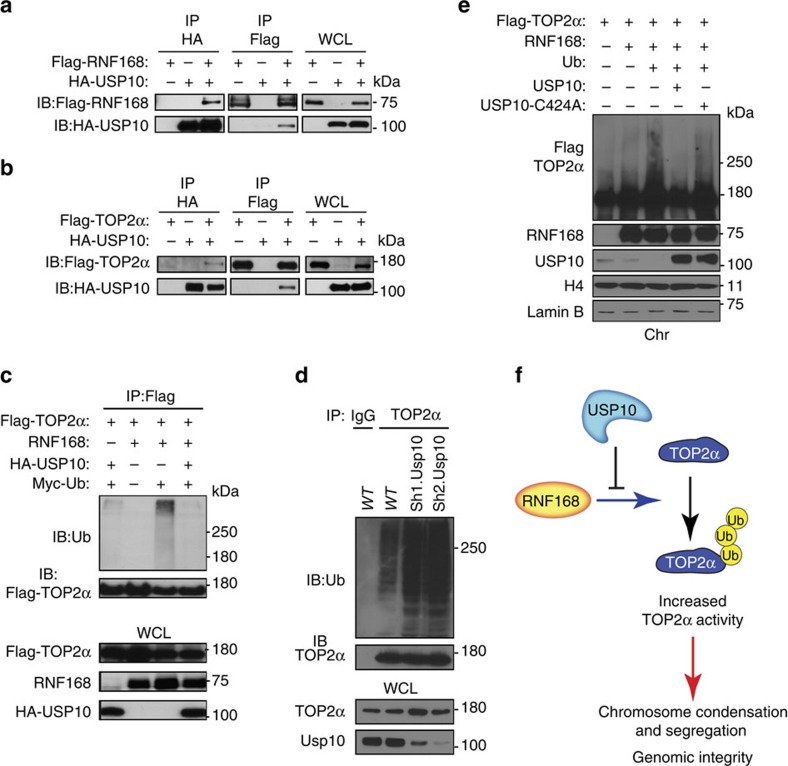
USP10 interacts with TOP2α and RNF168 to antagonize RNF168 ubiquitylation of TOP2α and its chromatin binding. (**a**,**b**) HEK293T cells were transfected with Flag-RNF168 and HA-USP10 vectors (**a**) or Flag-TOP2α and HA-USP10 vectors (**b**) as indicated. Cells were lysed and IP was performed using anti-Flag and anti-HA antibodies. The resulting precipitates were subjected to IB analysis with the indicated antibodies. WCL, whole-cell lysate. (**c**) HEK293T cells were transfected with Flag-TOP2α, RNF168, HA-USP10 and Myc-Ub vectors, as indicated. IP using anti-Flag and WCL was subjected to anti-Ub IB analysis to detect TOP2α ubiquitylation. (**d**) *WT* MEFs with knockdown of Usp10 (Sh1 and Sh2) and *WT* controls were examined for the level of Top2α ubiquitylation. Top2α was immunoprecipitated from whole-cell extracts and examined by IB for its ubiquitylation level using anti-Ub. IP using IgG was used as a control. The indicated antibodies were used for IB. (**e**) HEK293T cells were transfected with Flag-TOP2α with or without RNF168, HA-Ub, USP10 and USP10-C424A as indicated. TOP2α chromatin occupancy in these cells was examined by IB using anti-Flag antibodies and chromatin fractions (Chr). IB analysis of the chromatin fractions is also shown for the indicated antibodies. (**f**) A simplified model of RNF168-mediated regulation of TOP2α ubiquitylation and decatenation function.

Although ubiquitylation is central to the regulation of TOP2α function^2^, deubiquitylases that reverse this ubiquitylation have not yet been identified. Given that our mass spectrometry analysis identified USP10 as a possible RNF168-interacting partner (Supplementary Table 1), we hypothesized that USP10 and RNF168 forms a complex with TOP2α to co-regulate its ubiquitylation level. We tested this hypothesis by coexpressing HA-USP10 with Flag-RNF168 or Flag-TOP2α in HEK293T cells. Indeed, IP/IB analysis confirmed interaction of USP10 with RNF168 (Fig. 7a). Furthermore, we also observed interaction of USP10 with TOP2α (Fig. 7b). Examination of synchronized MEFs, in response to serum starvation, aphidicolin or colcemid treatments, indicated no changes of USP10-TOP2α interaction (Supplementary Fig. 13a). However, similar to RNF168, USP10 interaction with TOP2α was reduced in response to radiation (Supplementary Fig. 13b).

Clonogenic assays were performed to examine the sensitivity to etoposide and ICRF-193 of Usp10 knockdown WT MEFs and their controls. These studies indicated no change of sensitivity to TOP2 drugs in Usp10-deficient MEFs (Supplementary Fig. 13c). We next examined the effects of USP10 on RNF168-mediated ubiquitylation of TOP2α. Intracellular ubiquitylation assays using HEK293T cells transfected with RNF168, Flag-TOP2α, HA-USP10 and Myc-Ub indicated that USP10 antagonizes RNF168 ubiquitylation of TOP2α (Fig. 7c, cf. lanes 3 and 4). Furthermore, we examined the effect of Usp10-deficiency on the levels of Top2α ubiquitylation. MEFs knocked down for Usp10 displayed increased Top2α ubiquitylation compared with WT controls (Fig. 7d). These data indicate that the deubiquitylase USP10 opposes RNF168-mediated ubiquitylation of TOP2α.

We also examined whether USP10 affects TOP2α's binding to chromatin. To this end, we transfected HEK293T cells with Flag-TOP2α alone or with various combinations of RNF168, HA-Ub, HA-USP10-WT or the catalytically inactive mutant USP10-C424A (ref. 38) and performed IB analysis on chromatin-enriched fractions of these cells. The fraction of TOP2α bound to chromatin was increased in cells co-transfected with TOP2α and RNF168, and this fraction was further enriched by the additional coexpression of Ub (Fig. 7e, cf. lane 1 versus lanes 2 and 3). However, additional overexpression of USP10-WT in these cells significantly reduced the fraction of chromatin-bound TOP2α (Fig. 7e, cf. lane 3 versus lane 4). In contrast to USP10-WT, overexpression of USP10-C424A was inefficient in reducing the level of chromatin-bound TOP2α (Fig. 7e, cf. lane 4 versus lane 5).

Collectively these data reveal that RNF168 and USP10 exert important, but opposing, effects on the regulation of TOP2α's ubiquitylation status and association with chromatin.

## Discussion

TOP2α is essential for DNA decatenation and chromosome segregation, and is an important target for cancer therapy^1^,^2^,^39^. The physiological importance of TOP2α is highlighted by the early embryonic lethality associated with its deficiency^40^. In accordance with its important functions, a number of post-translational modifications have been reported to regulate TOP2α's turnover and activity^1^,^2^,^3^. For instance, TOP2α ubiquitylation by BMI1/RING1A or APC/C-Cdh1 stimulates its turnover^2^,^7^,^8^. TOP2α is also ubiquitylated by BRCA1, although this ubiquitylation was reported to stimulate both its activity and proteasomal degradation^9^,^10^. Given the biological importance of TOP2α, its inhibition in cancer therapy, and the role ubiquitylation plays in regulating its turnover and activity, it is of importance to identify other E3 ligases that may ubiquitylate TOP2α. Furthermore, there is also a need to identify mechanisms that regulate deubiquitylation of TOP2α.

In the present study, we report polyubiquitylation of TOP2α by RNF168, an E3 ligase known for its role in DSB signalling and being mutated in the human RIDDLE syndrome. *In vitro* ubiquitylation studies confirmed that TOP2α is a direct substrate for RNF168. We also provide data to support that RNF168 ubiquitylation of TOP2α is mediated mainly by K63-Ub-specific linkages that are rarely associated with proteolysis^30^. Consistent with this finding, we observed that RNF168 ubiquitylation of TOP2α does not promote its degradation, but it rather stimulates its function. Finally, we provide evidence that RNF168-mediated ubiquitylation of TOP2α stimulates its decatenation activity.

Previous studies reported decatenation activity of TOP2α from nuclear and chromatin fractions^9^,^37^. Consistent with a role of RNF168 in the regulation of both nuclear and chromatin-bound TOP2α, overexpression of this E3 ligase increased ubiquitylation levels of TOP2α in both fractions, although the chromatin-bound TOP2α showed more pronounced ubiquitylation.

Consistent with the importance of RNF168 ubiquitylation of TOP2α for stimulating its function, deficiency of RNF168 results in defective activation of the decatenation G2 checkpoint, elevated frequency of chromosome aberrations (undercondensed and/or entangled), and impaired chromosome segregation (bridges and micronuclei). The E3 ligase function of RNF168 is required for its regulation of TOP2α function. Our study indicate that in contrast to RNF168-WT, its E3 ligase inactive form fails to rescue defective TOP2α function in cells deficient for RNF168. Collectively these data reveal a novel and important role for RNF168 in mediating TOP2α ubiquitylation and the activation of its decatenation function.

BRCA1 is a tumour suppressor with a major function in DSB signalling/repair^11^,^41^. Consistent with previous reports^9^,^10^, we observed that BRCA1 deficiency impairs TOP2α ubiquitylation level. TOP2α in the nuclear fraction of BRCA1-deficient cells has been shown to have defective decatenation activity, suggesting that BRCA1 stimulates TOP2α decatenation activity^9^. Similar to BRCA1-deficient cells, Top2α from the nuclear fraction of *Rnf168*^*−/−*^ cells also displayed defective decatenation activity. This defect was readily detected despite increased level of Top2α in the nuclear fraction of Rnf168 deficient cells. Furthermore, Rnf168 stimulates Top2α-chromatin binding and its loss reduces decatenation activity of Top2α in the chromatin fraction.

Deubiquitylases play major roles in several cellular processes and signalling pathways, and their defects have been associated with a number of diseases including cancer^42^. Deubiquitylases and E3 ligases exert opposing effects on the ubiquitylation levels of their substrates and, as such are critical for the regulation of ubiquitylation-dependent processes^43^. Despite the importance of TOP2α function and the role ubiquitylation plays in the regulation of its activity and turnover^1^,^2^, mechanisms that deubiquitylate TOP2α, and the deubiquitylases involved in this processes, remain elusive. USP10 is a deubiquitylase important for the deubiquitylation and regulation of turnover and function of a number of substrates including p53, H2A.Z, SIRT6, AMPK, TRAF6 and MSH2 (refs 38, 42, 44, 45, 46, 47, 48). Interestingly, in this study, we also identified USP10 as an interacting partner for both TOP2α and RNF168. Furthermore, we discovered that USP10 antagonizes RNF168-mediated ubiquitylation of TOP2α and decreases TOP2α binding to chromatin, suggesting a role for this deubiquitylase in opposing RNF168-mediated effects on TOP2α (Fig. 7f).

TOP2 poisons (for example, etoposide) are chemotherapeutic agents extensively used to treat a broad spectrum of human cancers^21^,^27^. However, resistance to these drugs is common and significantly limits their potential therapeutic benefit. Several mechanisms have been ascribed to drive tumour resistance to TOP2 poisons, including down-regulation of TOP2α expression^27^,^49^. In this study, we observed that RNF168 deficiency promotes the resistance to cytotoxicity induced by the TOP2 poison etoposide and its catalytic inhibitor ICRF-193. Notably, knock down of RNF168 in human breast cancer cell lines T47D and MDA-MB-231 promoted their resistance to etoposide-induced cytotoxicity. These findings suggest that RNF168 defects (mutations or loss of expression) may contribute to the resistance of cancer patients to TOP2 poisons.

Similarities exist between BRCA1 (ref. 9) and RNF168 in mediating ubiquitylation of TOP2α and stimulation of its decatenation activity. However, in contrast to RNF168, BRCA1 deficiency increases cytotoxicity to TOP2α catalytic inhibitors and poisons^50^,^51^. The intriguing sensitivity of BRCA1 mutant cells and tumors suggests additional functions of BRCA1 compared with RNF168. Indeed, a recent study indicated the requirement of BRCA1, together with the Mre11-Rad50-Nbs1 complex and CtIP, for the removal of etoposide-induced TOP2 protein-DNA adducts and the subsequent resection of etoposide-generated DSBs (ref. 52). Defects of these novel BRCA1 functions would sensitize cells to TOP2 catalytic inhibitors and poisons^53^. In addition, the critical roles of BRCA1 in the homologous recombination (HR) pathway of DSB repair and in the CtIP-dependent processing of etoposide-induced DSBs (ref. 52), support the importance of HR in the repair of etoposide-induced DSBs. Interestingly, in contrast to BRCA1, RNF168 deficiency only mildly affects HR-mediated DSB repair^36^. Therefore, the difference in the requirement for BRCA1 and RNF168 for HR-mediated repair could also contribute to the different response of BRCA1 and RNF168 deficient cells to TOP2 catalytic inhibitors and poisons.

In this study, we also report that in addition to RNF168, TOP2α also interacts with USP10. We show that USP10 antagonizes RNF168 ubiquitylation of TOP2α and restrains RNF168 stimulation of TOP2α binding to chromatin. USP10-deficient cells displayed increased ubiquitylation level of TOP2α compared with WT controls, confirming that USP10 is a deubiquitylating enzyme for TOP2α. Although it is conceivable that deregulation of this deubiquitylase could also contribute to the resistance of tumors to TOP2 drugs, given the growing number of its substrates and their involvement in different cellular processes^38^,^42^,^44^,^45^,^46^,^47^,^48^, this remains to be demonstrated. Further studies are also required to determine whether loss of RNF168 expression or its inactivation correlate with resistance to anti-cancer chemotherapeutics that target TOP2 in cancer patients.

In summary, our results reveal the importance of the E3 ligase RNF168 for mediating the TOP2α ubiquitylation and the stimulation of its decatenation activity. We also identified USP10 as an interacting partner for RNF168 and TOP2α, and provide evidence that USP10 deubiquitylates TOP2α and negatively regulates its chromatin binding (Fig. 7f). These novel functional interactions between RNF168, TOP2α and USP10 are likely to contribute to the maintenance of chromosomal integrity. In addition, our data suggest that these functional interactions between RNF168 and TOP2α may play important roles in the response and resistance of tumors to anti-cancer drugs that target TOP2.

## Methods

### Mass spectrometry analysis

Mock or Flag-RNF168 expressing HEK293 cells were used for affinity purification. Samples were resolved in a gel that was subsequently stained with silver staining for visualization. The entire protein containing gel chunk was split into two and processed for mass spectrometry as previously described^54^. Samples were resuspended in 0.1% (MS grade) formic acid (Sigma). Analytical columns (75 μm inner diameter) and pre-columns (100 μm) were prepared in-house from silica capillary tubing (InnovaQuartz, Phoenix, AZ), and packed with 5 μm 300 Å C18-coated silica particles (Michrom). Analytical columns were fitted with metal emitters (Thermo Proxeon) using zero dead volume connections. Peptides were subjected to nanoflow liquid chromatography-electrospray ionization-tandem mass spectrometry (nLC-ESI-MS/MS) , using a 90 min reversed phase (10–40% acetonitrile and 0.1% formic acid) buffer gradient running at 250 nl min^−1^ on a Proxeon EASY-nLC pump in-line with a hybrid linear quadrupole ion trap Orbitrap mass spectrometer (LTQ Orbitrap, Thermo Fisher Scientific). A parent ion scan was performed in the Orbitrap, using a resolving power of 60,000. Simultaneously, up to the four most intense peaks were selected for MS/MS (minimum ion count of 500 for activation) using standard collision-induced dissociation (CID) fragmentation. Fragment ions were detected in the LTQ. Dynamic exclusion was activated such that MS/MS of the same *m*/*z* (within a 0.1 Da window, exclusion list size 500) detected three times within 45 s were excluded from analysis for 60 s. Thermo RAW files were converted to mzXML (Proteowizard^55^), and searched with the iProphet^56^ pipeline in Prohits^57^ and Comet^58^ search algorithms, using a parent mass window of 20 p.p.m. and 0.4 Da fragment mass window, against a database containing human refseq v57 forward and reversed protein sequences. Up to two missed cleavages were allowed, and oxidation of methionine, deamidation of glutamine and asparagine were set as variable modifications. Polypeptides with a Trans-Proteomic Pipeline (TPP) probability of 1.00 (and identified with 4 or more unique peptides) are shown in Supplementary Table 1.

### Cells

MEFs were generated according to standard procedures using mice mutant for RNF168 (ref. 19), RNF8 (ref. 59), BRCA1 (ref. 60) and WT littermates. All animal experiments were done in compliance with the Ontario Cancer Institute animal care committee guidelines. *Rnf168*^*−/−*^ MEFs reconstituted with RNF168-WT or the mutant RNF168-C21S as well as RIDDLE 15-9BI cells and their controls complemented with RNF168-WT were previously described^17^,^36^. MEFs, HEK293T, MDA-MB-231, MDA-MB-415, SKBR3, MCF7 and RIDDLE cells were cultured in Dulbecco's modified Eagle's medium while T47D were cultured in Roswell Park Memorial Institute medium (RPMI; GIBCO) supplemented with 10% fetal bovine serum (Wissent). Cells were transduced with the following lentivirus-PLKO-puro constructs: ShRNA1-TOP2α (TRCN0000049281: 5′-CCCAACTTTGATGTGCGTGAA-3′), ShRNA2-TOP2α (TRCN0000291060: 5′-GCTCCAAATCAATATGTGATT-3′), ShRNA1-RNF168 (TRCN0000034137: 5′-GCAGTCAGTTAATAGAAGAAA-3′), ShRNA2-RNF168 (5′-GAAGAGTCGTGCCTACTGATT-3′), ShRNA1-Usp10 (TRCN0000288539: 5′-CGCAGAGGAGTATCTAGGTTT-3′) and ShRNA2-Usp10 (TRCN0000307627: 5′-CCAAACGGTCAAGGTTATTAA-3′).

### Constructs and mutagenesis

Full-length mouse *Rnf168* was amplified by PCR using complementary DNA from *WT* MEFs and subcloned in p3xFLAG-CMV10 (Sigma-Aldrich), pcDNA3-HA (Invitrogen) and pBabe-puro (Addgene; ID 1764). FLAG-TOP2α was kindly provided by Dr. Sullivan (H. Lee Moffitt Cancer Center and Research Institute). HA-RNF8 was kindly provided by Dr. Durocher (Samuel Lunenfeld Research Institute). Site-specific mutagenesis was performed using QuickChange kit (Stratagene).

### Retroviral and lentiviral infections

Ecotropic retroviruses were generated by transient transfection of Phoenix cells^36^. For lentviruses, HEK293T cells were co-transfected by calcium phosphate precipitation with pLKO constructs containing specific ShRNA or ShRNA control together with psPAX-2 and pMDG1.vsvg. 24 h later, the media was replaced with fresh complete growth medium and cells were incubated at 37 °C for an additional 24–48 h before harvesting the virus. Cells were infected with retrovirus or lentivirus supernatants containing polybrene (Sigma-Aldrich) and were subjected to puromycin selection (10 μg/ml).

### Clonogenic assay

3T3 MEFs (200 cells) and breast cancer cell lines (300 cells) were seeded on 6 cm dishes, treated with DMSO, ICRF-193, etoposide or camptothecin for 24 h and cultured for an additional 11 days. Colonies were stained with crystal violet and counted.

### Pull-down analysis

HEK293T cells were harvested 48 h post-transfection, washed twice with ice-cold PBS and centrifuged at 1,500 r.p.m. for 5 min at 4 °C. Cell pellets were resuspended in lysis buffer (50 mM Tris-HCl (pH 7.5), 150 mM NaCl, 0.5% Triton-X100 and protease inhibitor cocktail tablets) and incubated on ice for 30 min. Cell debris were removed by centrifugation at 13,200 r.p.m. for 30 min at 4 °C. Anti-Flag conjugated agarose beads (M2, Sigma-Aldrich) were added to the cleared lysate and the mixture was rotated for 16 h at 4 °C. Anti-Flag (M2) agarose beads were collected and washed five times with the lysis buffer and one time with detergent-free lysis buffer.

### Chromatin association assay

For chromatin fractionation, 3 × 10^7^ cells were washed with PBS and resuspended in 200 μl of buffer A (10 mM HEPES (pH 7.9), 50 mM NaCl, 0.5 M sucrose, 0.1 mM EDTA, 1 mM dithiothreitol, 1% NP40 and protease inhibitor mixture (Roche Molecular Biochemicals)). The cells were incubated for 5 min on ice and subsequently nuclei were collected in the pellet (P1) by low-speed centrifugation (3,000 r.p.m., 10 min, 4 °C). The pellet was washed once with buffer A then lysed in 200 μl of buffer B (10 mM HEPES (pH 7.9), 150 mM NaCl, 0.1 mM EDTA, 1 mM dithiothreito, 1% NP40 and protease inhibitor mixture), followed by 10-min incubation on ice. Soluble nuclear proteins (S2) were separated from chromatin by centrifugation (5,000 r.p.m., 5 min). The pellet (P2) was solubilized using sonication in buffer C (10 mM HEPES (pH 7.9), 250 mM NaCl, 0.1 mM EDTA, 1% NP40 and 1 mM dithiothreitol with protease inhibitor mixture and the association of TOP2α to chromatin was analysed by IB. Antibodies for TOP2α, (1:3,000; Abcam, ab52934), Flag (1:5,000; Sigma, F3165), USP10 (1:3,000; Cell Signaling, 8501), mouse Rnf168 (1:3,000; home made), mouse Brca1 (1:500; home made) Histone H4 (1:3,000; Millipore, 05-858) and Lamin B (1:500; Santa Cruz, 3739) were used. All uncropped western blots can be found in Supplementary Fig. 14.

### *In vivo* ubiquitylation assay

Cells were lysed into fraction I and II as described earlier. Mixed cell lysates were immunoprecipitated with anti-TOP2α antibody (1:3,000; NeoMarkers; 1st IP). The immunocomplexes were denatured in Laemmli's sample buffer to dissociate contaminant proteins and TOP2α was reimmunoprecipitated (2nd IP) in denatured conditions (2 mM Tris-HCl (pH 7.5), 5 mM EDTA, 150 mM NaCl, 1% NP40, 1% deoxycholate, 0.025% SDS, 1 mM PMSF and protease inhibitor cocktail tablets). IB analyses were performed with anti-Ub (1:2,000; FK2; Biomol international), anti-TOP2α (NeoMarkers), and horseradish peroxidase-conjugated antibodies to mouse (1:3,000, Cell Signaling; 7076) and an enhanced chemiluminescence system (ECL, Amersham).

### Intracellular ubiquitylation assay

Calcium phosphate was used to transfect HEK293T cells with expression vectors for mouse RNF168 (WT and C21S mutant), HA-tagged WT or mutated mouse Rnf168, HA-RNF8, Flag-TOP2α, Flag-TOP2α−ϰR, HA-USP10 and WT or mutated HA-Ub (Addgene: pRK5-HA-Ub-WT (ID 17608), pRK5-HA-Ub-K48 [ID 17605), pRK5-HA-Ub-K63 (ID 17606)). 48 h post-transfection, cells were lysed in the modified RIPA buffer described earlier. IP was performed using anti-Flag, anti-HA (1:1,000; Santa Cruz, 080211) or anti-Myc (1:1,000; 9E10 Santa Cruz, sc40) antibodies and Protein A-Sepharose.

### *In vitro* ubiquitylation assays

Recombinant RNF168 (ref. 15; 0.5–2 μg), recombinant TOP2α (5 μg, Topogen), UBE1 (0.1 μg; E1), UBE2E2 (0.2 μg; E2), Ub (5 μg) and ATP (5 mM) were mixed and the reactions were incubated at 30 °C for 90 min in buffer containing 50 mM Tris/HCl (pH 7.5), 5 mM MgCl2, and 1 mM DTT, and examined by IB.

### Assessment of nuclear abnormalities

Cells grown on glass coverslips were fixed with 2% paraformaldehyde for 5 min at room temperature, stained with DAPI for 5 min and then mounted with Mowiol mounting solution. More than 1,000 interphase nuclei were analysed using a Leica DM4000B fluorescence microscope.

### Mitotic inhibition assay of decatenation G2 checkpoint

Asynchronous cells were treated for 15 min with DMSO or 4 μM ICRF-193 (Sigma-Aldrich), followed by incubation in drug-free medium for 2 h. Cells were then trypsinized into a single-cell suspension and fixed with 70% ethanol. Mitotic cells were quantified by flow cytometry using anti-pHH3 (pSer10) antibody (1:100; Cell signaling, 9708) and Propidium Iodide (Sigma-Aldrich).

### Mitotic entry assay of decatenation G2 checkpoint

Cells were plated at a density of 10^6^ cells per 10 cm dish (day 0) and fed with fresh medium on day 1. On day 2, cells were treated with 100 ng ml^−1^ colcemid and either DMSO or 4 μM ICRF-193 for 2, 4 and 6 h. Cells were stained with anti-pHH3 antibody and PI and mitotic fraction was identified by flow cytometry.

### EdU labelling and RNF168, TOP2α staining

For EdU labelling, we used Click-It Edu Alexa Fluor 647 Imaging Kit according to manufacturer's recommendations (Invitrogen). EdU was added to growth medium (10 μM) for 4 h and was subsequently detected with Click-It cocktail containing Alexa Fluor 647 azide as one of the components. After washing with 3% BSA in PBS, the sheep polyclonal against RNF168 (1:2,000; R&D systems cat# AF7217) and the rabbit polyclonal against TOP2α (Abcam) were detected using their respective secondary antibodies. Cells were examined using the confocal laser scanning microscope 700 (Carl Zeiss).

### Comet assay

Neutral comet assay was performed as previously described^61^. Single-cell suspensions of MEFs were collected after treatment with 20 μM etoposide for 30 min. They were mixed with 0.7% low-melting agarose and spread on slides pre-coated with 1% normal melting agarose. Slides were then treated with lysis solution (2.5 mM NaCl, 100 mM EDTA, 10 mM Trizma base, 10% DMSO and 1% Triton-X100, pH 9.0) at 4 °C for 72 h. Electrophoresis was performed in Tris-buffer with boric acid and EDTA for 30 min at 32 V. Slides were then air-dried and stained with 2 mg ml^−1^ of ethidium bromide and scored using KOMET 5.0 software (Kinectic Imaging). A minimum of one hundred cells were scored at random for each treatment. Olive Tail Moment (% DNA in the tail/distance between centre of gravity of head and tail) and % Tail DNA were reported as a measure of DNA double-strand breaks.

### Immunofluorescence microscopy

For γ-H2a.x foci experiments, MEFs grown on glass coverslips were fixed with 2% paraformaldehyde for 5 min at room temperature and then incubated overnight at 4 °C with mouse anti-γ-H2a.x (Millipore). Labelling was detected using Alexa Fluor 555-labelled goat-anti-rabbit immunoglobulin secondary antibodies (Molecular Probes). Cells were stained with DAPI for 5 min and then mounted with Mowiol mount solution (Calbiochem). The slides were examined under a Leica DM4000B fluorescence microscope equipped with digital camera (Leica DFC490). Images were acquired under × 100 magnification using Leica Image Manager software.

### Statistical analysis

Data are presented as the mean±s.e.m. The statistical significance of experimental data (*P*-values ≤0.05) was determined using ANOVA with or without Tukey–Kramer test.

### Data availability

The authors declare that the data supporting the findings of this study are available from the corresponding authors upon request.

## Additional information

**How to cite this article:** Guturi, K. K. N. *et al*. RNF168 and USP10 regulate topoisomerase IIα function via opposing effects on its ubiquitylation. *Nat. Commun.* 7:12638 doi: 10.1038/ncomms12638 (2016).

## Supplementary Material

Supplementary Information Supplementary Figures 1-14 and Supplementary Table 1

## References

[b1] Nitiss J. L. DNA topoisomerase II and its growing repertoire of biological functions. Nat. Rev. Cancer 9, 327–337 (2009). 19377505 10.1038/nrc2608 PMC2730144

[b2] Chen S. H., Chan N. L. & Hsieh T. S. New mechanistic and functional insights into DNA topoisomerases. Annu. Rev. Biochem. 82, 139–170 (2013). 23495937 10.1146/annurev-biochem-061809-100002

[b3] Vos S. M., Tretter E. M., Schmidt B. H. & Berger J. M. All tangled up: how cells direct, manage and exploit topoisomerase function. Nat. Rev. Mol. Cell Biol. 12, 827–841 (2011). 22108601 10.1038/nrm3228 PMC4351964

[b4] Damelin M., Sun Y. E., Sodja V. B. & Bestor T. H. Decatenation checkpoint deficiency in stem and progenitor cells. Cancer Cell 8, 479–484 (2005). 16338661 10.1016/j.ccr.2005.11.004

[b5] Bower J. J. . Topoisomerase IIalpha maintains genomic stability through decatenation G(2) checkpoint signaling. Oncogene 29, 4787–4799 (2010). 20562910 10.1038/onc.2010.232 PMC2928865

[b6] Luo K., Yuan J., Chen J. & Lou Z. Topoisomerase IIalpha controls the decatenation checkpoint. Nat. Cell Biol. 11, 204–210 (2009). 19098900 10.1038/ncb1828 PMC2712943

[b7] Alchanati I. . The E3 ubiquitin-ligase Bmi1/Ring1A controls the proteasomal degradation of Top2alpha cleavage complex-a potentially new drug target. PLoS ONE 4, e8104 (2009). 19956605 10.1371/journal.pone.0008104 PMC2779455

[b8] Eguren M. . A synthetic lethal interaction between APC/C and topoisomerase poisons uncovered by proteomic screens. Cell Rep. 6, 670–683 (2014). 24508461 10.1016/j.celrep.2014.01.017

[b9] Lou Z., Minter-Dykhouse K. & Chen J. BRCA1 participates in DNA decatenation. Nat. Struct. Mol. Biol. 12, 589–593 (2005). 15965487 10.1038/nsmb953

[b10] Shinagawa H., Miki Y. & Yoshida K. BRCA1-mediated ubiquitination inhibits topoisomerase II alpha activity in response to oxidative stress. Antioxid. Redox. Signal 10, 939–949 (2008). 18162055 10.1089/ars.2007.1851

[b11] Panier S. & Durocher D. Push back to respond better: regulatory inhibition of the DNA double-strand break response. Nat. Rev. Cancer 13, 661–672 (2013). 10.1038/nrm3659 24002223

[b12] Jackson S. P. & Bartek J. The DNA-damage response in human biology and disease. Nature 461, 1071–1078 (2009). 19847258 10.1038/nature08467 PMC2906700

[b13] Ciccia A. & Elledge S. J. The DNA damage response: making it safe to play with knives. Mol. Cell 40, 179–204 (2010). 20965415 10.1016/j.molcel.2010.09.019 PMC2988877

[b14] Mattiroli F. . RNF168 ubiquitinates K13-15 on H2A/H2AX to drive DNA damage signaling. Cell 150, 1182–1195 (2012). 22980979 10.1016/j.cell.2012.08.005

[b15] Gatti M. . RNF168 promotes noncanonical K27 ubiquitination to signal DNA damage. Cell Rep. 10, 226–238 (2015). 25578731 10.1016/j.celrep.2014.12.021

[b16] Stewart G. S. . RIDDLE immunodeficiency syndrome is linked to defects in 53BP1-mediated DNA damage signaling. Proc. Natl Acad. Sci. USA 104, 16910–16915 (2007). 17940005 10.1073/pnas.0708408104 PMC2040433

[b17] Stewart G. S. . The RIDDLE syndrome protein mediates a ubiquitin-dependent signaling cascade at sites of DNA damage. Cell 136, 420–434 (2009). 19203578 10.1016/j.cell.2008.12.042

[b18] Devgan S. S. . Homozygous deficiency of ubiquitin-ligase ring-finger protein RNF168 mimics the radiosensitivity syndrome of ataxia-telangiectasia. Cell Death Differ. 18, 1500–1506 (2011). 21394101 10.1038/cdd.2011.18 PMC3178430

[b19] Bohgaki T. . Genomic instability, defective spermatogenesis, immunodeficiency, and cancer in a mouse model of the RIDDLE syndrome. PLoS Genet. 7, e1001381 (2011). 21552324 10.1371/journal.pgen.1001381 PMC3084200

[b20] Ishida R. . Inhibition of intracellular topoisomerase II by antitumor bis(2,6-dioxopiperazine) derivatives: mode of cell growth inhibition distinct from that of cleavable complex-forming type inhibitors. Cancer Res. 51, 4909–4916 (1991). 1654205

[b21] Pommier Y. Drugging topoisomerases: lessons and challenges. ACS Chem. Biol. 8, 82–95 (2013). 23259582 10.1021/cb300648v PMC3549721

[b22] Hajji N., Pastor N., Mateos S., Dominguez I. & Cortes F. DNA strand breaks induced by the anti-topoisomerase II bis-dioxopiperazine ICRF-193. Mutat. Res. 530, 35–46 (2003). 14563529 10.1016/s0027-5107(03)00135-0

[b23] Park I. & Avraham H. K. Cell cycle-dependent DNA damage signaling induced by ICRF-193 involves ATM, ATR, CHK2, and BRCA1. Exp. Cell Res. 312, 1996–2008 (2006). 16630610 10.1016/j.yexcr.2006.02.029

[b24] Lukas J., Lukas C. & Bartek J. More than just a focus: the chromatin response to DNA damage and its role in genome integrity maintenance. Nat. Cell Biol. 13, 1161–1169 (2011). 21968989 10.1038/ncb2344

[b25] Kaufmann W. K. Analysis of the topoisomerase II-dependent decatenation G2 checkpoint and checkpoint kinases in human cells. Methods Mol. Biol. 582, 155–166 (2009). 19763949 10.1007/978-1-60761-340-4_13

[b26] Pommier Y. Topoisomerase I inhibitors: camptothecins and beyond. Nat. Rev. Cancer 6, 789–802 (2006). 16990856 10.1038/nrc1977

[b27] Nitiss J. L. Targeting DNA topoisomerase II in cancer chemotherapy. Nat. Rev. Cancer 9, 338–350 (2009). 19377506 10.1038/nrc2607 PMC2748742

[b28] Thorslund T. . Histone H1 couples initiation and amplification of ubiquitin signalling after DNA damage. Nature 527, 389–393 (2015). 26503038 10.1038/nature15401

[b29] Panier S. & Boulton S. J. Double-strand break repair: 53BP1 comes into focus. Nat. Rev. Mol. Cell Biol. 15, 7–18 (2014). 24326623 10.1038/nrm3719

[b30] Komander D. & Rape M. The ubiquitin code. Annu. Rev. Biochem. 81, 203–229 (2012). 22524316 10.1146/annurev-biochem-060310-170328

[b31] Agostinho M. . Conjugation of human topoisomerase 2 alpha with small ubiquitin-like modifiers 2/3 in response to topoisomerase inhibitors: cell cycle stage and chromosome domain specificity. Cancer Res. 68, 2409–2418 (2008). 18381449 10.1158/0008-5472.CAN-07-2092

[b32] Ryu H., Furuta M., Kirkpatrick D., Gygi S. P. & Azuma Y. PIASy-dependent SUMOylation regulates DNA topoisomerase IIalpha activity. J. Cell Biol. 191, 783–794 (2010). 21079245 10.1083/jcb.201004033 PMC2983052

[b33] Denuc A. & Marfany G. SUMO and ubiquitin paths converge. Biochem. Soc. Trans. 38, 34–39 (2010). 20074031 10.1042/BST0380034

[b34] Coelho P. A. . Dual role of topoisomerase II in centromere resolution and aurora B activity. PLoS Biol. 6, e207 (2008). 18752348 10.1371/journal.pbio.0060207 PMC2525683

[b35] Gimenez-Abian J. F. & Clarke D. J. Cytological analysis of chromosome structural defects that result from topoisomerase II dysfunction. Methods Mol. Biol. 582, 189–207 (2009). 19763951 10.1007/978-1-60761-340-4_15

[b36] Bohgaki M. . RNF168 ubiquitylates 53BP1 and controls its response to DNA double-strand breaks. Proc. Natl Acad. Sci. USA 110, 20982–20987 (2013). 24324146 10.1073/pnas.1320302111 PMC3876264

[b37] Dykhuizen E. C. . BAF complexes facilitate decatenation of DNA by topoisomerase IIalpha. Nature 497, 624–627 (2013). 23698369 10.1038/nature12146 PMC3668793

[b38] Draker R., Sarcinella E. & Cheung P. USP10 deubiquitylates the histone variant H2A.Z and both are required for androgen receptor-mediated gene activation. Nucleic Acids Res. 39, 3529–3542 (2011). 21245042 10.1093/nar/gkq1352 PMC3089478

[b39] Ashour M. E., Atteya R. & El-Khamisy S. F. Topoisomerase-mediated chromosomal break repair: an emerging player in many games. Nat. Rev. Cancer 15, 137–151 (2015). 25693836 10.1038/nrc3892

[b40] Akimitsu N. . Enforced cytokinesis without complete nuclear division in embryonic cells depleting the activity of DNA topoisomerase II alpha. Genes Cells 8, 393–402 (2003). 12653966 10.1046/j.1365-2443.2003.00643.x

[b41] Huen M. S., Sy S. M. & Chen J. BRCA1 and its toolbox for the maintenance of genome integrity. Nat. Rev. Mol. Cell Biol. 11, 138–148 (2010). 20029420 10.1038/nrm2831 PMC3899800

[b42] Jacq X., Kemp M., Martin N. M. & Jackson S. P. Deubiquitylating enzymes and DNA damage response pathways. Cell Biochem. Biophys. 67, 25–43 (2013). 23712866 10.1007/s12013-013-9635-3 PMC3756857

[b43] Reyes-Turcu F. E., Ventii K. H. & Wilkinson K. D. Regulation and cellular roles of ubiquitin-specific deubiquitinating enzymes. Annu. Rev. Biochem. 78, 363–397 (2009). 19489724 10.1146/annurev.biochem.78.082307.091526 PMC2734102

[b44] Yuan J., Luo K., Zhang L., Cheville J. C. & Lou Z. USP10 regulates p53 localization and stability by deubiquitinating p53. Cell 140, 384–396 (2010). 20096447 10.1016/j.cell.2009.12.032 PMC2820153

[b45] Lin Z. . USP10 antagonizes c-Myc transcriptional activation through SIRT6 stabilization to suppress tumor formation. Cell Rep. 5, 1639–1649 (2013). 24332849 10.1016/j.celrep.2013.11.029 PMC4007576

[b46] Deng M. . Deubiquitination and activation of AMPK by USP10. Mol. Cell 61, 614–624 (2016). 26876938 10.1016/j.molcel.2016.01.010 PMC4836875

[b47] Elia A. E. . Quantitative proteomic atlas of ubiquitination and acetylation in the DNA Damage Response. Mol. Cell 59, 867–881 (2015). 26051181 10.1016/j.molcel.2015.05.006 PMC4560960

[b48] Zhang M. . Ubiquitin-specific peptidase 10 (USP10) deubiquitinates and stabilizes MutS homolog 2 (MSH2) to regulate cellular sensitivity to DNA damage. J. Biol. Chem. 291, 10783–10791 (2016). 26975374 10.1074/jbc.M115.700047 PMC4865924

[b49] Burgess D. J. . Topoisomerase levels determine chemotherapy response in vitro and in vivo. Proc. Natl Acad. Sci. USA 105, 9053–9058 (2008). 18574145 10.1073/pnas.0803513105 PMC2435590

[b50] Treszezamsky A. D. . BRCA1- and BRCA2-deficient cells are sensitive to etoposide-induced DNA double-strand breaks via topoisomerase II. Cancer Res. 67, 7078–7081 (2007). 17671173 10.1158/0008-5472.CAN-07-0601

[b51] Savage K. I. . Identification of a BRCA1-mRNA splicing complex required for efficient DNA repair and maintenance of genomic stability. Mol. Cell 54, 445–459 (2014). 24746700 10.1016/j.molcel.2014.03.021 PMC4017265

[b52] Aparicio T., Baer R., Gottesman M. & Gautier J. MRN, CtIP, and BRCA1 mediate repair of topoisomerase II-DNA adducts. J. Cell Biol. 212, 399–408 (2016). 26880199 10.1083/jcb.201504005 PMC4754713

[b53] Prakash R., Zhang Y., Feng W. & Jasin M. Homologous recombination and human health: the roles of BRCA1, BRCA2, and associated proteins. Cold Spring Harb. Perspect. Biol. 7, a016600 (2015). 25833843 10.1101/cshperspect.a016600 PMC4382744

[b54] Lallemand-Breitenbach V. . Arsenic degrades PML or PML-RARalpha through a SUMO-triggered RNF4/ubiquitin-mediated pathway. Nat. Cell Biol. 10, 547–555 (2008). 18408733 10.1038/ncb1717

[b55] Kessner D., Chambers M., Burke R., Agus D. & Mallick P. ProteoWizard: open source software for rapid proteomics tools development. Bioinformatics 24, 2534–2536 (2008). 18606607 10.1093/bioinformatics/btn323 PMC2732273

[b56] Shteynberg D. . iProphet: multi-level integrative analysis of shotgun proteomic data improves peptide and protein identification rates and error estimates. Mol. Cell Proteomics. 10, M111 007690 (2011). 21876204 10.1074/mcp.M111.007690 PMC3237071

[b57] Liu G. . ProHits: integrated software for mass spectrometry-based interaction proteomics. Nat. Biotechnol. 28, 1015–1017 (2010). 20944583 10.1038/nbt1010-1015 PMC2957308

[b58] Eng J. K., Jahan T. A. & Hoopmann M. R. Comet: an open-source MS/MS sequence database search tool. Proteomics 13, 22–24 (2013). 23148064 10.1002/pmic.201200439

[b59] Li L. . Rnf8 deficiency impairs class switch recombination, spermatogenesis, and genomic integrity and predisposes for cancer. J. Exp. Med. 207, 983–997 (2010). 20385750 10.1084/jem.20092437 PMC2867283

[b60] McPherson J. P. . Collaboration of Brca1 and Chk2 in tumorigenesis. Genes Dev. 18, 1144–1153 (2004). 15131084 10.1101/gad.1192704 PMC415639

[b61] Fan R. . Defective DNA strand break repair after DNA damage in prostate cancer cells: implications for genetic instability and prostate cancer progression. Cancer Res. 64, 8526–8533 (2004). 15574758 10.1158/0008-5472.CAN-04-1601

