# Supplementary material for: RNF168 and USP10 regulate topoisomerase IIα function via opposing effects on its ubiquitylation
Source: Nat Commun. 2016 Aug 25;7:12638. doi: 10.1038/ncomms12638 (PMC5007378; doi:10.1038/ncomms12638)
Supplement: Supplementary Information — Supplementary Figures 1-14 and Supplementary Table 1 [file ncomms12638-s1.pdf]

Supplementary Information

Supplementary Figures

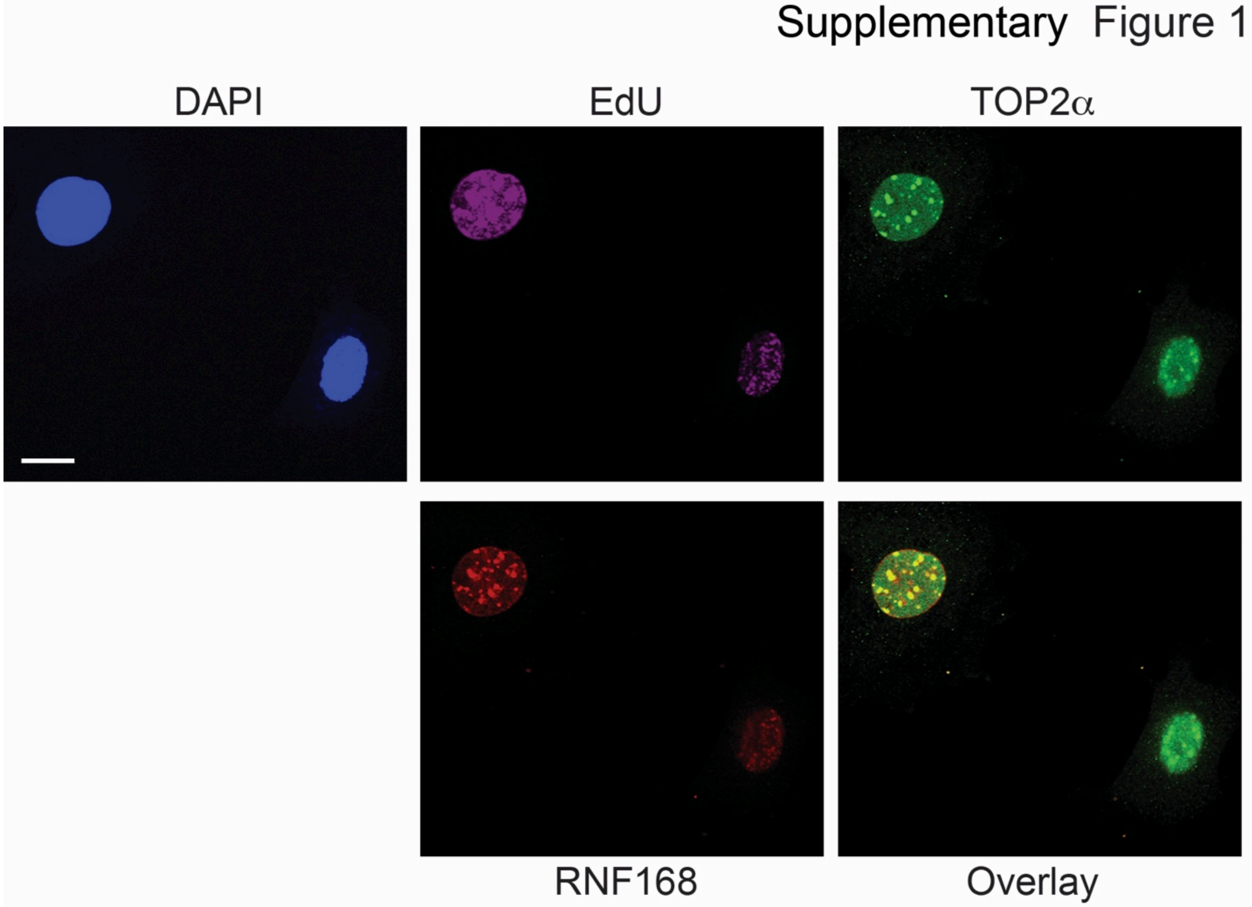

Supplementary Figure 1. TOP2α colocalization with RNF168 in mid S phase. Cells treated with EdU were examined for localization patterns of TOP2α (Alexa Fluor 488) and RNF168 (Alexa Fluor 594) using confocal microscopy. Representative images showing colocalization of TOP2α and RNF168 in S phase. Scale bar: 20μm.

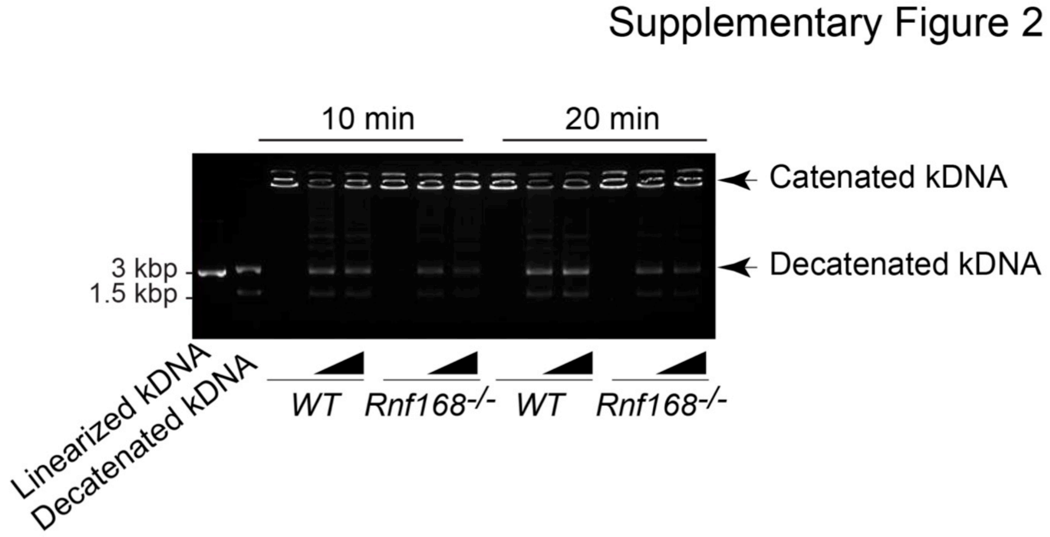

Supplementary Figure 2. RNF168 Stimulates DNA decatenation. *In vitro* decatenation assaywas carried out for 10 or 20 min using different amount of nuclear extracts from *Rnf168*$^{-/-}$ and *WT* thymocytes. Catenated and decatenated DNA were examined by electrophoresis using 1% agarose gel.

Supplementary Figure 3

a

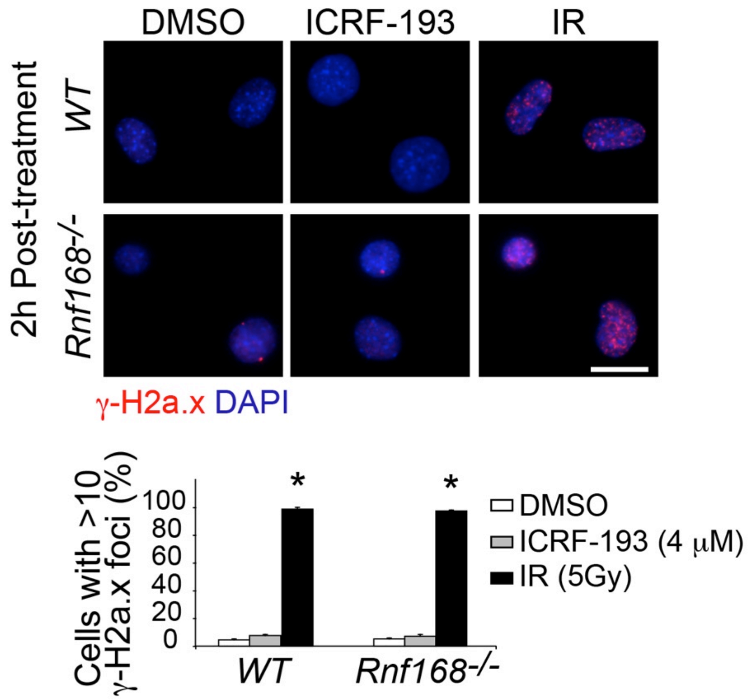

b

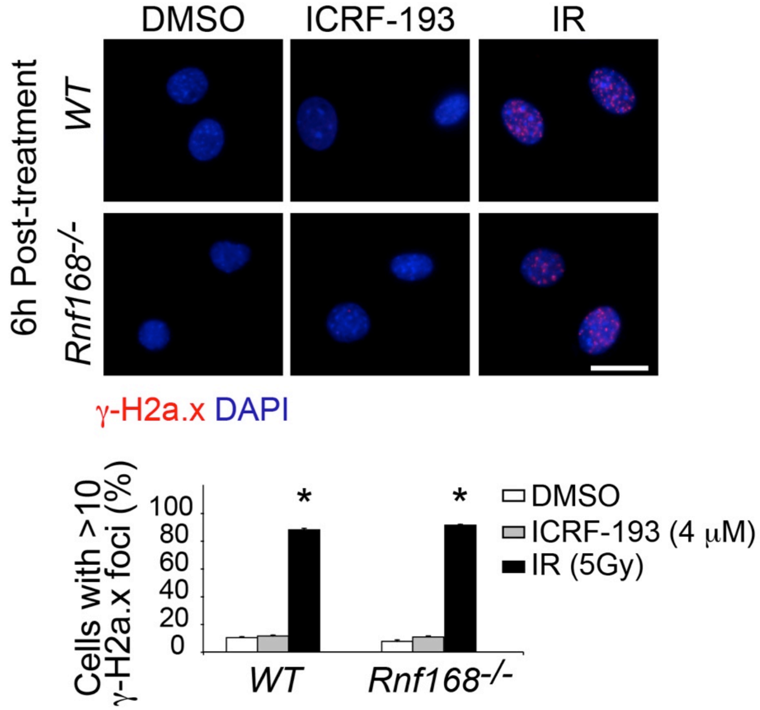

Supplementary Figure 3. In contrast to irradiation, ICRF-193 does not induce DSBs in MEFs. *WT* and *Rnf168*$^{-/-}$ MEFs were treated for 15 min with DMSO or ICRF-193 (4 μM). 2h (**a**) or 6h (**b**) post-treatment, cells were fixed and stained with anti-γ-H2a.x and DAPI. Cells treated with 5Gy IR were used as positive controls for DNA damage. Scale bars: 20 μm. Histograms show the percentage of cells with more than 10 γ-H2a.x foci (mean±SEM, n=3). ANOVA test with Tukey-Kramer test: * $P<0.05$ compared to DMSO treated controls.

Supplementary Figure 4

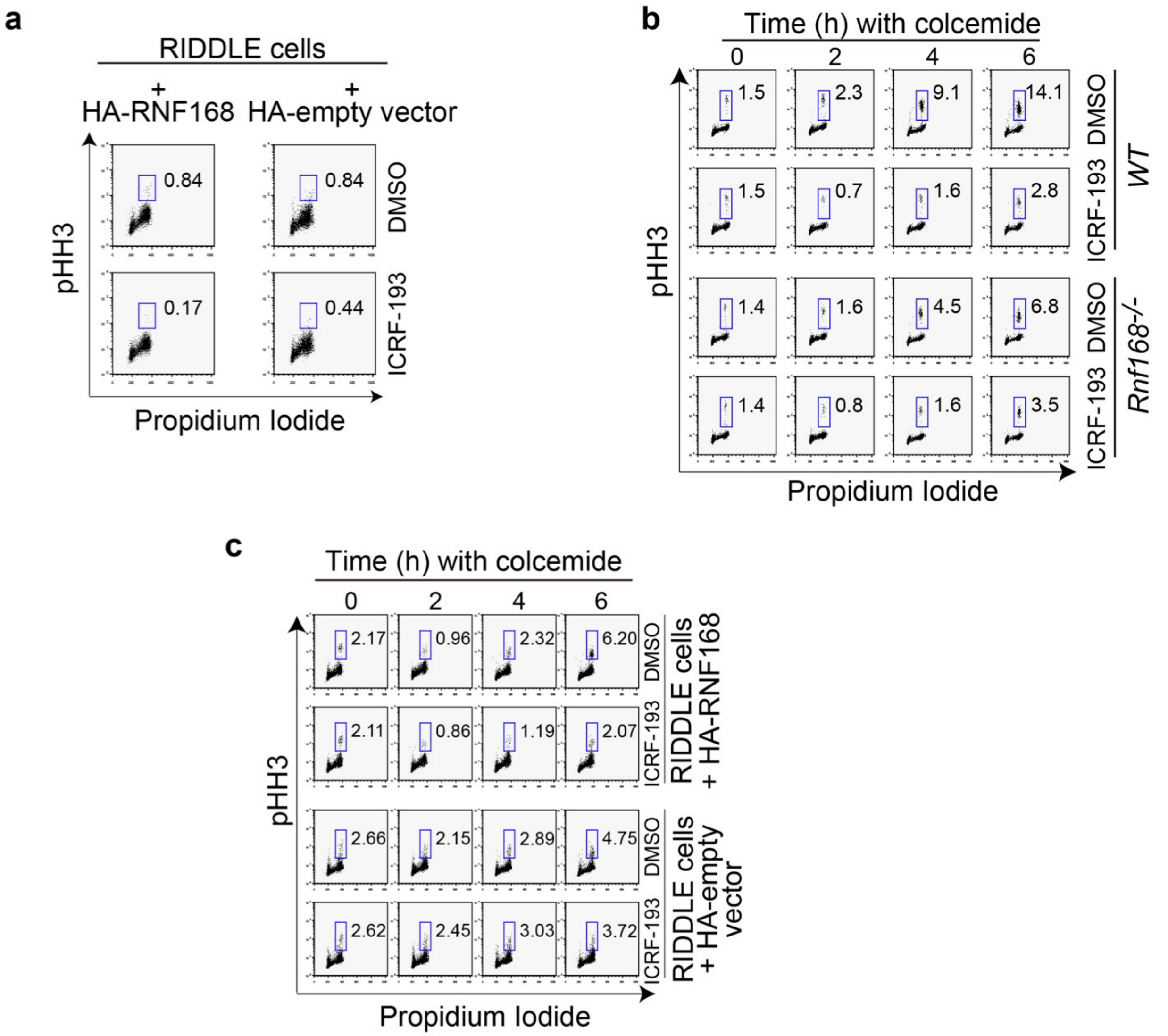

Supplementary Figure 4. RNF168 is required for the stimulation of decatenation G2 checkpoint. (**a**) Representative FACS analysis of mitotic inhibition assay of decatenation G2 checkpoint in human RIDDLE cells reconstituted with HA-RNF168 or HA-empty vector. (**b**,**c**) Representative FACS analysis of the mitotic entry assay showing $pHH3^{+}$ *WT* and *Rnf168*$^{-/-}$

passage 3 MEFs at the indicated time post-treatment with colcemid with or without 4 μM ICRF-193 (**b**) or RIDDLE cells reconstituted with HA-RNF168 or HA-empty vector (**c**).

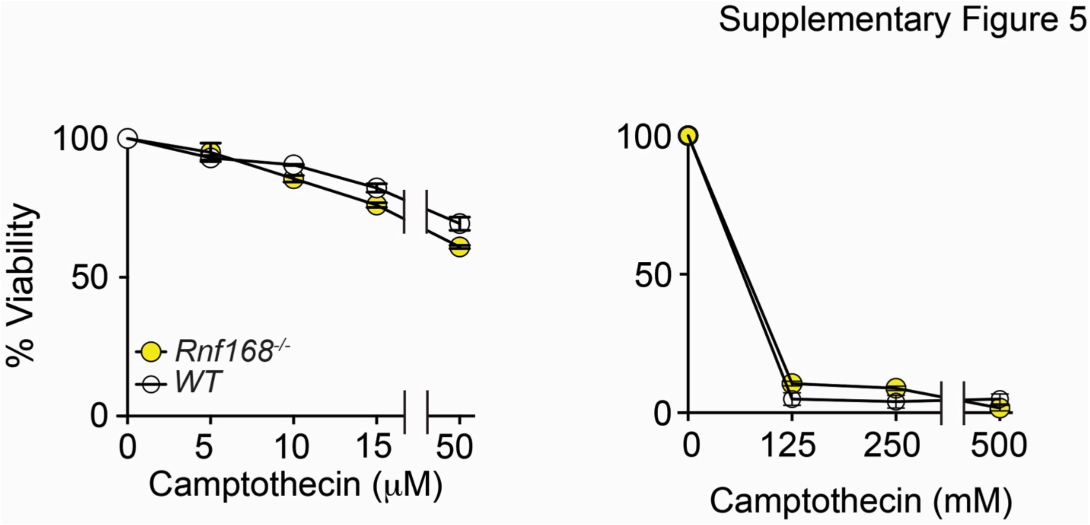

Supplementary Figure 5. The cytotoxic effect of the TOP1 inhibitor camptothecin is not affected by Rnf168 deficiency. (a,b) Sensitivity of *WT* and *Rnf168*$^{-/-}$ MEFs to camptothecin was determined using clonogenic assays. Data are presented as the mean±SEM (n=4). No statistically significant difference was observed between *WT* and *Rnf168*$^{-/-}$ MEFs.

Supplementary Figure 6

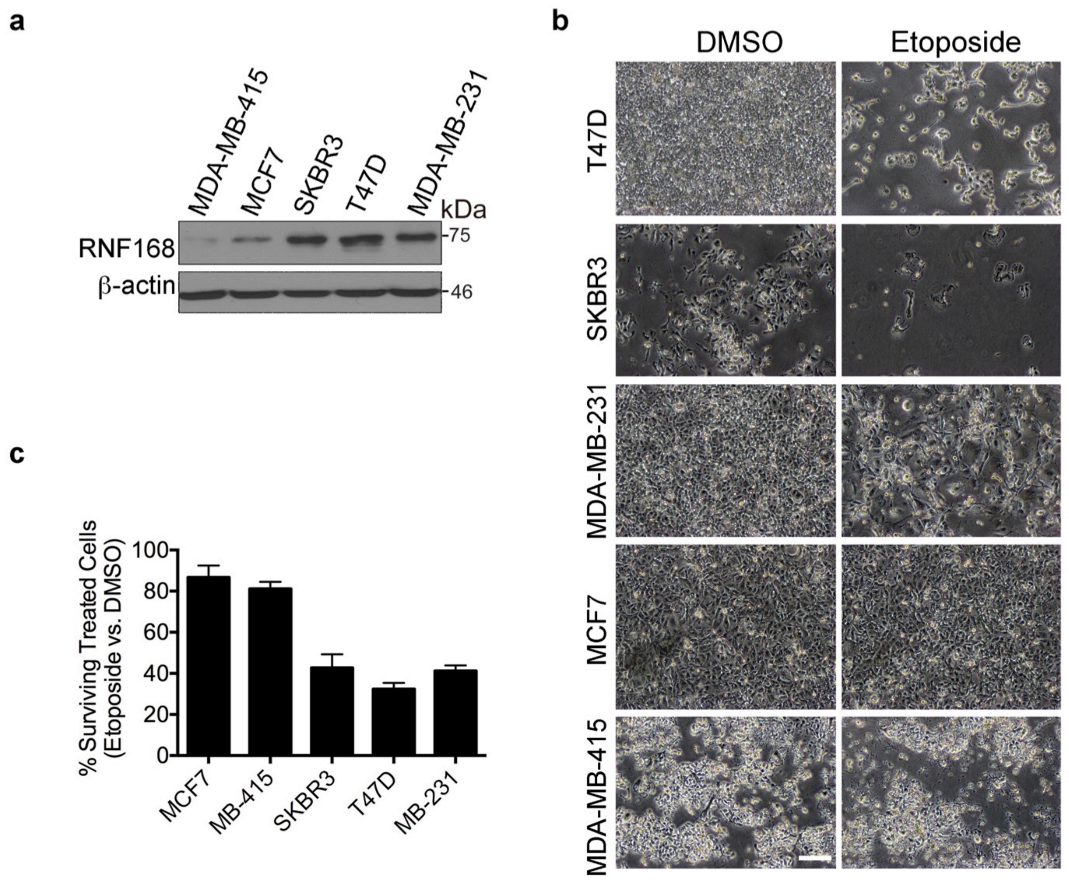

Supplementary Figure 6. Examination of human breast cancer cell lines for the expression of RNF168 and the response to etoposide. (a) The expression level of RNF168 in the indicated breast cancer cell lines was examined by IB. (**b**) Representative images of the indicated breast cancer cell lines treated with 250 nM of etoposide or DMSO and grown on 6 cm dishes for 14 days. (**c**) Cells were counted at day 14 post treatment and the ratio of surviving fractions in response to etoposide compared to DMSO are shown. Data are presented as the mean±SEM (n=2). Scale bar: 200 μm.

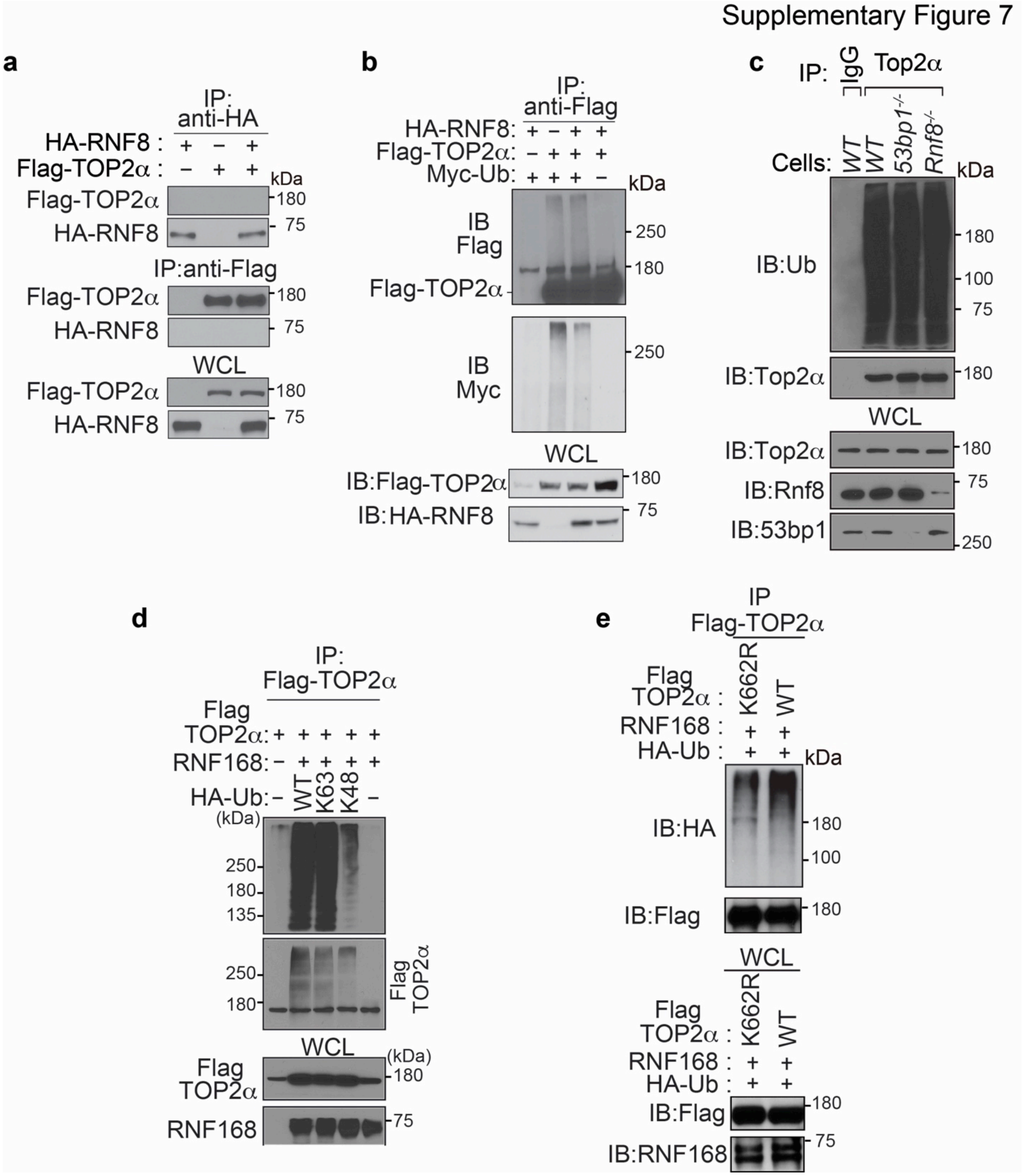

Supplementary Figure 7. Effect of RNF8, RNF168 and 53BP1 on TOP2α ubiquitylation.

(**a**) Intracellular binding assay. HEK293T cells were transfected with HA-RNF8 and Flag-TOP2α expression vectors as indicated. The WCL were immunoprecipitated with anti-HA or

anti-Flag and IB analysis was performed using the indicated antibodies. (**b**) HEK293T cells were transfected with RNF8, Flag-TOP2α and Myc-Ub vectors as indicated. WCL were subjected to IP with anti-Flag and IB analysis was performed using anti-HA antibody to detect ubiquitylated Flag-TOP2α. (**c**) Detection of TOP2α ubiquitylation in *Rnf8*$^{-/-}$, *53bp1*$^{-/-}$ and *WT* MEFs. Anti-TOP2α antibody IP from WCL was immunoblotted using anti-Ub. (d) HEK293T cells were transfected with constructs to express Flag-TOP2α, RNF168, and either HA-Ub-WT (WT), or mutants HA-Ub-K48 (K48) or HA-Ub-K63 (K63) as indicated. Anti-Flag IPs performed in native conditions using WCL were subjected IB analysis with anti-HA and anti-Flag antibodies. (**e**) HEK293T cells were transfected with RNF168, Flag-TOP2α, Flag-TOP2α-K662R and HA-Ub vectors as indicated. WCL were subjected to IP with anti-Flag and IB analysis was performed using anti-HA antibody to detect ubiquitylated Flag-TOP2α.

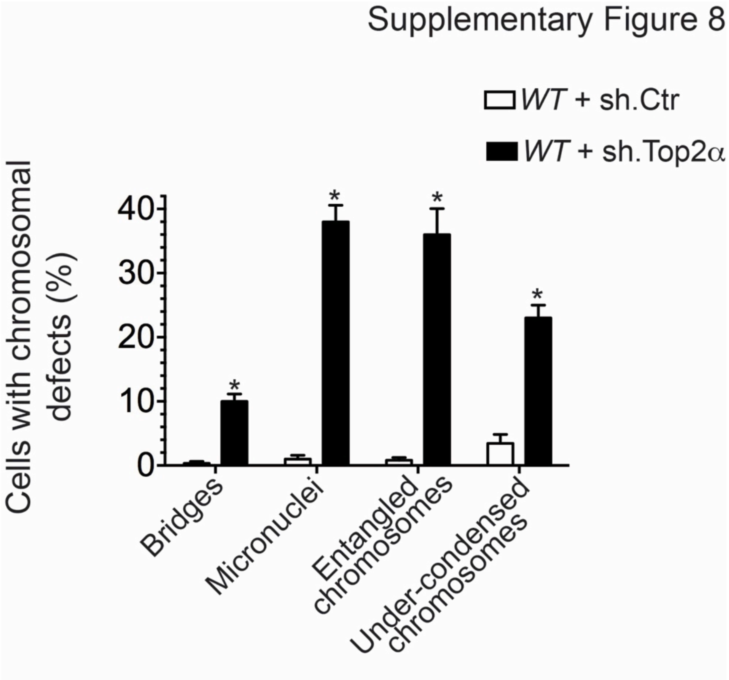

Supplementary Figure 8. Deficiency of TOP2α impairs chromosome segregation and promotes chromosome entanglement and under-condensation. Histograms show quantification of the frequency of cells with chromosome bridges, micronuclei, undercondensed and entangled chromosomes. *WT* MEFs were transduced with lentiviral ShRNA control (sh.Ctr) or ShRNA for Top2α (sh.Top2α). (mean±SEM, n=3). * $P<0.05$.

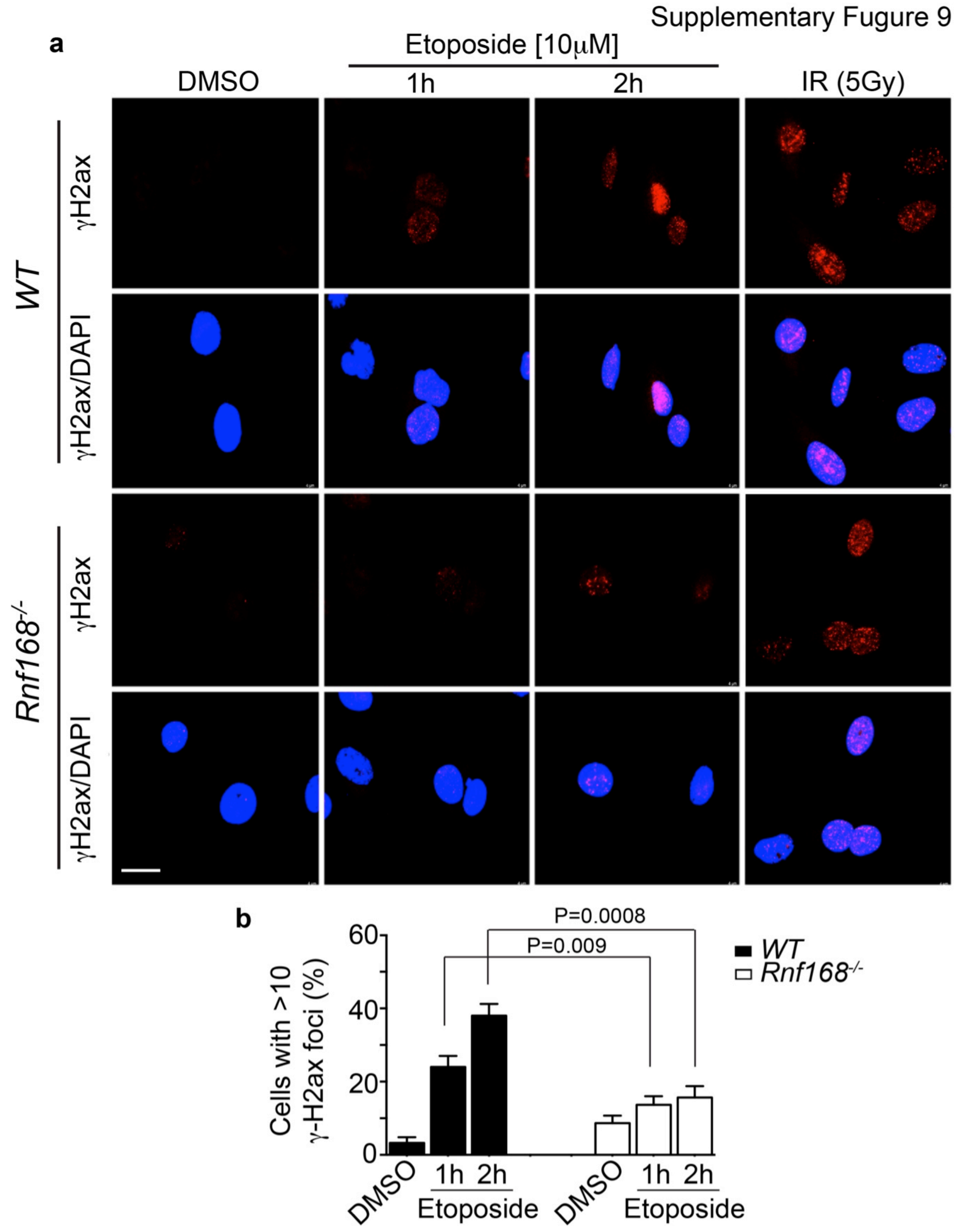

Supplementary Figure 9. ***Rnf168**$^{-/-}$* **MEFs show reduced frequency of DSBs in response to etoposide**. (**a**) *WT* and *Rnf168*$^{-/-}$ MEFs were treated for 15 min with DMSO or etoposide

(10μM), fixed 1h or 2h later and stained with anti-γ-H2ax and DAPI. MEFs, 1h post IR treatment (5Gy), were used as positive controls for DNA damage. (**b**) Histograms show the percentage of cells with more than 10 γ-H2ax foci (mean±SEM, n=3). P: ANOVA test with Tukey-Kramer test (*Rnf168*$^{-/-}$ MEFs compared to *WT* MEFs). Scale bar: 0.8 μm.

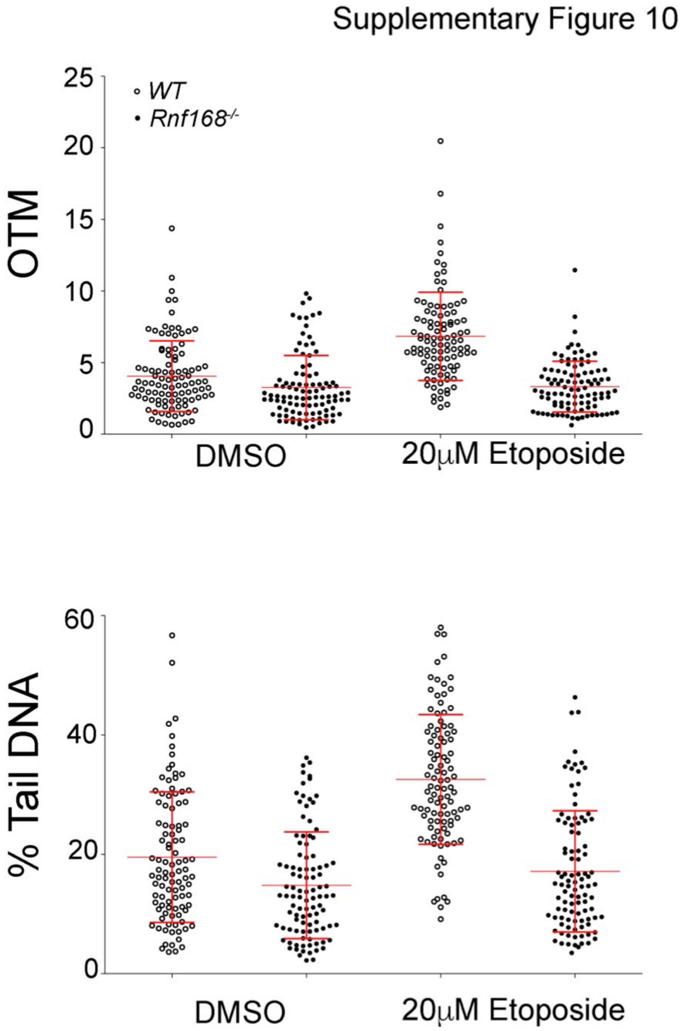

Supplementary Figure 10. Neutral comet assay data indicate fewer etoposide generated

DSBs in *Rnf168*$^{-/-}$ MEFs compared to *WT* controls. The assay was performed using cells 30 min post etoposide (20 µM) treatment. A lower increase in the mean olive tail moment (**Upper panel**) and in the % tail DNA (**Lower pan**el) was observed in *Rnf168*$^{-/-}$ MEFs compared to *WT* controls

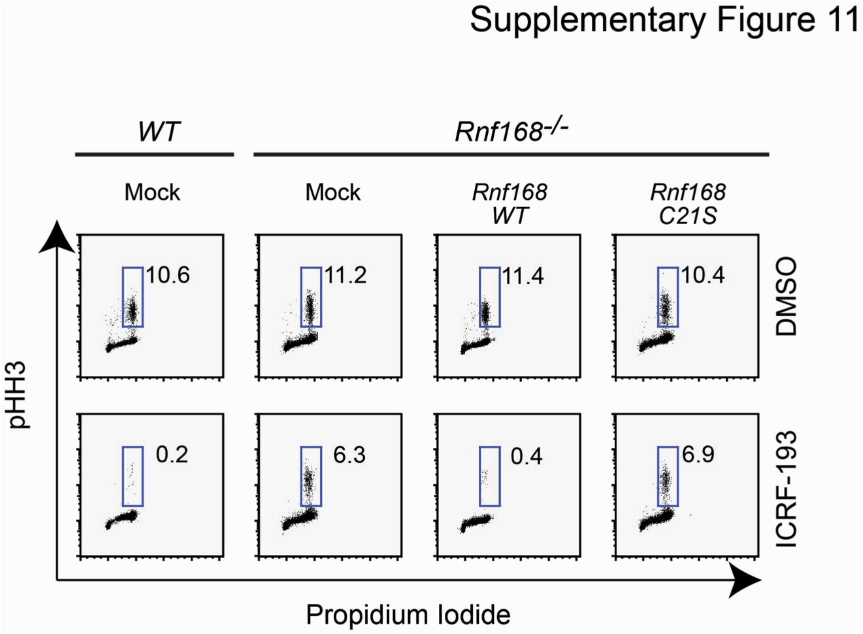

Supplementary Figure 11. The E3 Ub ligase function of RNF168 stimulates decatenation G2 checkpoint. Representative data of the mitotic entry assay of decatenation G2 checkpoint in *WT* 3T3 MEFs and *Rnf168*$^{-/-}$ 3T3 MEFs restored with either empty vector (mock), *Rnf168-WT* or *Rnf168-C21S* mutant. Data are shown for the time-point 6h post-treatment with colcemid ± 4 µM ICRF-193.

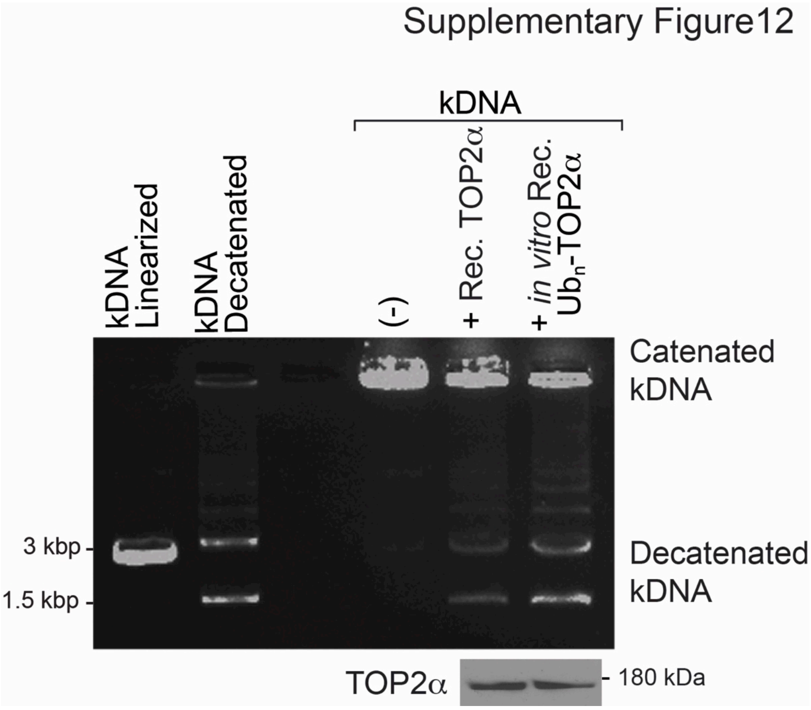

Supplementary Figure 12. Increased decatenation activity of recombinant TOP2α following its *in vitro* ubiquitylation by RNF168. A representative agarose gel showing data obtained using *in vitro* kinetoplast DNA-based decatenation assay performed for 10 min with recombinant TOP2α before or after its *in vitro* ubiquitylation by RNF168. Catenated and decatenated kDNA were separated by electrophoresis using 1% agarose. Ub_n-TOP2α: polyubiquitylated TOP2α. (-) No TOP2α added.

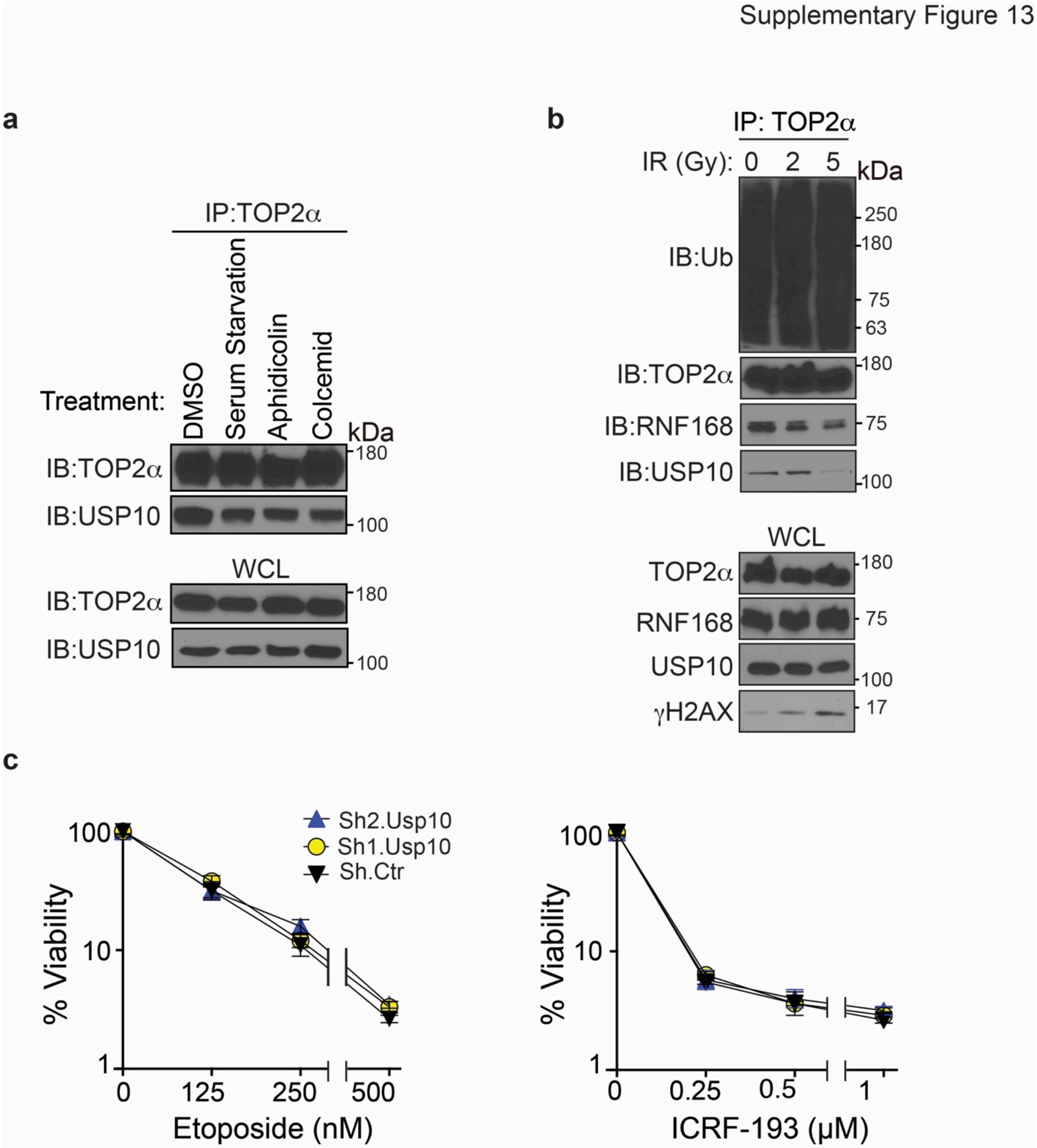

Supplementary Figure 13. Interactions of TOP2α with RNF168 and USP10. **(a)** *WT* MEFs were synchronized at G0/G1 (serum starvation), G1/S (Aphidicolin treatment) and G2/M (Colcemid treatment), and IP using observed no difference between these conditions and Top2α-

Usp10 interaction in nuclear extracts was examined by IP/IB as indicated. (**b**) Effects of irradiation on Top2α ubiquitylation and interaction with Usp10 and Rnf168. IP of Top2α from nuclear extract of *WT* MEFs either untreated or 1h post-IR (2 or 5Gy) were examined by IB as indicated. Expression of the indicated proteins in WCL was examined by IB. (**c**) Graphs represent data from clonogenic assays showing sensitivity to etoposide and ICRF-193 of *WT* MEFs knocked down for Usp10 (sh1.Usp10 and sh2.Usp10) compared to controls (Sh.Ctr: ShRNA control). mean±SEM (2). $P>0.05$.

Supplementary Fig. 14 (Uncropped Blots)

Figure 1b

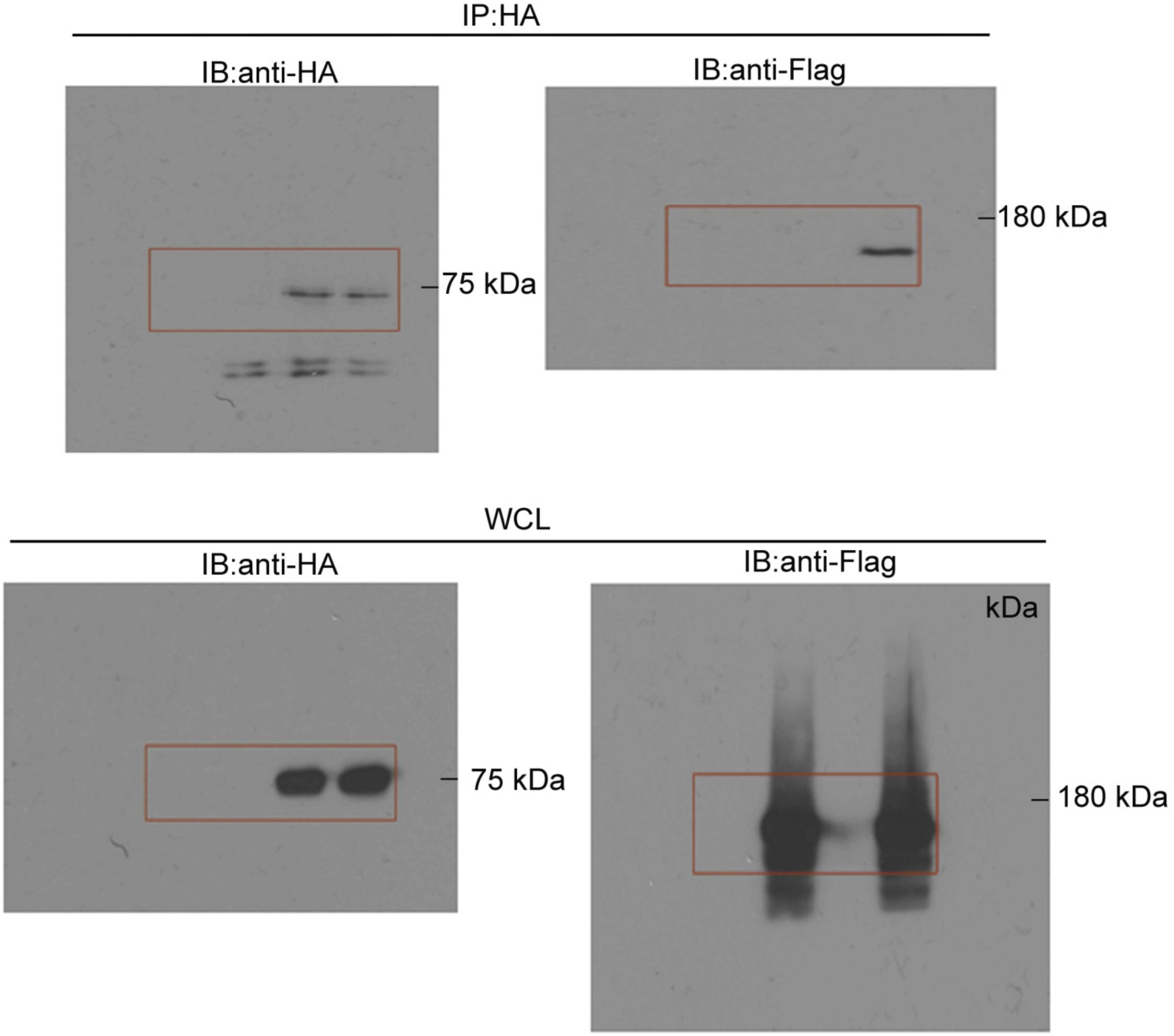

Figure 1c

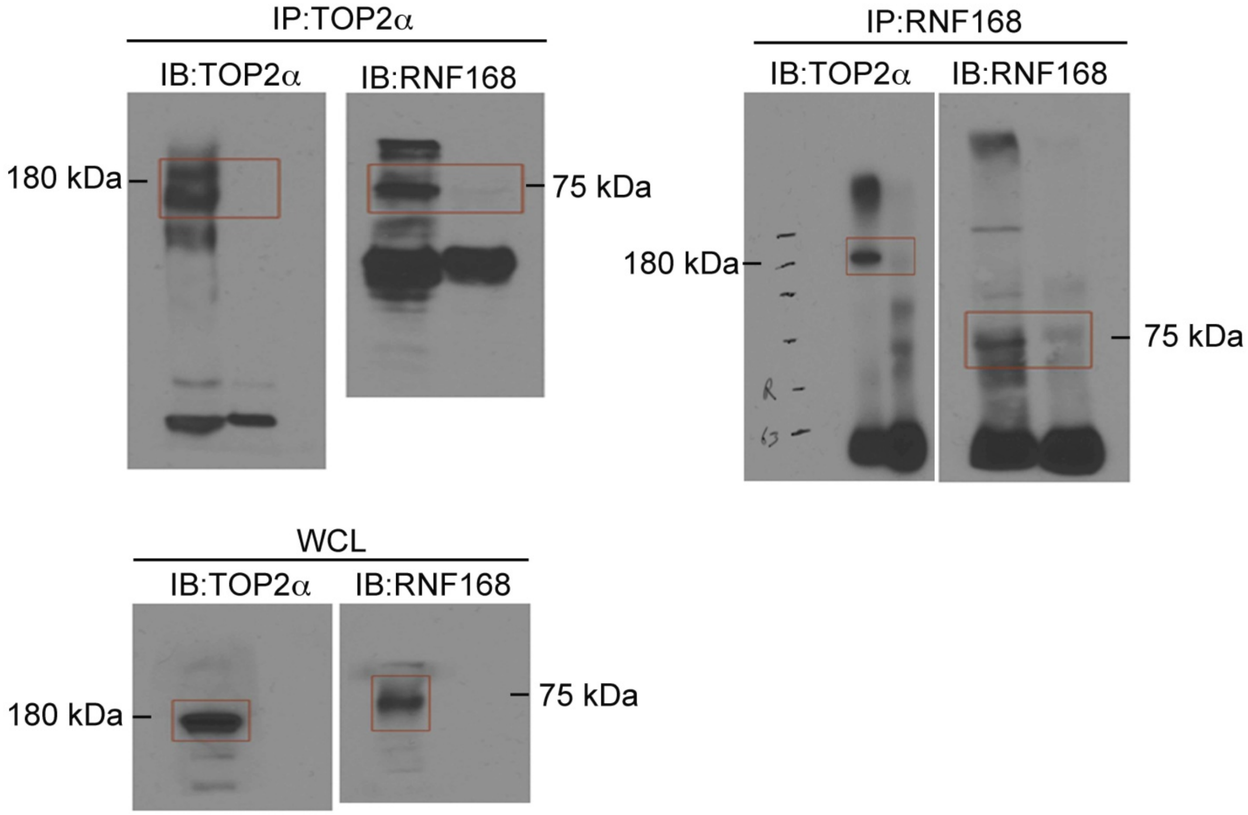
IP:TOP2α
IB:TOP2α
IB:RNF168
180 kDa
75 kDa
IP:RNF168
IB:TOP2α
IB:RNF168
180 kDa
75 kDa
WCL
IB:TOP2α
IB:RNF168
180 kDa
75 kDa

Figure 2a

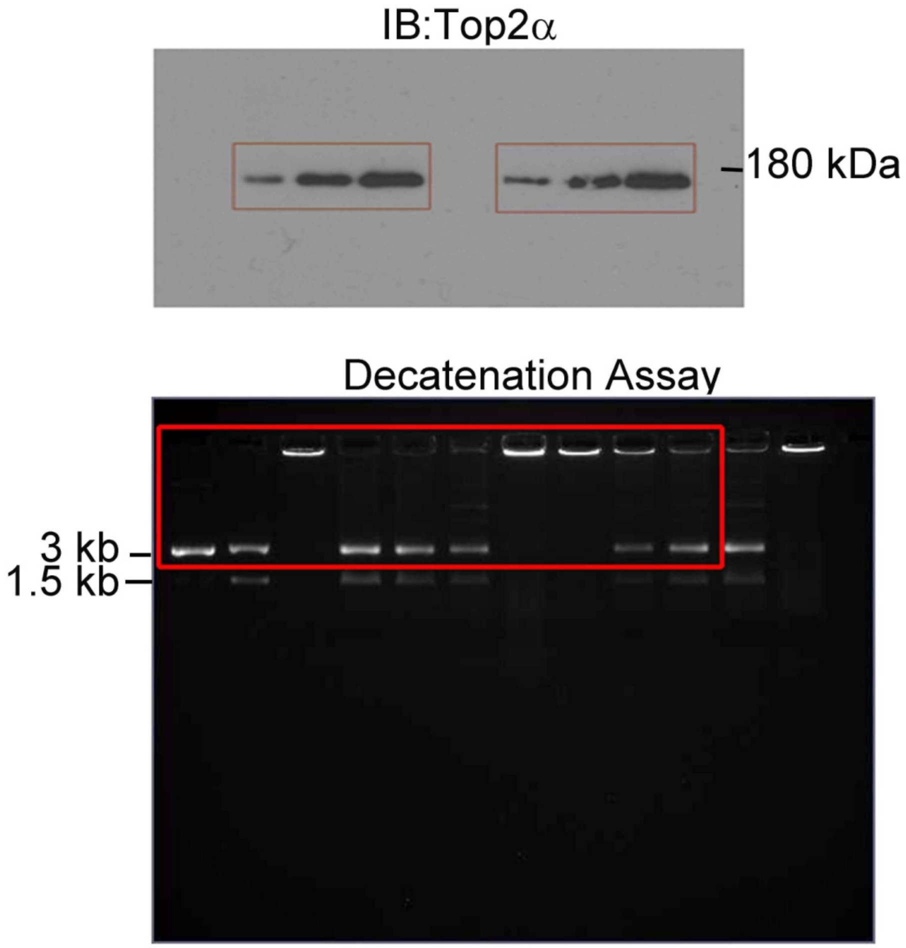

Figure 4a

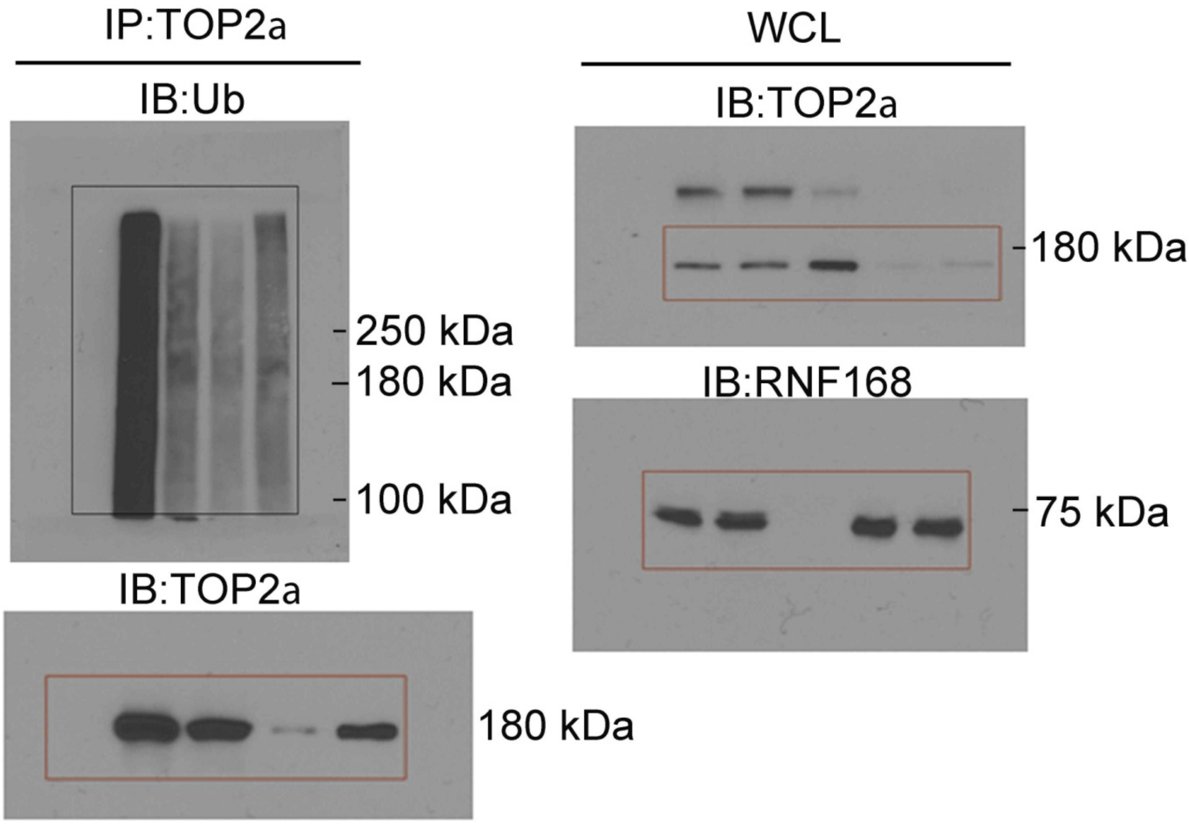

IP:TOP2a
IB:Ub
250 kDa
180 kDa
100 kDa
IB:TOP2a
180 kDa
WCL
IB:TOP2a
180 kDa
IB:RNF168
75 kDa

Figure 4b

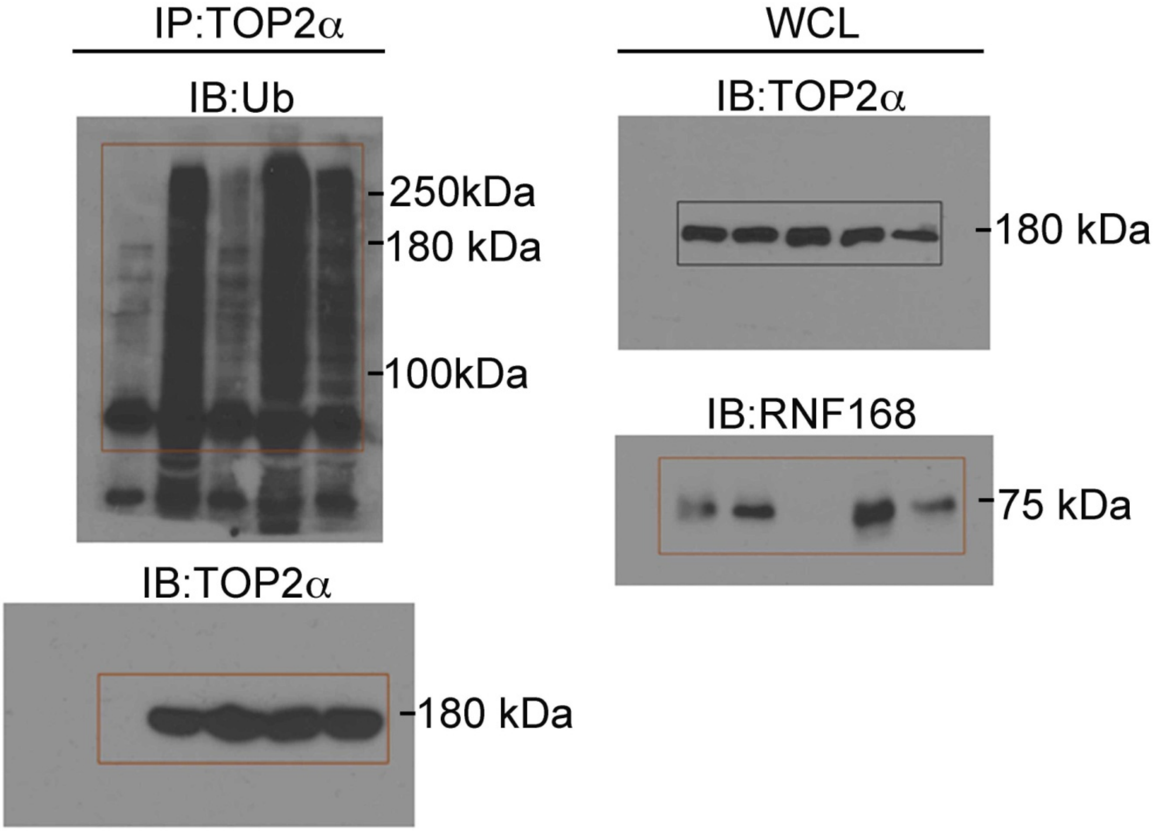

IP:TOP2α
IB:Ub
250kDa
180 kDa
100kDa
IB:TOP2α
180 kDa
WCL
IB:TOP2α
180 kDa
IB:RNF168
75 kDa

Figure 4c

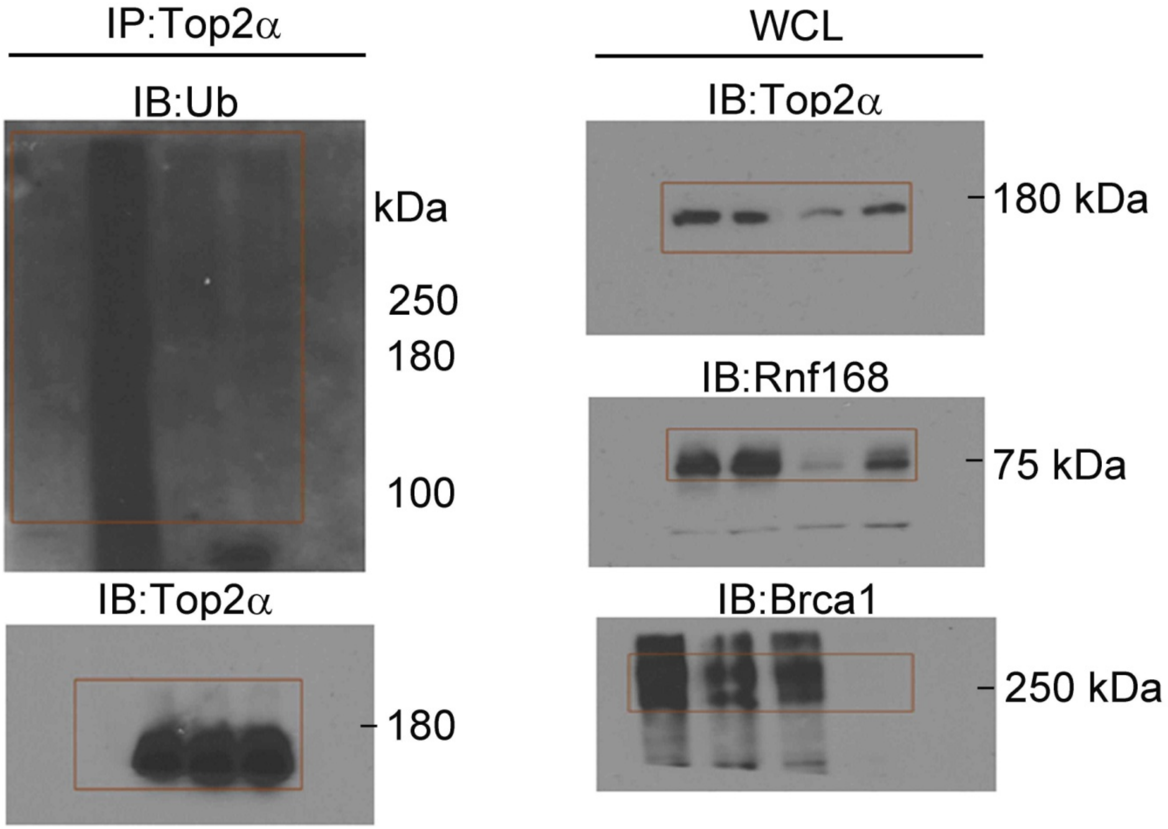
IP:Top2α
IB:Ub
kDa
250
180
100
IB:Top2α
180
WCL
IB:Top2α
180 kDa
IB:Rnf168
75 kDa
IB:Brca1
250 kDa

Figure 4d

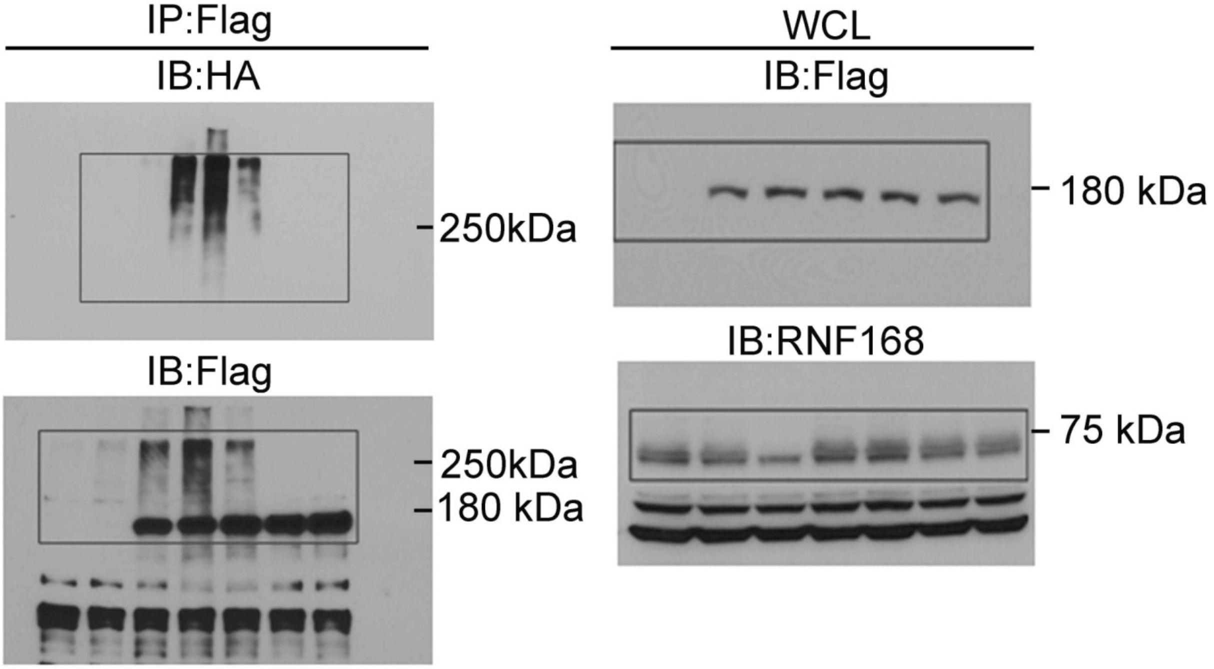
IP:Flag
IB:HA
250kDa
IB:Flag
250kDa
180 kDa
WCL
IB:Flag
180 kDa
IB:RNF168
75 kDa

Figure 4e

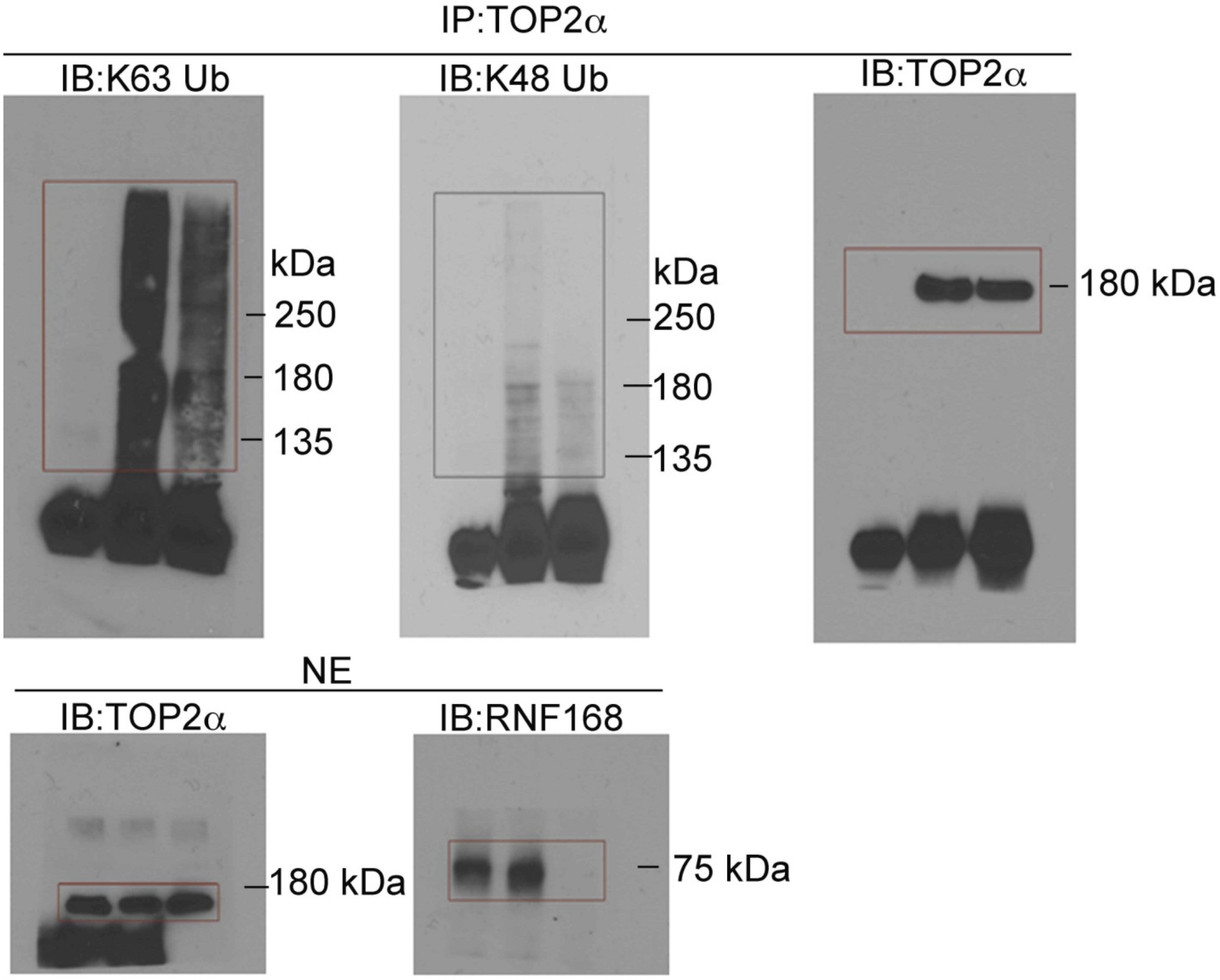
IP:TOP2α
IB:K63 Ub
kDa
250
180
135
IB:K48 Ub
kDa
250
180
135
IB:TOP2α
180 kDa
NE
IB:TOP2α
180 kDa
IB:RNF168
75 kDa

Figure 4f

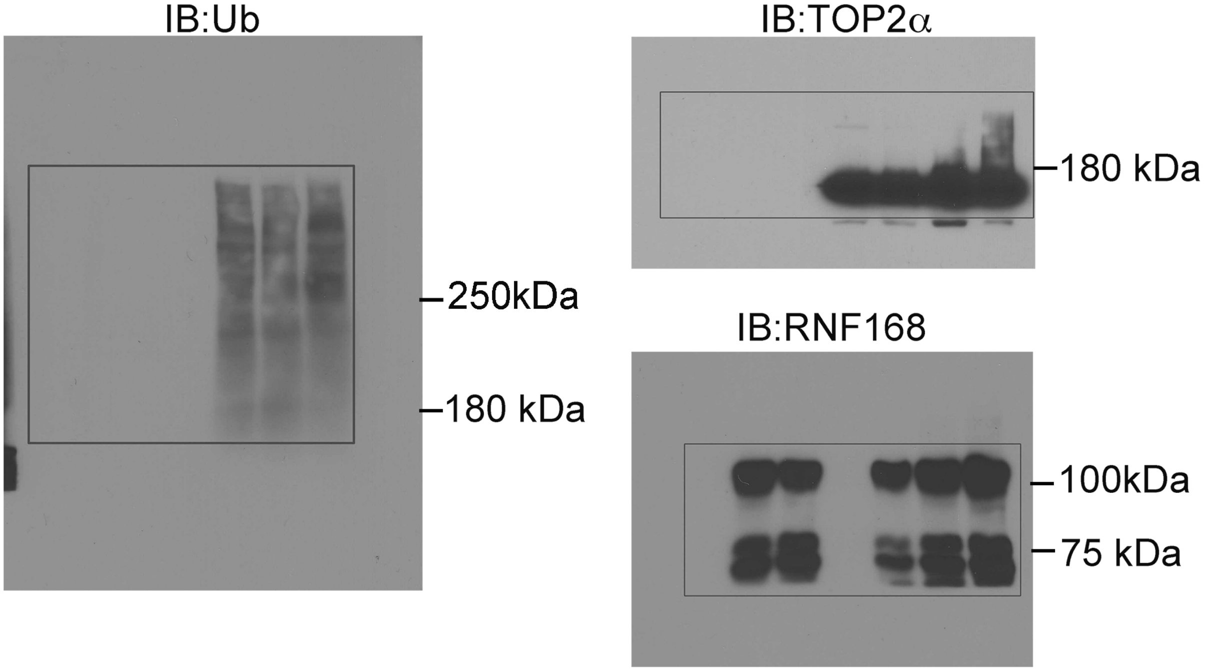

Figure 6a

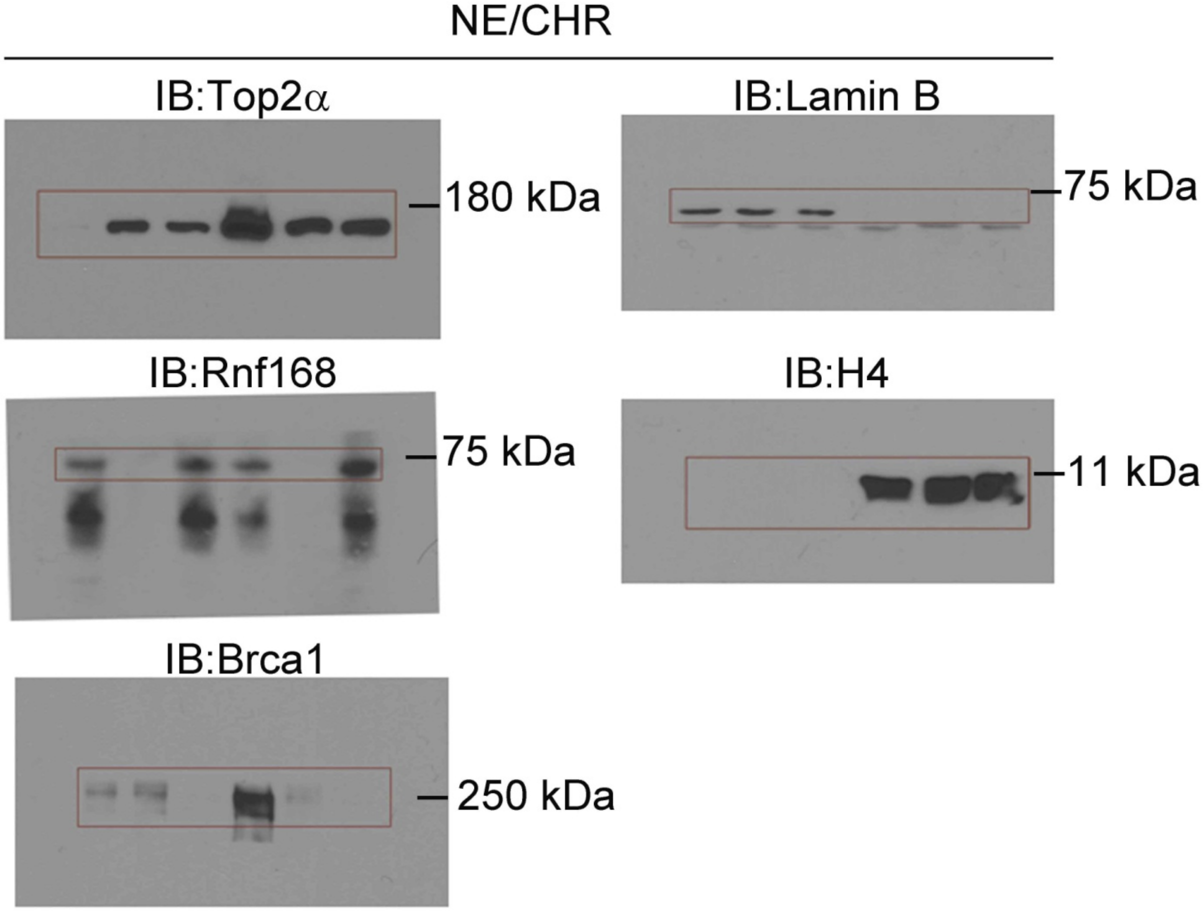
NE/CHR
IB:Top2α
180 kDa
IB:Lamin B
75 kDa
IB:Rnf168
75 kDa
IB:H4
11 kDa
IB:Brca1
250 kDa

Figure 6b

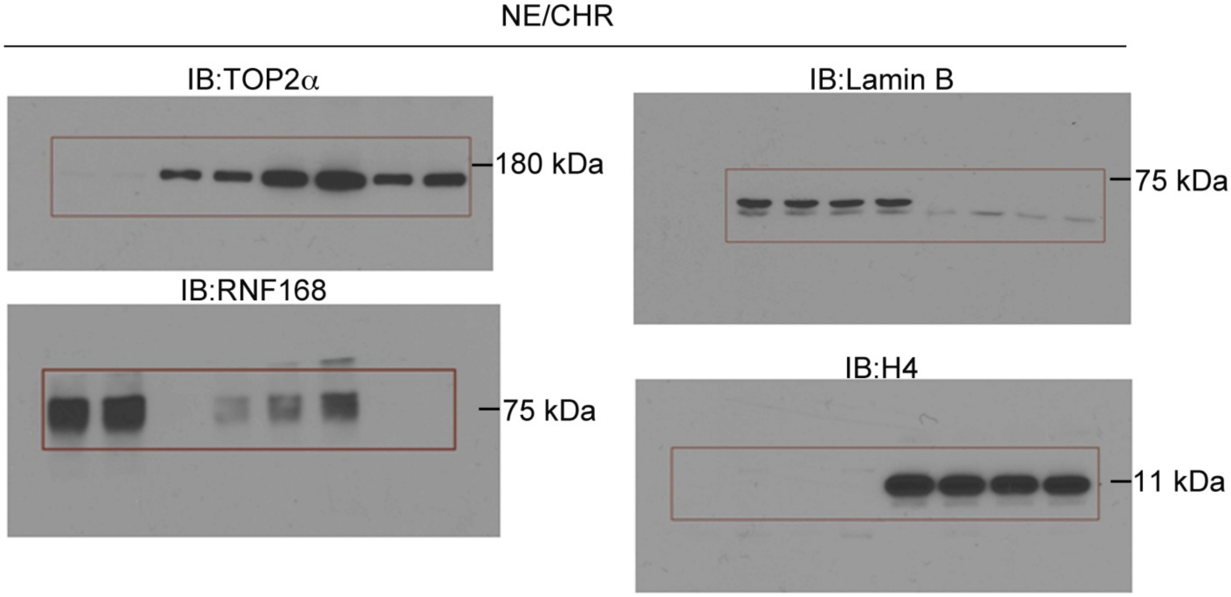

Figure 6c

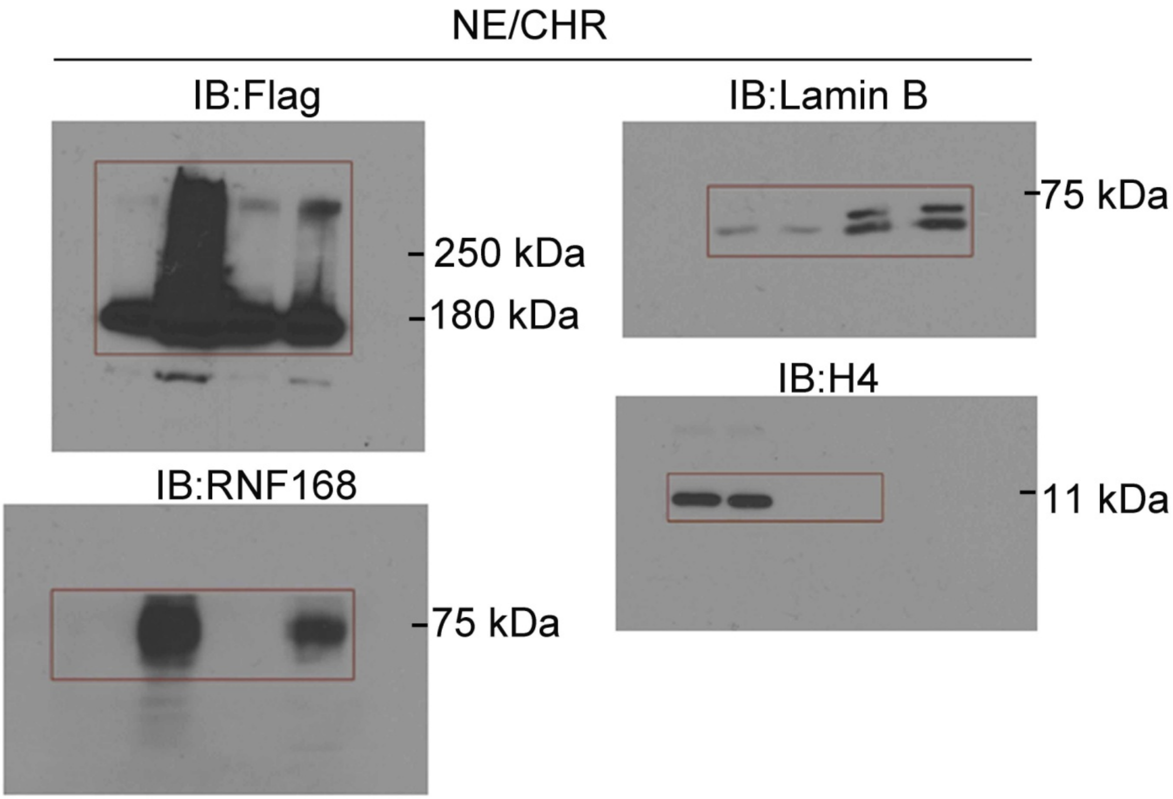

Figure 6d

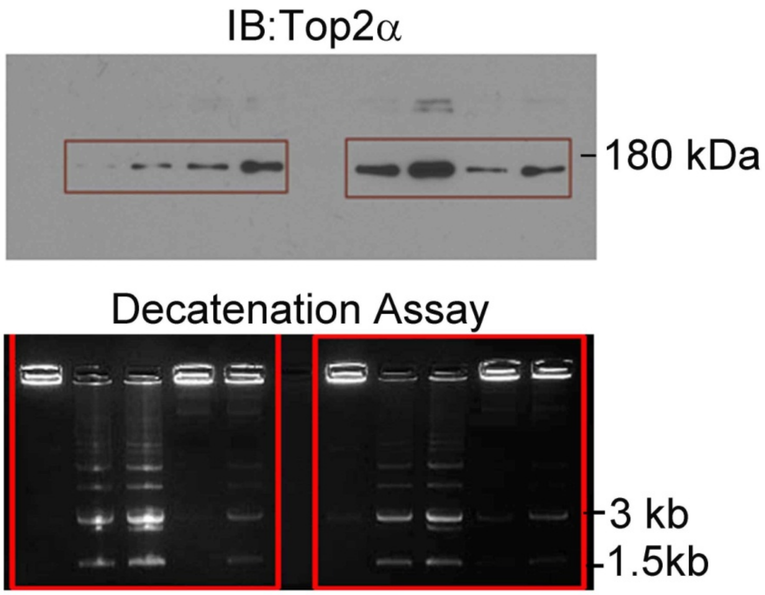

Figure 7a

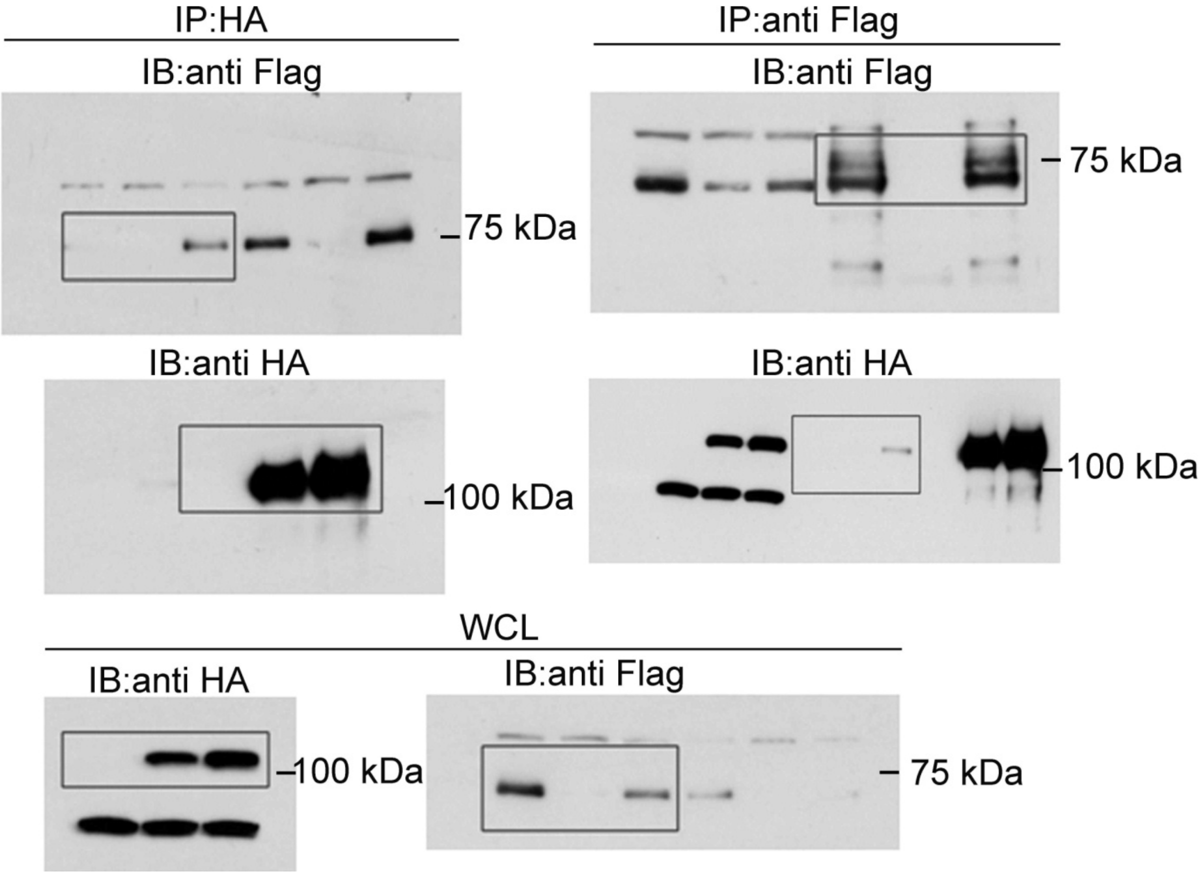

Figure 7b

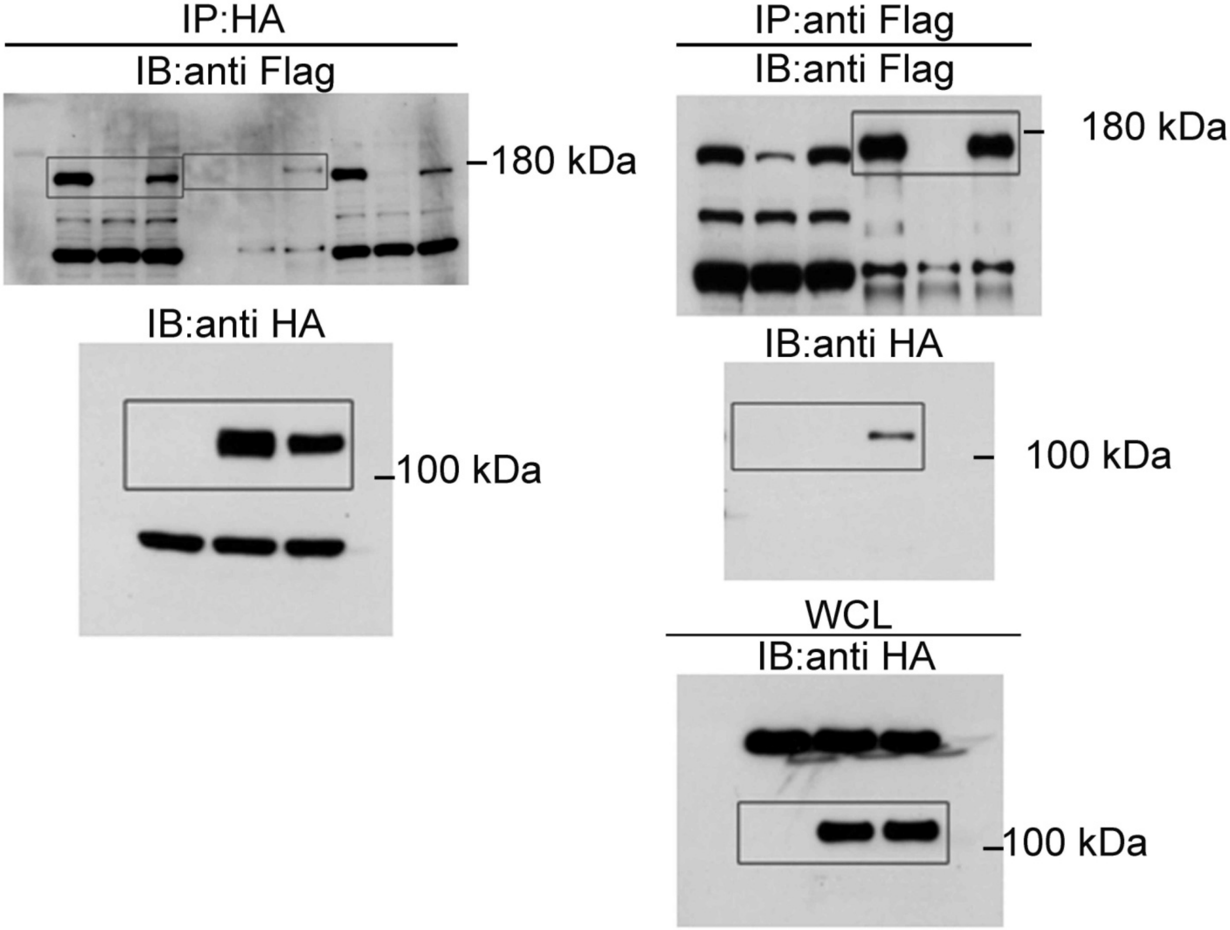
IP:HA
IB:anti Flag
180 kDa
IB:anti HA
100 kDa
IP:anti Flag
IB:anti Flag
180 kDa
IB:anti HA
100 kDa
WCL
IB:anti HA
100 kDa

Figure 7c

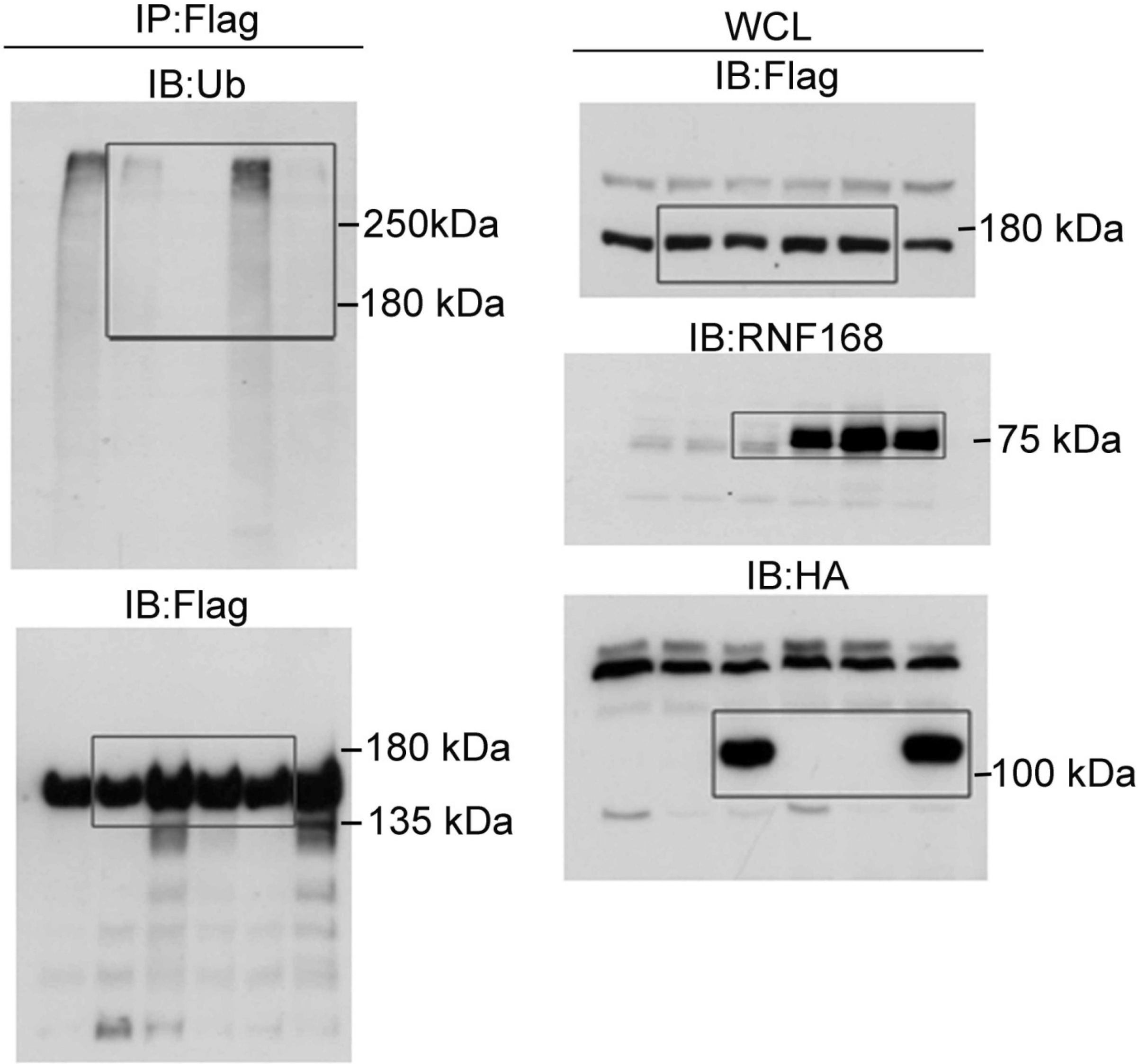
IP:Flag
IB:Ub
250kDa
180 kDa
IB:Flag
180 kDa
135 kDa
WCL
IB:Flag
180 kDa
IB:RNF168
75 kDa
IB:HA
100 kDa

Figure 7d

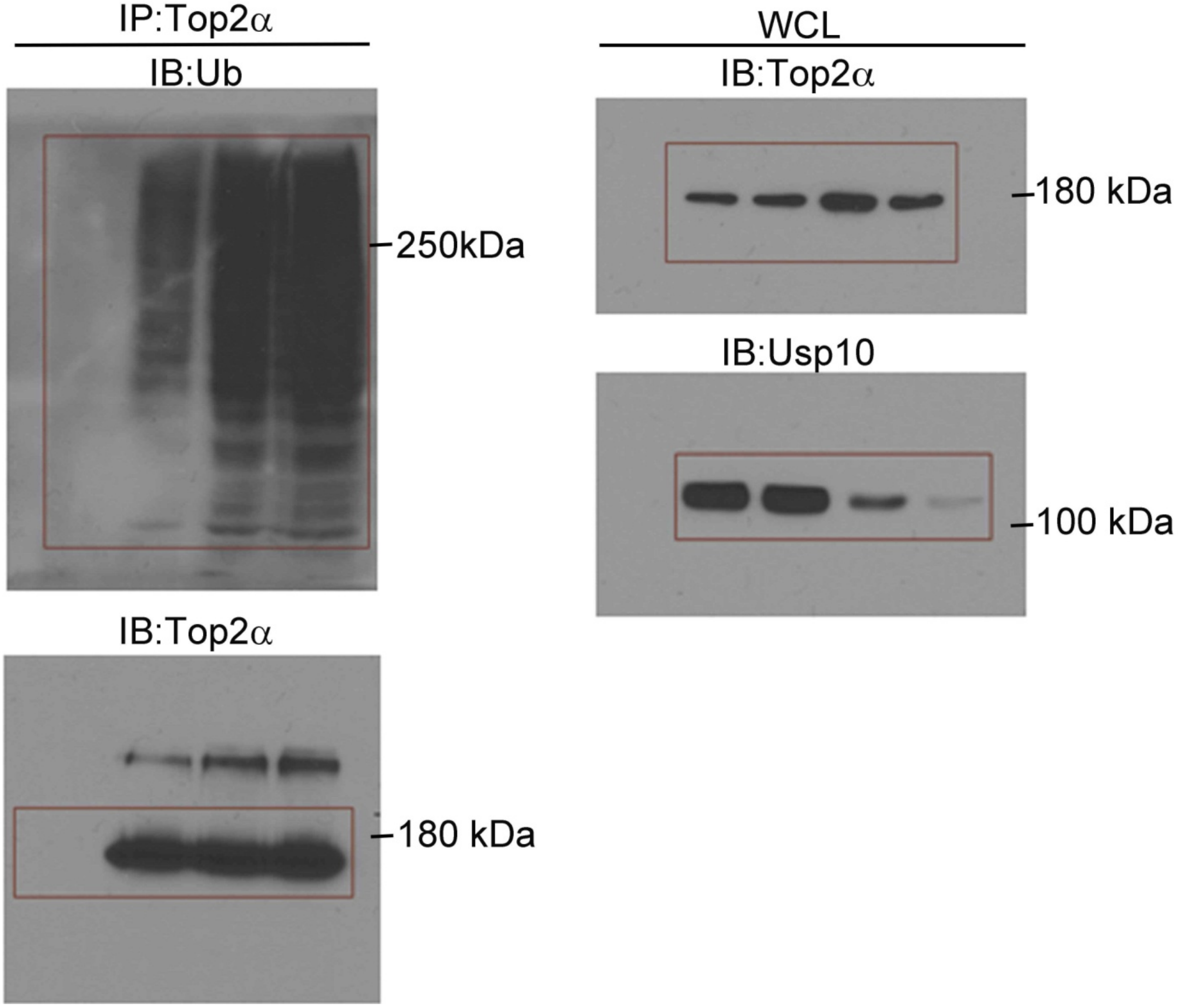
IP:Top2α
IB:Ub
250kDa
IB:Top2α
180 kDa
WCL
IB:Top2α
180 kDa
IB:Usp10
100 kDa

Figure 7e

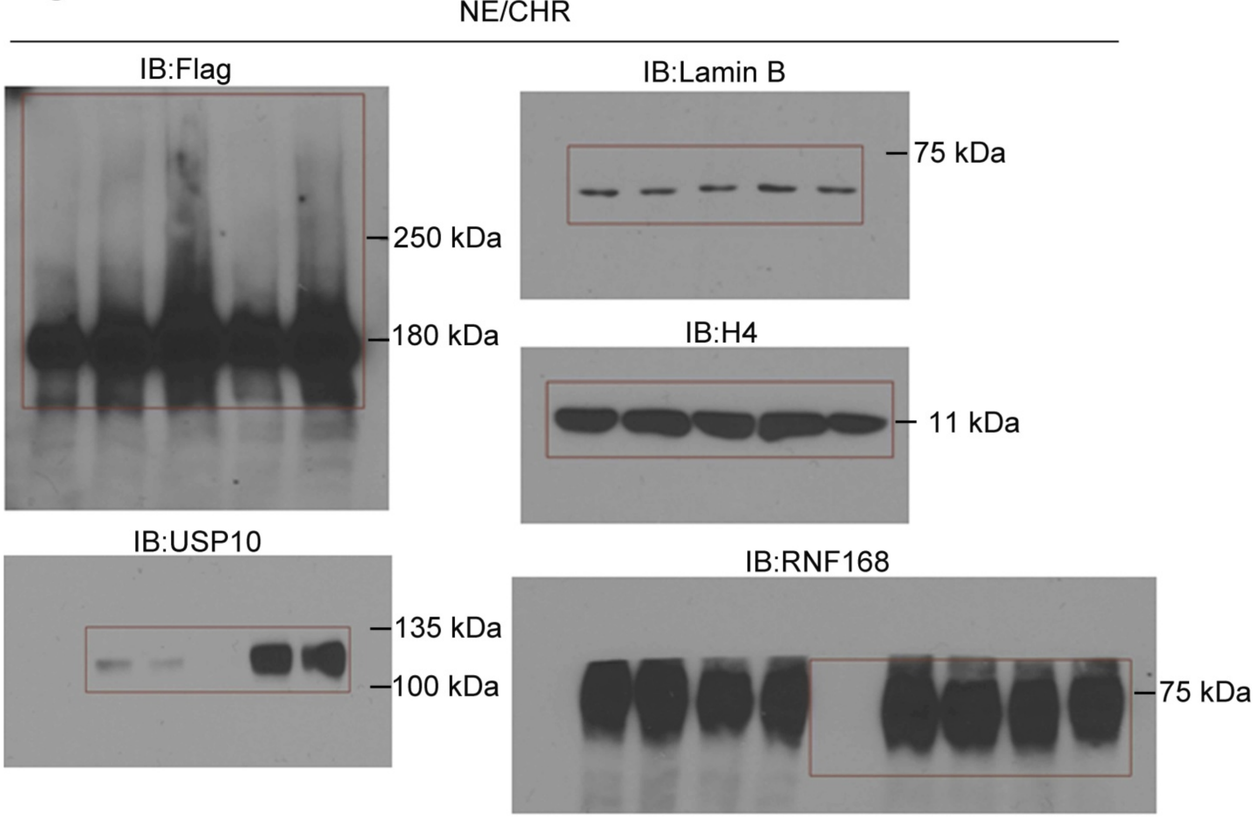

Supplementary Figure 2

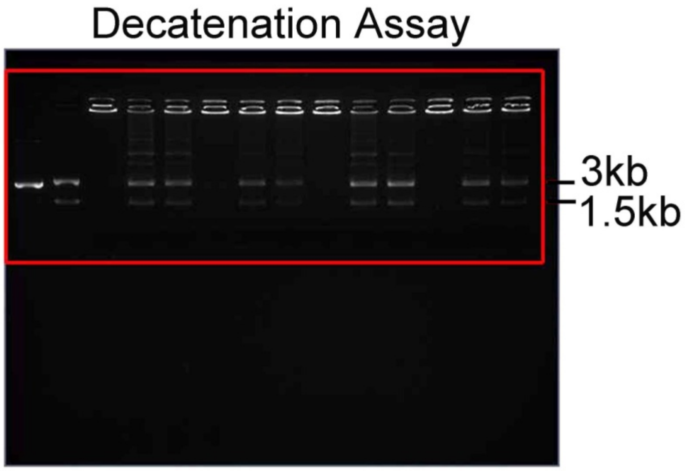

Supplementary Figure 6a

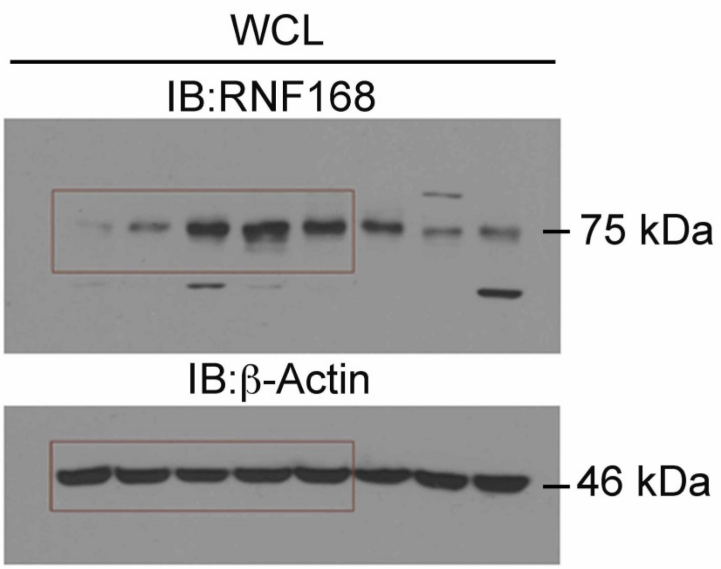

Supplementary Figure 7a

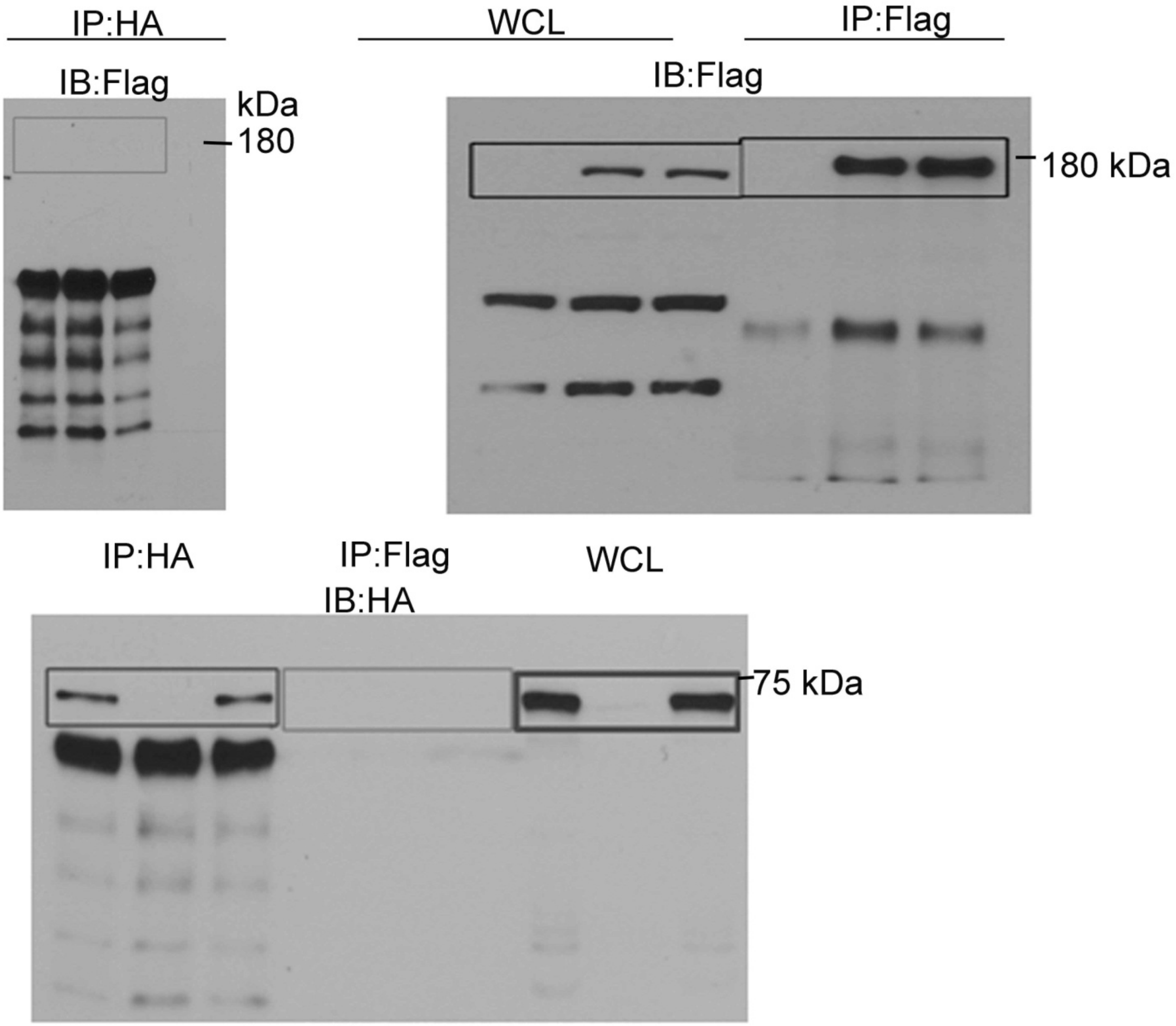

Supplementary Figure 7b

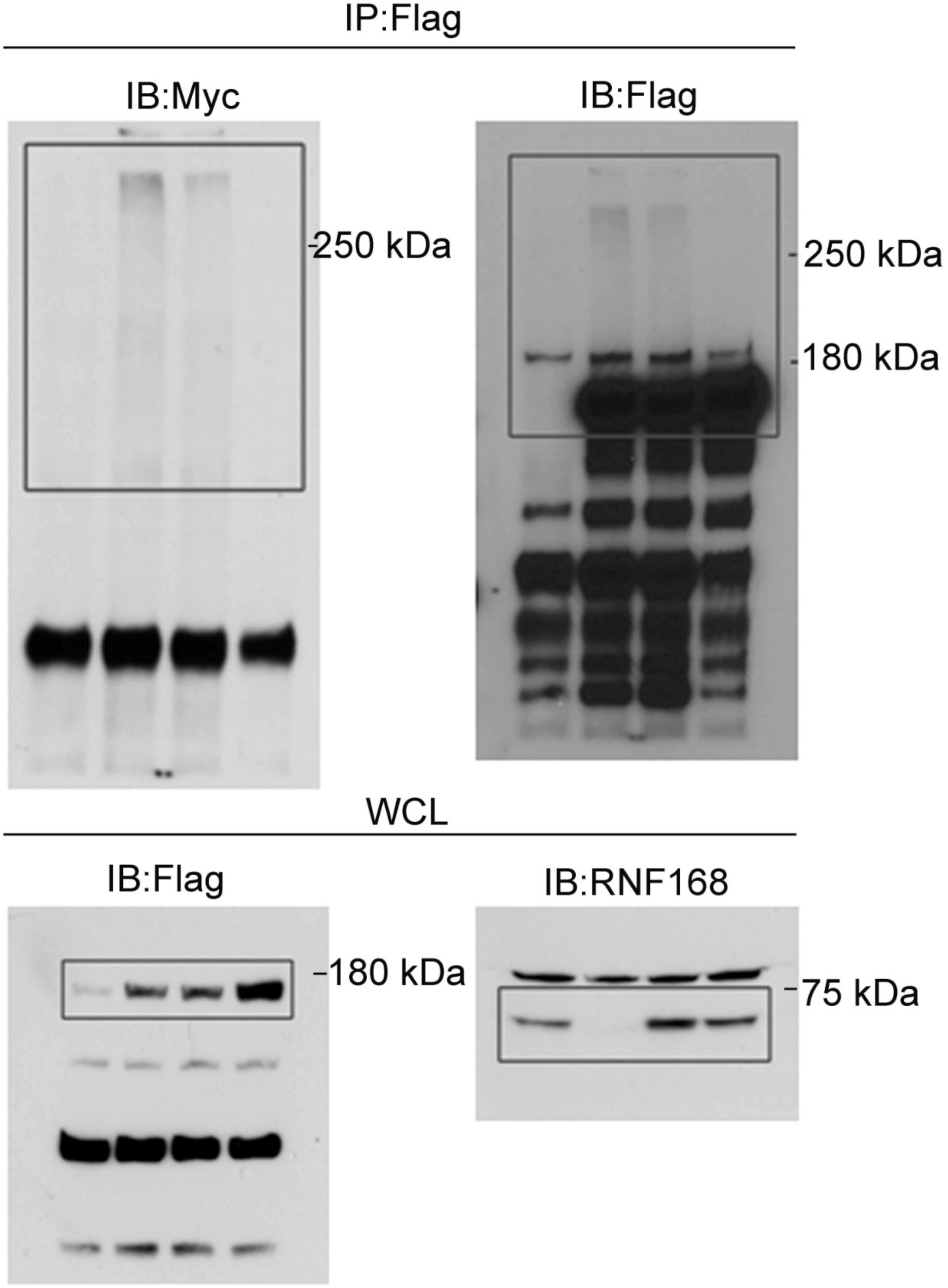

Supplementary Figure 7c

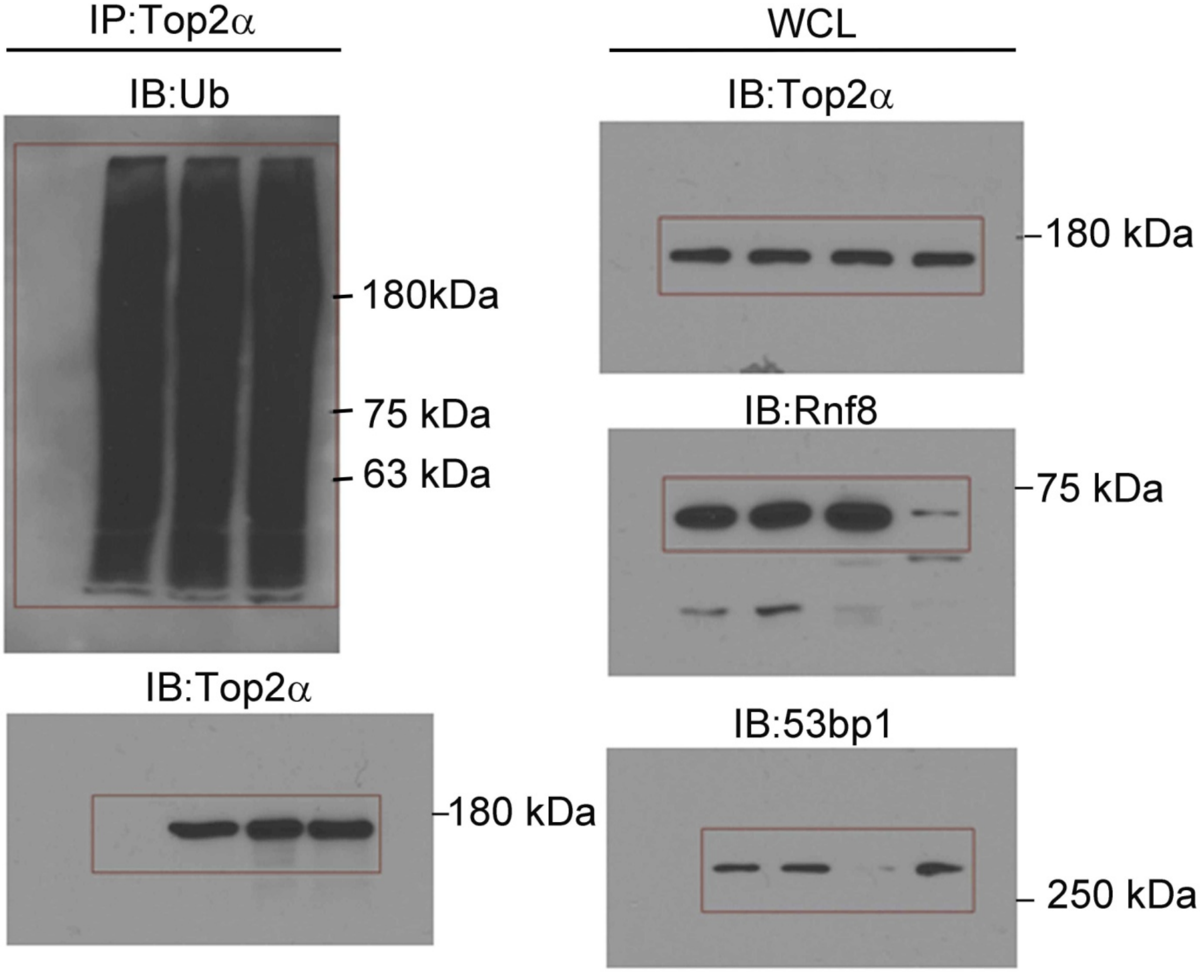

Supplementary figure 7d

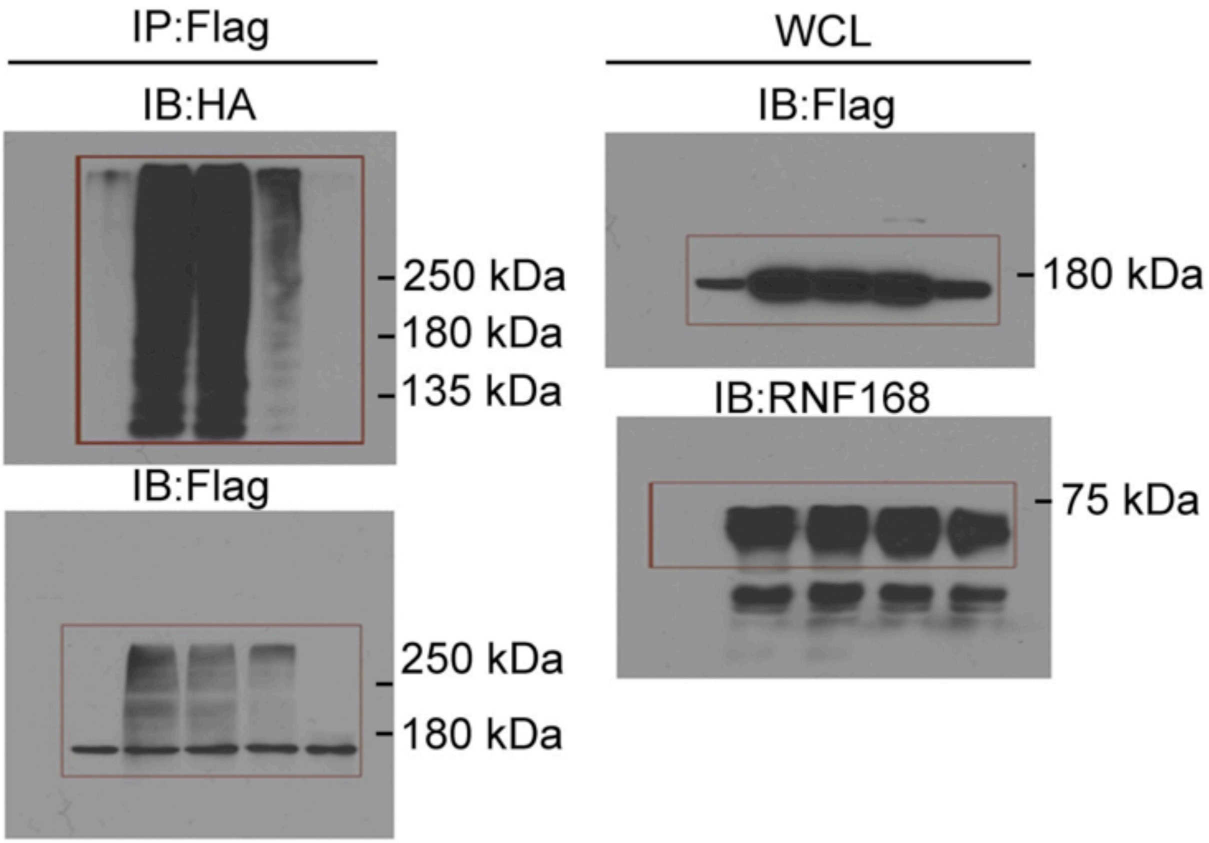

Supplementary figure 7e

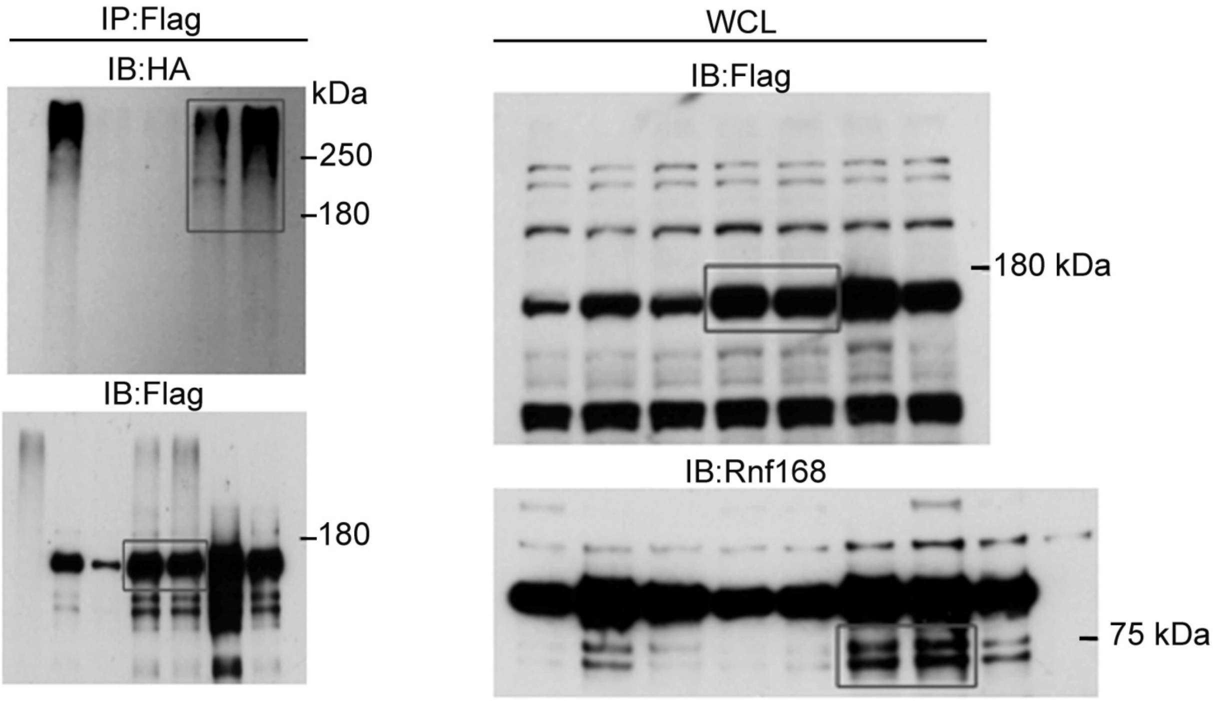

Supplementary Figure S12

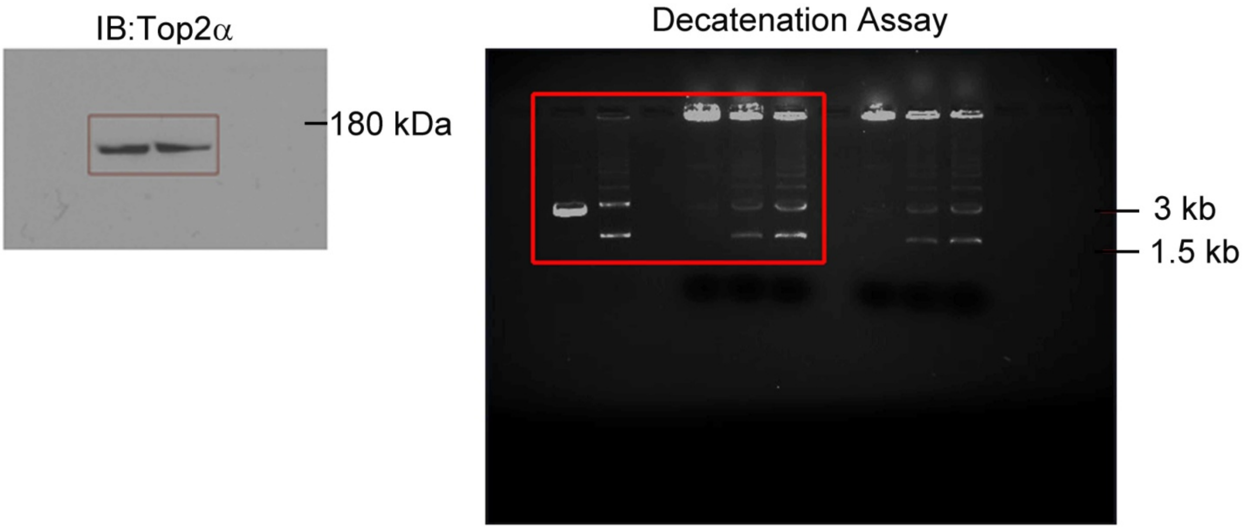

Supplementary Figure 13a

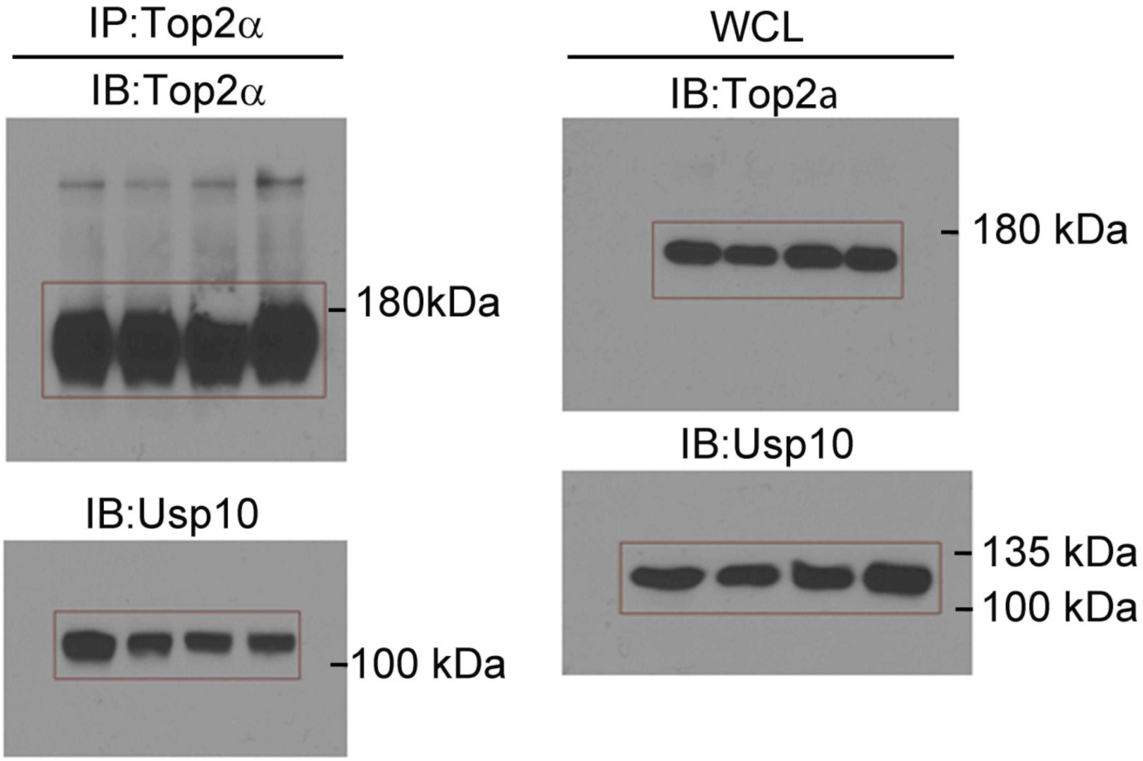

Supplementary Figure 13b

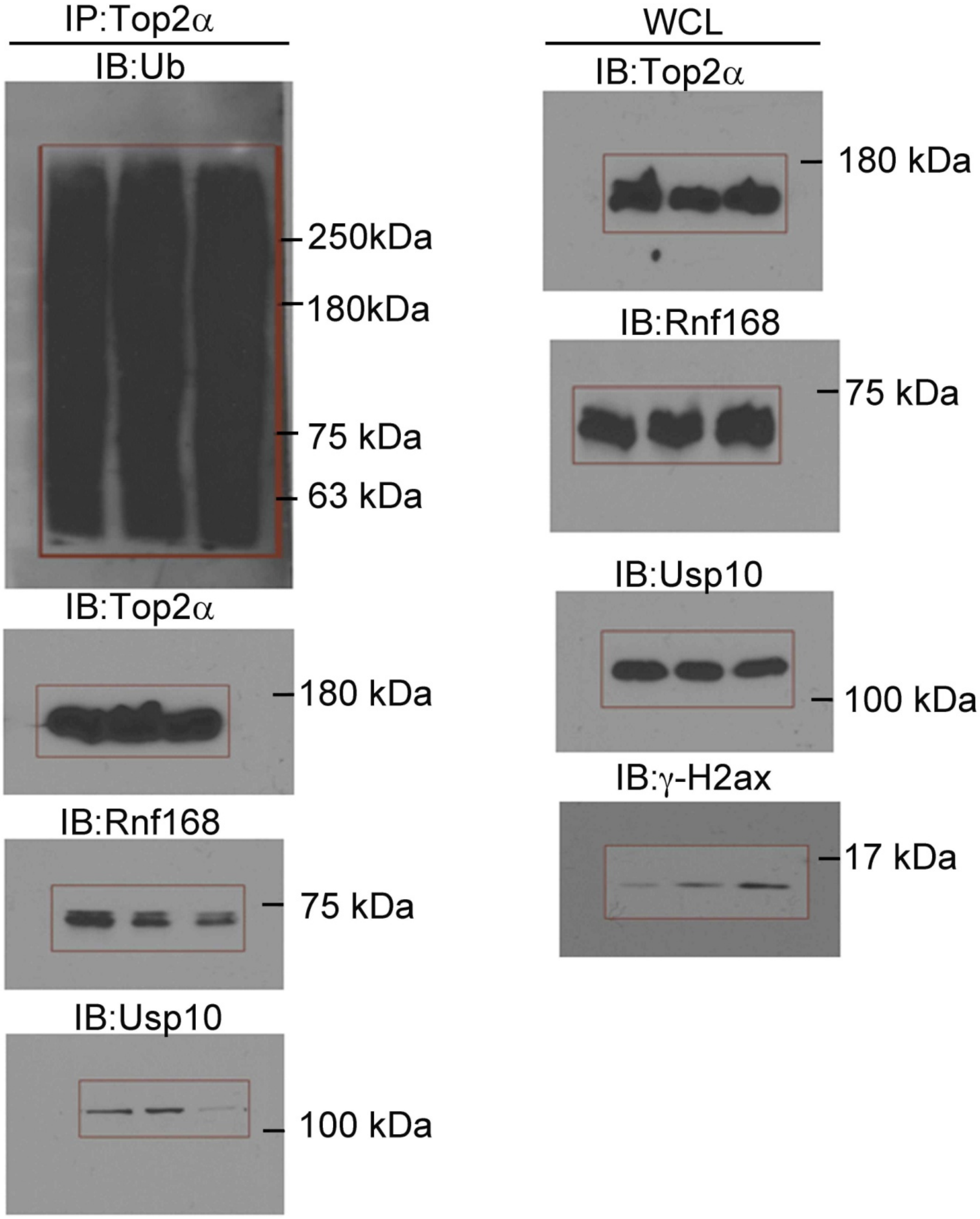

Gene ID	Gene Name	Protein ID	Mock chunkA	Mock chunkB	RNF168 chunkA	RNF168 chunkB
10419	PRMT5	20070220		83		66
8241	RBM10	325120986		24		24
26986	PABPC1	46367787		15		20
10521	DDX17	148613856		10		24
54726	OTUD4	156630992		8		
10642	IGF2BP1	56237027		7		21
4288	MKI67	103472005			82	
7341	SUMO1	4507801			73	
**7314**	**UBB**	**11024714**			**34**	**77**
**7153**	**TOP2A**	**19913406**			**11**	
7082	TJP1	116875765			10	
5108	PCM1	134142826			9	
22984	PDCD11	70980549			9	
**165918**	**RNF168**	**31377566**			**9**	**35**
4691	NCL	55956788				88
4670	HNRNPM	14141152				53
9188	DDX21	50659095				43
26064	RAI14	224809468				33
51474	LIMA1	165905589				31
1660	DHX9	100913206				29
1665	DHX15	68509926				28

1627	DBN1	18426913				14
8761	PABPC4	208431833				11
**9100**	**USP10**	**119220605**				**6**

Supplementary Table 1. Mass spectrometry analysis of RNF168 interacting partners. Raw and filtered list of protein spectral counts identified with a TPP probability of 1.00 in mock or FLAG-RNF168 expressing cells. Gene names, NCBI gene and protein ID along with observed spectral counts in each gel chunk are given for each protein.
